# Consensus-based guidelines for the provision of palliative and end-of-life care for people living with epidermolysis bullosa

**DOI:** 10.1186/s13023-023-02870-8

**Published:** 2023-09-04

**Authors:** Mark P. Popenhagen, Paola Genovese, Mo Blishen, Dilini Rajapakse, Anja Diem, Alex King, Jennifer Chan, Eduard Pellicer Arasa, Simone Baird, Anna Carolina Ferreira da Rocha, Gideon Stitt, Kellie Badger, Vlasta Zmazek, Faiza Ambreen, Caroline Mackenzie, Harper Price, Toni Roberts, Zena Moore, Declan Patton, Paul Murphy, Kattya Mayre-Chilton

**Affiliations:** 1 Department of Anesthesiology B090, Children’s Hospital Colorado, University of Colorado School of Medicine, Anschutz Medical Campus, 13123 E 16Th Ave, Aurora, CO 80045 USA; 2 Section of Pediatric Anesthesiology, Children’s Hospital Colorado, Aurora, CO USA; 3 Phoenix Children’s Hospital, Phoenix, AZ USA; 4 DEBRA New Zealand, Newtown, Wellington New Zealand; 5 Great Ormond Street Hospital Trust, London, UK; 6 EB House Austria, Department of Dermatology and Allergology, University Hospital of the Paracelsus Medical University, Salzburg, Austria; 7 Human Sense, LLC, Phoenix, AZ USA; 8 Lucile Packard Children’s Hospital, Stanford, Menlo Park, CA USA; 9 Sant Joan de Déu Barcelona Hospital, Barcelona, Spain; 10 DEBRA Australia, Pittsworth, QLD Australia; 11 Melbourne, Australia; 12 DEBRA Brazil, Blumenau, Santa Catarina Brazil; 13 Santa Catarina, Brazil; 14 Division of Clinical Pharmacology, University of Utah, Salt Lake City, UT USA; 15 DEBRA Croatia, Zagreb, Croatia; 16 Zagreb, Croatia; 17 DEBRA Pakistan, Lahore, Punjab Pakistan; 18 London, UK; 19 Guys and St Thomas’ Foundation NHS Foundation Trust, EB Adult Service, East Hampshire, England, UK; 20 DEBRA South Africa, Western Cape, Cape Town, South Africa; 21 Cape Town, South Africa; 22 Royal College of Surgeons in Ireland, University of Medicine and Health Sciences, Dublin, Ireland; 23 DEBRA International, Vienna, Austria; 24 Mildmay Mission Hospital, London, UK

**Keywords:** Epidermolysis bullosa, Palliative care, End-of-life, Quality-of-life, Clinical practice guidelines, Consensus guidelines

## Abstract

**Background:**

Inherited epidermolysis bullosa (EB) is a cluster of rare, genetic skin and mucosal fragility disorders with multi-system and secondary effects, in which blistering and erosions occur in response to friction/mechanical trauma. Considering the incurable and potentially life-limiting nature of the condition and the challenges posed by its symptoms, a palliative approach to EB-related care is necessary. However, knowledge and experience related to the provision of EB palliative care is minimal. Evidence-based, best care guidelines are needed to establish a base of knowledge for practitioners to prevent or ease suffering while improving comfort at all stages of the illness, not just the end of life.

**Methods:**

This consensus guideline (CG) was begun at the request of DEBRA International, an international organization dedicated to improvement of care, research, and dissemination of knowledge for EB patients, and represents the work of an international panel of medical experts in palliative care and EB, people living with EB, and people who provide care for individuals living with EB. Following a rigorous, evidence-based guideline development process, the author panel identified six clinical outcomes based on the results of a survey of people living with EB, carers, and medical experts in the field, as well as an exhaustive and systematic evaluation of literature. Recommendations for the best clinical provision of palliative care for people living with EB for each of the outcomes were reached through panel consensus of the available literature.

**Results:**

This article presents evidence-based recommendations for the provision of palliative healthcare services that establishes a base of knowledge and practice for an interdisciplinary team approach to ease suffering and improve the quality of life for all people living with EB. Any specific differences in the provision of care between EB subtypes are noted.

**Conclusions:**

Because there is yet no cure for EB, this evidence-based CG is a means of optimizing and standardizing the IDT care needed to reduce suffering while improving comfort and overall quality of life for people living with this rare and often devastating condition.

**Supplementary Information:**

The online version contains supplementary material available at 10.1186/s13023-023-02870-8.

## Background

Inherited epidermolysis bullosa (EB) is a devastating, incurable, and potentially life-limiting cluster of rare (~ 11.1 per 1 million live births), heterogeneous, genetic skin and variable mucosal fragility disorders with multi-system and secondary effects, in which blistering and erosions occur in the skin and mucosae in response to friction/mechanical trauma. Classically, EB has been classified into four types based on the level of skin cleavage or blister: EB Simplex (EBS), Dystrophic EB (DEB), Junctional EB (JEB), and EB Kindler (EBK), with autosomal dominant and recessive forms. Each group is further classified based on severity, disease findings and genes involved [1].

Although EB primarily involves the skin, several subtypes, especially the most severe, have systemic complications. These complications can include cutaneous squamous cell carcinomas (SCC), growth retardation, cardiomyopathies, musculoskeletal deformities, osteoporosis, anaemia, dental complications, infections, endocrine and psychological complications. Extracutaneous manifestations in EB patients can involve other epithelial tissues including the external eye, genitourinary and gastrointestinal tracts, and upper airway [2].

Palliative care as defined by the World Health Organization (WHO) is “an approach that improves the quality of life of patients (adults and children) and their families who are facing problems associated with life-threatening illness. It prevents and relieves suffering through the early identification, correct assessment and treatment of pain and other problems, whether physical, psychosocial, or spiritual” [3]. This approach to care is intended to ease suffering and improve comfort and quality of life (QoL) for the patient and their family, can begin as soon as a diagnosis is made or at any other stage of an illness [4–6]. Although end-of-life (EoL) care and referral to hospice programs are two of the more commonly known aspects of palliative care, they represent only a small part of the overall approach to care in the palliative care spectrum [4, 6]. Because of the incurable nature of EB, the challenges posed by its symptoms, the focus of treatment on easing suffering while improving comfort at all stages of the illness, and to meet the needs of people living with EB from birth to end-of-life, the authors took the most inclusive approach to palliative care in formulation of these guidelines and this article will consider all EB-related care to be palliative. Unfortunately, knowledge and experience related to the provision of EB palliative care is minimal.

### Scope and purpose

This article summarizes the clinical practice recommendations reached by an international panel of experts following a systematic literature review of the provision of palliative care in people living with EB. The overall purpose of this guideline is to provide the most up-to-date evidence and consensus-based recommendations for the provision of palliative care to improve outcomes and QoL for those living with EB and those individuals that support them. The intent of the guideline is to:

- examine and appraise all relevant literature up to June 2022,
- address clinical questions related to the provision of palliative care for people living with EB, and
- provide recommendations for future research.

### Objectives

- To provide guidance on the highest level of effective, culturally appropriate, and evidence-based palliative care treatment across the lifespan to people living with EB, their carers, and all healthcare providers involved in their care.
- To identify knowledge gaps and encourage future research in palliative care treatments for people living with EB.

### Guideline users

These Guidelines are intended to be used by, and to aid the learning and educational process for, all medical providers who are part of an interdisciplinary EB team (IDT), especially those without a palliative care expert or access to extended support of EB specialists in their area. These team members may include physicians, nurses, physician’s assistants, nurse practitioners, psychologists, counsellors, social workers, occupational therapists, physical therapists/physiotherapists, dietitians, and other medical team members. The Guidelines are also intended to be useful for people living with EB as well as their families, partners, carers, and community.

### Target group

Unless specifically stated, these guidelines may be applied by support services for all people of any age who have been diagnosed with any type of EB and should be considered on a case-by-case basis using the medical providers’ best clinical judgement in collaboration with the person living with EB and/or his/her family/carers. Most of the available literature did not define the type of EB and when specific subtypes were defined, the majority were on RDEB and JEB severe. Therefore, any specific recommendations made for any specific EB type(s) or subtype(s) herein may be applicable for other subtypes in certain circumstances only but may not necessarily be able to be generalized to other types, subtypes, or individuals. Any attempt to do so is at the clinical discretion of the individual, should be based on the latest available research, and is not the recommendation of any of the authors, their affiliate institutions, the panel, any of the DEBRA organizations, or DEBRA International.

## Methodology

The consensus guideline (CG) development process established three broad clinical questions.

A: Symptom management and survivorship

What are the best practices to manage the symptoms of, and improve survivorship for, EB over the lifespan?

B: Mental health, quality of life, and life satisfaction

Are there recommendations and interventions to improve mental health; QoL; and overall life satisfaction with respect to cultural, religious, and other belief-systems of people living with EB?

C: Grief

What are best practices to identify individuals who are grieving because of EB and to assist them in improving their ability to cope with that grief?

For further information on the CPG methodology, see Additional file 3.

## Results

**Fig. 1:**
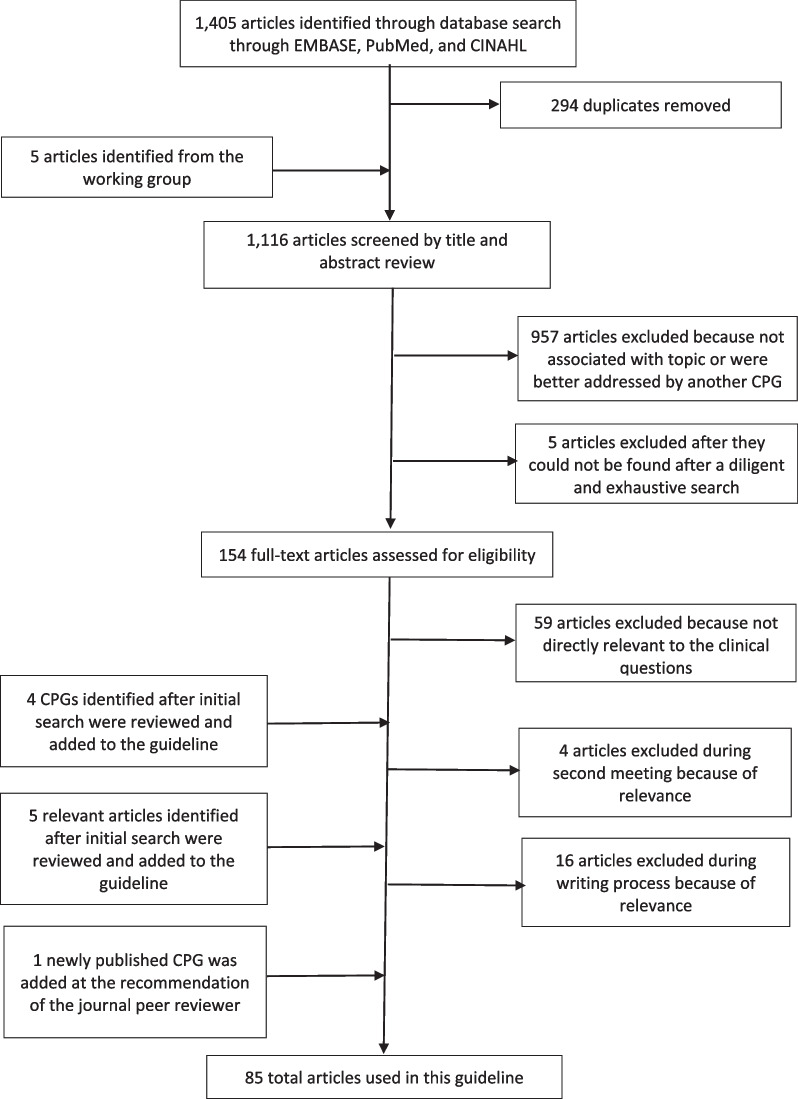
Flowchart of the systematic article review

In total, 1405 articles, were first identified, 85 articles were used in the qualitative synthesis of this CG. See Fig. 1 for an illustration of the selection and review process.

See Additional file 3 for the GRADEpro [7] “Summary of Findings Table” for the article summary data.

From the EB community survey, a total of 125 responses were obtained from people living with EB and/or their carers residing in at least 16 countries (not all respondents completed country information) spread over every continent except Antarctica. And 25 responses were obtained from healthcare providers from 14 disciplines in five countries.

### Recommendations

The three broad clinical questions that became the basis of this guideline are the result of responses to surveys distributed internationally to both Patient Population Involvement (PPIs; people living with EB or their carers) and healthcare providers (see Additional files 1 and 2). An exhaustive literature search identified 1111 papers of potential relevance, 84 of which were selected for appraisal, grading, and used here. One article, published in early 2023 was recommended by the journal peer reviewer and was subsequently added. The recommendations were unanimously agreed upon by all the panel members of the CG. Recommendation strength was strongly influenced by expert panel decision-making, which accounts for observable gaps between evidence levels and recommendation strength. The panel is also aware of the lack of strength of some of the recommendations due to the lack of high-certainty evidence. The evidence level is very low for all recommendations. (See Table 1 for additional information on the wording used for, and strength of, each recommendation.) Good practice point (GPP) recommendations are best practices based on the clinical experience of the panel. The recommendations for each of the clinical questions are outlined in Tables 2, 3 and 4 and are sub-grouped by topic.

**Table 1:** GRADE strength of recommendations ratings [89]

Strength	Wording	Symbol	Definition
Strong recommendation for the use of an intervention	‘Offer’ (or similar, e.g., ‘use’, ‘provide’, ‘take’, ‘investigate’, etc.)	↑↑	Benefits of the intervention outweigh the risks; most patients would choose the intervention while only a small proportion would not; for clinicians, most of their patients would receive the intervention; for policymakers, it would be a useful performance indicator
Weak recommendation for the use of an intervention	‘Consider’	↑	Risks and benefits of the intervention are finely balanced; many patients would choose the intervention, but many would not; clinicians would need to consider the pros and cons for the patient in the context of the evidence; for policymakers it would be a poor performance indicator where variability in practice is expected
No recommendation		Θ	Insufficient evidence to support any recommendation
Strong recommendation against the use of an intervention	‘Do not offer’	↓↓	Risks of the intervention outweigh the benefits; most patients would not choose the intervention while only a small proportion would; for clinicians, most of their patients would not receive the intervention

Good Practice Points (GPP)

GPP—recommended best practice on the clinical experience of the guideline development group

(Ratings table taken from Greenblatt et al. [89] “Recommendations on pregnancy, childbirth and aftercare in epidermolysis bullosa: a consensus-based guideline”)

**Table 2:** Recommendations table: symptom management and survivorship

No.	Recommendation	Strength of recommendation	Key references*
*General*			
R1	EB is a disease that is best treated within an IDT that optimally consists of the person living with EB and their carers at the core in addition to a dermatologist, paediatrician/primary care physician, specialist nurse, wound care specialist, surgeon, oncologist, psychologist, pain specialist, palliative care specialist, gastroenterologist, physiotherapist, occupational therapist, and a social worker. Each team member’s role is clearly established shortly after the birth of a baby with EB and are adjusted to meet the child’s evolving needs	↑↑	[8, 9, 11–19] → [9, 10]
R2	Treatment should focus on achievable goals over the span of an entire lifetime, with the goal of managing physical and/or emotional suffering while respecting the autonomy and individuality of each person, and providing psychoeducation to the person living with EB, their family, and carers in a way they can fully understand	↑↑	[16, 19–21]
R5	It is paramount to fully inform the patient of all their treatment options (including no treatment) in an age-appropriate manner and to ensure that all the wishes of the person living with EB and their family are heard and respected, including those related to where they wish to die, the level of sedation that they are willing to tolerate, as well as feeding, and hydration	↑↑	→ [9]
R29	Accurately diagnosing the EB type and applying the appropriate interventions to manage disease complications significantly improves disease management and the likelihood of survival as the patient ages	↑↑	[18, 31]
R30	Consider offering nursing care to give carers respite	↑	[11, 46]
R31	To provide the best possible care, good provider mental health through self-care practices and a life outside of the medical setting is necessary	↑↑	[10, 20, 47, 48]
R32	Parents and their affected child must be allowed the opportunity to voice their opinions regarding the best plan of care	↑↑	[16, 20, 48, 49]
R33	A collaborative “co-survivor” approach between healthcare providers and parents is critical for educating patients on the importance of self-care and for developing better palliative care protocols	GPP	
R34	A multifactorial approach is recommended to assess the balance between QoL and survivorship	GPP	
R35	Comorbidities should be diagnosed and treated early whenever possible	↑↑	[54]
*Pain and symptom management*			
R2	See R2 above	↑↑	[16, 19–21]
R3	Provide appropriate levels of analgesia to keep pain controlled while using the safest possible routes	↑↑	→ [9]
R6	Steps should always be taken to manage suffering related to pain and itch with the understanding that the aggressiveness of pharmacologic treatment of pain and itch may increase/change as the patient’s goals and clinical trajectory change	↑↑	[20, 27]
R7	Treatment should focus on identifying different pain qualities to plan the most effective and appropriate pharmacologic and non-pharmacologic pain management regimen tailored for the individual and their situation	↑↑	→ [10] [28–30]
*Urology*			
R4	Urinary catheterization and administering nasogastric or subcutaneous fluids toward the end of life may be beneficial if they do not outweigh discomfort and patients at this stage may be more comfortable having fewer or even no dressing changes	GPP	→ [9]
R8	Urological complications should be detected and treated early	↑↑	[31]
*Ophthalmology*			
R9	Ocular involvement, especially management of corneal abrasions and corneal epithelial defects must be considered in all cases of EB	↑↑	[32]
R10	The panel recommends the preventative use of artificial tears to reduce corneal abrasions and to improve eye comfort	GPP	
*Gastroenterology/nutrition*			
R11	“The aims of nutritional support mainly include: improving nutritional status, alleviating the stress of oral feeding, and minimizing nutritional deficiencies”	↑↑	[2]
R12	Because of the particularly deleterious nature of GER, prompt treatment is necessary	↑↑	[2]
R13	Dietary modifications, such as choosing foods that are energy dense (e.g., fats), softer, and have lower volume, are recommended for strictures that are less severe and esophageal dilation is necessary for severe strictures	↑↑	[2, 34, 35]
R14	Nasogastric tubes are not recommended	↓↓	[2]
R15	G-tube placement is recommended for patients who present with failure to thrive, chronic oral issues, chronic constipation, and/or high stress associated with feeding despite the potential risks	↑↑	[40, 41]
R16	G-tube placement needs to be carefully managed to minimize side effects and potential downstream negative gastrointestinal effects	↑↑	[23, 34]
R17	People affected by more severe forms of EB may need to rely heavily upon increased amounts of micronutrients and vitamins through nutritional supplementation	↑↑	[2]
R18	Infants with more damaged skin may require energy supplementation powders to be added to expressed breast milk/infant formulas so that they may achieve their nutritional goals	↑↑	[2]
R19	Children living with EB may begin consuming solid foods at the same time as unaffected children (i.e., when good head control is achieved), but hard, sharp, or otherwise rough foods are not recommended	↓↓	[2]
R20	Meals should contain the highest caloric and nutrient content with the lowest possible volume	↑↑	[2]
*Oral/dental*			
R21	The importance of good oral preventative care and cautious medication selection is strongly emphasized with the understanding that conventional dental management must typically be modified, and all dental care should, whenever possible, be done by a dentist experienced in the care of people living with EB	↑↑	[34]
R22	Before teeth are extracted, clinicians should consider the difficulties of wearing prosthetic devices as well as the psychological effect losing teeth can have	↑	[34]
R23	Just as a close relationship between the dental care provider and dietitian is important, maximizing prevention methods and utilizing appropriate oral hygiene techniques and equipment are very important	↑↑	[34]
*End-of-life*			
R4	See R4 above	GPP	→ [9]
R24	After a diagnosis of severe JEB subtype is made, it is advisable to focus exclusively on comfort-oriented care	↑↑	[43]
R25	Enteral nutrition is not recommended in the context of severe JEB	↓↓	[2]
R26	Withholding medically non-beneficial interventions is well-established in both adult and paediatric medicine and is widely practiced in end-of-life care for people with EB	GPP	[20]
R27	To relieve refractory symptoms, palliative sedation may be considered	↑	[22]
R28	Providing pain medications with the intent of symptom management is well-established and is an alternative to ethical treatment of pain without resorting to euthanasia, even if doing so hastens death	↑↑	[20]

Recommendations are based on the results of the literature review. In addition, other recommendations relating to palliative care were added during the process of guideline development from expert consensus, and the experience of the guideline development group. To provide easier access to information, the recommendations in this table are grouped in accordance with the clinical questions and are arranged by clinical subheadings. If a recommendation fit within multiple subheadings or within multiple clinical questions, it was listed in each. Recommendations were not listed in order of strength or importance. Recommendation strength was strongly influenced by expert panel decision-making, which accounts for observable gaps between evidence levels and recommendation strength. The evidence level is very low for all recommendations. For the strength of recommendation ratings see Table 1. EB, epidermolysis bullosa; GPP, good practice point; G-tube, gastrostomy feeding tube; JEB, junctional epidermolysis bullosa; IDT, interdisciplinary team; QoL, quality-of-life; RDEB, recessive dystrophic epidermolysis bullosa; SCC, squamous cell carcinoma. *Right-pointing arrows (→) denote a guideline document

### A: Symptom management and survivorship

**Recommendation 1 (R1)** ↑↑ EB is a disease that requires the timely involvement of different specialties within an interdisciplinary team (IDT) that is an essential component in the effective and inclusive delivery of palliative care services and support. This team optimally consists of the person living with EB and their carers at the core in addition to a dermatologist, pediatrician/primary care physician, specialist nurse, wound care specialist, surgeon, oncologist, psychologist, pain specialist, palliative care specialist, gastroenterologist, physiotherapist, occupational therapist, and a social worker whose roles are clearly established shortly after the birth of a baby with EB and are adjusted to meet the child’s evolving needs [8–16]. This team works to improve survivorship by managing the multiple and very complex symptoms of the disease through improvement of patient/carer skills, knowledge, and motivation, with the eventual goal of independent patient self-care [9, 16–19]. This section seeks to identify the best practices to manage the symptoms of, and improve survivorship for, EB across the lifespan. See Table 2 for a summary of the recommendations for symptom management and survivorship.

### A.1 Symptom control/management

Palliative care focuses on the balance between the burdens of any intervention provided within the context of the patient’s goals and quality of life. **R2** ↑↑ Because EB is a disease with lifelong symptoms, treatment should focus on achievable goals over the span of an entire lifetime, with the goal of managing physical and/or emotional suffering while respecting the autonomy and individuality of each person, and providing psychoeducation to the person living with EB, their family, and carers in a way they can fully understand [16, 19–21].

As the end of life approaches, involving multiple resources is as important as ever, especially because EoL care does not follow traditional cure-oriented pathways [22]. **R3** ↑↑ It is important to provide appropriate levels of analgesia to keep pain controlled while using the safest possible routes [9]. **R4 Good Practice Point (GPP)** Urinary catheterization and administering nasogastric or subcutaneous fluids at EoL may be beneficial for issues such as wound management, pain with movement, and maintaining clean dressings as long as they do not outweigh discomfort. Similarly, patients at EoL may be more comfortable with fewer dressing changes or may even wish to forgo them completely [9]. **R5** ↑↑ Whenever possible, it is paramount that the patient is fully informed of all their treatment options (including the option of no treatment) and potential complications, in an age-appropriate manner and that all of the wishes of the person living with EB and their family are heard and respected, including those related to where they wish to die (e.g., home or hospital setting), the level of sedation that they are willing to tolerate, as well as feeding, and hydration [9, 10, 23, 24].

### Wound care

Wound formation and management are at the core of this disease and of patient suffering [8]. For a thorough examination of the wound care management recommendations for people living with EB, the panel directs the reader to the 2017 Best practice guidelines for skin and wound care in epidermolysis bullosa [25].

### Pain and Itch management

For a thorough examination of the pain management recommendations for people living with EB, the panel directs the reader to the 2014 Pain care for patients with epidermolysis bullosa: best care practice guidelines [26]. We include here recommendations published since the release of that CPG. **R6** ↑↑ Pain and itch remain an ever-present and difficult challenge for those who live with EB and their carers, and steps should always be taken to manage suffering with the understanding that the aggressiveness of pharmacologic treatment of pain and itch may increase/change as the patient’s goals and clinical trajectory change [2, 20, 26, 27]. **R7** ↑↑ Treatment should focus on identifying different pain qualities, which could be helpful for planning the most effective and appropriate pain management regimen that could include medication [28] and nonpharmacologic techniques (e.g., family psychotherapy, nonpharmacologic pain management skills, etc.) tailored for the individual and their situation [10, 29, 30].

### Urology

**R8** ↑↑ Early detection and treatment of urological complications due to recurrent blistering such as dysuria, recurrent urinary tract infections, hematuria, albuminuria, and urinary tract obstruction (including labial fusion) are important for improved survival and QoL [31].

### Ophthalmology

**R9** ↑↑ Ocular involvement, especially management of corneal abrasions and corneal epithelial defects must be considered in all cases of EB [32]. Use of bandage contact lens in EB patients suffering from corneal epithelial defects can immediately improve the pain and frequency of corneal abrasions without sequelae and can be used for years without secondary effects [33]. **R10 GPP** Additionally, the panel recommends the preventative use of artificial tears to reduce corneal abrasions and to improve eye comfort.

### Diet/nutrition

#### Clinical considerations

Several challenging aspects of nutrition management in EB necessitate close cooperation between IDT members, including: iron-deficiency anaemia, gastrostomy placement, feeding, micronutrients and vitamins, muscle mass and mobility, dental health, and bone density [34]. Nutritional deficits due to EB are caused by a hypercatabolic inflammatory state with increased metabolic needs that are often accompanied by reduced nutritional intake due to oral and gastrointestinal tract involvement such as blistering and erosion in the oral cavity, pharynx, larynx, and esophagus, ankyloglossia, microstomia, chewing difficulties, hiatal hernia, gastritis, peptic ulcer disease, inflammatory bowel disease, anal erosions and fissures, constipation, protein-losing enteropathy, and gastroesophageal reflux (GER) disease can also lead to a greater expenditure of nutritional energy than what the patient is able to ingest [2]. **R11** ↑↑ “The aims of nutritional support mainly include improving nutritional status, alleviating the stress of oral feeding, and minimizing nutritional deficiencies that consequently affect growth, pubertal development, bowel function, immune status, and wound healing” (p. 343) [2]. **R12** ↑↑ Because of the particularly deleterious nature of GER (causing esophagitis, nausea, pain, decreased appetite, severe chronic luminal blistering, development of esophageal strictures, dysphagia, and scarring leading to regurgitation of gastric content), prompt treatment is necessary [2].

Esophageal scarring not caused by GER (usually located in the upper esophagus), can cause strictures that gradually limit the textures of food that can be ingested to the point that patients may be unable to swallow their own saliva [2, 35]. Esophageal stricture management methodologies that have been proposed include, **R13** ↑↑ dietary modifications, such as choosing foods that are energy dense (e.g., fats), softer, and have lower volume, are recommended for strictures that are less severe [2, 34] and esophageal dilation for severe strictures [2]. Balloon dilation can be safely repeated without the risk of severe complications such as esophageal perforations or death [35]. For further discussion of procedural anesthetic management for people living with EB during esophageal dilatation, the panel refers the reader to the articles by Peterson et al. [36] and by Gottschalk, et al. [37].

For a thorough examination of the use of nutrition to manage and prevent constipation for people living with EB, the panel directs the reader to the 2019 Preventative Nutritional Care Guideline: Constipation Management for Children and Adults with Epidermolysis Bullosa (EB) [38].

#### Anaemia

In people living with EB, anaemia is caused by chronic blood loss, ulceration and desquamation of the gastrointestinal mucosa, reduced iron intake, and chronic inflammation leading to suppressed erythropoiesis [2]. It causes a significant disturbance on general well-being through negative impact on fatigue, anorexia, wound healing, ability to breathe, and tolerance to physical activity. For a thorough examination of the anaemia for people living with EB, the panel directs the reader to the 2023 Consensus guidelines for diagnosis and management of anemia in epidermolysis bullosa [39].

#### Gastrostomy feeding tube (G-tube) placement

There is significant variability in the symptoms associated with the different EB types in terms of the extent of blistering and the degree of other complications such as difficulties with the mouth and throat, kidney and liver issues, and skeletal muscle dysfunction, and additional complications of severe forms of EB can also lead to chronic malnutrition thereby negatively affecting growth, health, wound healing, and overall QoL [40, 41]. As the person living with more severe forms of EB ages and as their disease progresses, it becomes increasingly difficult to meet nutritional goals and sicker patients are not able to take in enough calories and nutrients to affect growth, even if their diet consists of high protein and energy foods with oral supplementation. Unfortunately, although there is a direct correlation between disease severity and the patient’s nutritional and caloric needs [41], strategies to supplement oral nutritional intake are fraught with challenges and are difficult to maintain adequately.

Because of continual patient and parental concerns surrounding oral intake of food, liquids, medications, and supplements, as well as growth failure, patients, parents, and providers must often contemplate the use of enteral feeds. **R14** ↓↓ Nasogastric tubes are not recommended because of the risk of internal and external trauma and the difficulties securing them to the skin of the face [2]. **R15** ↑↑ G-tube placement has become increasingly routine in EB centers worldwide over the past 20 years and is recommended for patients who present with failure to thrive, chronic oral issues, chronic constipation, and/or high stress associated with feeding despite the potential risks [40, 41]. **R16** ↑↑ Although G-tube utilization may prolong survivorship by improving growth and weight gain, they must be carefully managed to minimize side effects associated with placement and potential downstream negative gastrointestinal effects that can significantly affect patient outcomes and QoL [23, 34]. Improved nutrition results in increased enjoyment of eating, thereby improving the QoL of the persons with EB [23]. G-tube-related complications in people living with EB did not differ from those observed in patients with other diseases [40, 41] and include pain, abdominal distension, leakage, and infection around the insertion site [40]. In two studies [34, 40], patients and parents rated their overall satisfaction with G-tube feeding as high, despite the issues related to pain and leakage. Those reporting severe problems experienced a substantial effect on their everyday lives. Despite this, parents unanimously acknowledged that when G-tube feeds were the only means to feed and medicate their children, they were willing to tolerate the problems rather than have their children endure uncontrollable hunger and pain.

#### Micronutrients and vitamins

Deficiencies in micronutrients such as zinc, calcium, iron, and vitamins C; A; K; B1, 6, and 12; 25(OH)D3; niacin; selenium; and carnitine can occur in people living with EB because of insufficient intake, chronic inflammation, and losses due to blistering and can potentially lead to the development of fatal cardiomyopathy [2]. **R17** ↑↑ Therefore, people affected by more severe forms of EB may need to rely heavily upon increased amounts of micronutrients and vitamins through nutritional supplementation.

#### Feeding

In newborns and infants, breast milk may satisfy nutritional requirements in those less severely affected by EB. **R18** ↑↑ However, infants with more damaged skin may require energy supplementation powders to be added to expressed breast milk/infant formulas so that they may achieve their nutritional goals [2].

**R19** ↓↓ Children living with EB may begin consuming solid foods at the same time as unaffected children (i.e., when good head control is achieved), but hard, sharp, or otherwise rough foods are not recommended [2]. **R20** ↑↑ Meals should contain the highest caloric and nutrient content with the lowest possible volume so that satiety is not reached before they are able to get all of the needed calories and nutrients, especially for children with small appetites or who are only able to ingest liquids [2].

#### Oral/dental health

There are several oral/dental features of EB including microstomia, ankyloglossia, trismus, progressive fixation of the tongue to the mouth floor, malocclusion, teeth that are structurally deficient and prone to decay, blistering and scarring, and gum disease [34]. Other risk factors for decay include xerostomia and jaw necrosis caused by medication side effects, GERD, poor dexterity, care difficulties, and poor dietary regimens [34]. **R21** ↑↑Good oral preventative care and cautious medication selection is strongly emphasized with the understanding that conventional dental management must typically be modified, and all dental care should, whenever possible, be done by a dentist experienced in the care of people living with EB. **R22** ↑ Before teeth are extracted, clinicians should consider the difficulties of wearing prosthetic devices as well as the psychological effect that losing teeth can have [34]. **R23** ↑↑ Finally, just as a close relationship between the dental care provider and dietitian is important, maximizing prevention methods and utilizing appropriate oral hygiene techniques and equipment are very important [34]. For additional recommendations for oral care management for people living with EB, the panel directs the reader to the 2020 Clinical practice guidelines: Oral health care for children and adults living with epidermolysis bullosa [42].

### EB specific considerations

#### JEB severe subtype

The sparse epidemiological data about severe JEB (formerly called JEB generalized severe [GS-JEB] or JEB Herlitz-type) that is available suggests that while aggressive interventions may possibly prolong life by months, they do not change the inevitable outcome of death during infancy. However, these extra months may be extremely important emotionally to the parents [20].

**R24** ↑↑ After a diagnosis of severe JEB subtype is made, it is advisable to focus exclusively on comfort-oriented care [43]. Despite the likelihood of eventual transition to comfort care,, upper airway interventions to improve QoL can be performed safely on people living with severe JEB, even though they will not affect the ultimate outcome. Therefore, the role of such intervention within the goals of care needs to be discussed with the family and primary health care team to choose the most appropriate plan of care [44]. **R25** ↓↓ Finally, because enteral nutrition is unsuccessful in severe JEB, it is not recommended [2].

Alternatively, the decision to withhold medical interventions becomes ethically permissible when the burdens of medically futile interventions outweigh any potential benefits to the patient. **R26 GPP** Withholding medically non-beneficial interventions at EoL is well-established in both adult and pediatric medicine and is widely practiced for people with EB [20].

#### Groningen protocol

The extreme suffering of an infant in the Netherlands with RDEB, led to the development of the Groningen Protocol in 2005. The protocol, meant to transparently identify candidates for neonatal euthanasia, medically requires, “(1) certainty of the diagnosis and prognosis; (2) presence of hopeless and unbearable suffering; (3) confirmation of the diagnosis, prognosis, and suffering by at least one doctor independent of the medical team; (4) parental consent; and (5) performance of the procedure in accordance with the accepted medical standard” (p. 448) [20]. Since the protocol’s inception, at least two infants with lethal forms of EB were euthanized at the request of their parents because of uncontrollable pain despite an optimized pain regimen [20, 43, 45]. “However, although the majority of parents appreciated being able to discuss about euthanasia transparently, ‘most never requested it as the suffering of their child could be adequately treated with palliative medicine’” (p. 448) [20].

One of the primary concerns with the Groningen Protocol is that because neonates cannot express their own autonomy or provide assent/consent for themselves, the decision to proceed with euthanasia must be made by the neonate’s surrogates. Furthermore, the judgment of whether the suffering is ‘unbearable’ is subjective and raises the questions of how suffering is defined, who may make the definition, and who may give consent to proceed. Similarly, the impossibility of certainty is also part of the debate related to medical futility [20]. **R27** ↑ Palliative sedation, another often debated option, has been shown to be effective in a patient with severe JEB for the reduction of refractory symptoms [22]. **R28** ↑↑ The principle of double effect (providing pain medications with the intent to treat uncontrolled pain even though it may unintentionally hasten death) is a well-established alternative and is considered to be an ethical treatment of pain without resorting to euthanasia. However, some infants still suffer with pain despite high doses of pain medications [20]. This subject is in no way settled, thereby necessitating further research and ethical debate that is beyond the scope of this article.

#### Squamous cell carcinoma

Whenever possible, the first line therapy for the treatment of SCC remains surgical intervention. In instances when surgery is not possible, other options may be discussed. For additional information on the treatment of SCC the the panel directs the reader to the 2016 Management of cutaneous squamous cell carcinoma in patients with epidermolysis bullosa: best clinical practice guidelines [9].

### A.2 Survivorship

### The importance of health care providers and carers in survivorship

Healthcare providers and carers play an important role in managing survivorship of people living with EB at different stages of their lives. **R29** ↑↑ Their ability to accurately diagnose the EB type significantly improves disease management and the likelihood of survival [18], as does applying the appropriate interventions to manage disease complications, especially as the patient ages [31]. **R30** ↑ Additionally, the option of nursing care can afford the primary caregiver(s)/parent(s)/co-survivor(s) respite allowing for an improved life balance and survivorship for the person living with EB and their family [11, 46].

**R31** ↑↑ Good provider mental health, achieved through self-care practices and a life outside of the medical setting, is necessary so that they may provide the best possible care from the time of diagnosis, through EoL, and possibly beyond [10, 20, 47, 48].

**R32** ↑↑ Although parents cannot change the outcome of an illness alone, they and their child must be allowed the opportunity to voice their opinions regarding the best plan of care. A close working relationship with the healthcare team enables parents to empower their child to use their skills and knowledge to promote and manage self-care [16, 20, 48, 49]. **R33 GPP** A collaborative “co-survivor” approach between healthcare providers and parents is critical for educating patients about the importance of self-care and for developing better palliative care protocols that improve outcomes and survivorship.

### The balance of quality of life and survivorship in EB

Assessment scales that measure the QoL of the person living with EB offer a more objective view of the treatment plan that allows providers and carers to better understand patient survivorship at different points in the life journey [50, 51]. **R34 GPP** However, because QoL scores are imperfect and can be affected by numerous factors such as pain and itch, infection, and other co-morbidities; and wound severity scores are often unhelpful in differentiating between mild, moderate, or severe wounds [52], the panel suggests the use of a multifactorial approach to assess the balance between QoL and survivorship in EB.

### Co-morbidities associated with EB impacting survivorship

Kidney failure, dilated cardiomyopathy, and SCC are only a few of the common co-morbidities experienced by people living with EB that impact overall disease state and survivorship [52–54]. **R35** ↑↑ Early diagnosis and treatment of these comorbidities increases their manageability, slows their progress, and may prolong survivorship [54].

### B: Mental health, quality of life, and life satisfaction

Just as management of physical health is important, so too are mental health, quality of life, and overall life satisfaction that takes into consideration culture, religion, and other belief systems. We seek to identify recommendations and interventions to improve these aspects of the life of a person living with EB and their carers. See Table 3 for a summary of the recommendations in mental health, quality of life, and life satisfaction.

**Table 3:** Recommendations Table: Mental health, quality of life, and life satisfaction

No.	Recommendation	Strength of recommendation	Key references*
*General*			
R1	See R1 in Table 2 above	↑↑	[8, 9, 11–19] → [9, 10]
R36	Both the symptoms and treatments associated with EB require equal consideration when providing care necessitating that medical providers consider the whole person living with EB that is beyond the visible lesions	↑↑	→ [10] [12, 24, 55–57]
R39	People living with EB must have the same opportunities to feel that they are a useful and contributing member of society as those individuals unaffected by EB by allowing them to grow emotionally, spiritually, and intellectually rather than holding them back because of any actual or perceived physical challenges that they may have	GPP	
R41	Medical providers should help reduce the costs to the person living with EB and his/her family through judicious use of materials	↑↑	[62]
R44	Increasing public awareness of the biopsychosocial challenges caused by EB as well as addressing fears of contagion or abuse is necessary	↑↑	[13, 73]
R49	Health care providers should provide multi-faceted health care education to benefit patients and carers to encourage active engagement in the process of one’s own medical decision making throughout the lifespan	↑↑	[9, 13, 14, 16, 20, 23, 34, 43, 49]
R50	Established models for practice should be utilized	↑↑	[13, 16, 20, 34, 43, 49]
R51	Interventions provided by healthcare teams should target the development of personal support systems for people living with EB	↑↑	[15, 47, 49, 59, 87]
*Patient, family, and carers mental health*			
R37	The mental health of parents, carers, and providers should be addressed	↑↑	→ [10] [21, 47, 56–60]
R38	Focus should be placed on the emotional needs of the family and of the parent/child relationship	GPP	[59–61]
R40	Children living with EB should be encouraged to develop psychosocially and live their lives despite having EB while either accepting or distancing themselves from the disease	↑↑	→ [10] [60]
R43	Formal and informal support and advocacy groups and solutions-focused psychotherapy, while being mindful of trauma, can help with coping related to EB, stigma, bullying loss, challenging decision-making, and bereavement while encouraging fuller participation in society, a sense of self-realization, having a fulfilling social life, and good peer support	↑↑	→ [10] [48, 56–58, 60, 65, 72, 73]
R45	Family members need long-term support while helping to improve coping with the emotional burden associated with providing care and with bringing in respite	↑↑	→ [10]
R46	Psychological support of the person living with EB, their families, and carers is very important after a diagnosis of SCC, in end-of-life decision-making, or following bereavement	↑↑	→ [9]
*Provider mental health*			
R34	See R34 in Table 2 above	↑↑	[10, 20, 47, 48]
*Quality of life*			
R42	QoL and other psychological assessment/screening tools should be considered to help evaluate the impact of EB	↑↑	[13, 15, 16, 40, 50, 51, 55, 56, 59, 63–71]
R47	Involvement of an IDT (where each member has expertise in treating EB) that provides a wholistic treatment approach aids in the improvement of overall QoL, especially when the person living with EB decides their own therapeutic goals	↑↑	[2, 12, 13, 23, 26, 29, 32–34, 40, 53, 59, 61, 63, 74–84, 87]
R48	To improve QoL, symptoms should be managed through medical or surgical interventions	↑↑	→ [10, 26] [23, 24, 27, 34, 35, 40, 84, 85]

Recommendations are based on the results of the literature review. In addition, other recommendations relating to palliative care were added during the process of guideline development from expert consensus, and the experience of the guideline development group. To provide easier access to information, the recommendations in this table are grouped in accordance with the clinical questions and are arranged by clinical subheadings. If a recommendation fit within multiple subheadings or within multiple clinical questions, it was listed in each. Recommendations were not listed in order of strength or importance. Recommendation strength was strongly influenced by expert panel decision-making, which accounts for observable gaps between evidence levels and recommendation strength. The evidence level is very low for all recommendations. For the strength of recommendation ratings see Table 1. EB, epidermolysis bullosa; GPP, good practice point; G-tube, gastrostomy feeding tube; JEB, junctional epidermolysis bullosa; IDT, interdisciplinary team; QoL, quality-of-life; RDEB, recessive dystrophic epidermolysis bullosa; SCC, squamous cell carcinoma. *Right-pointing arrows (→) denote a guideline document

### B.1 Mental Health/Well-being

### The importance of emotional well-being

**R36** ↑↑ Because the symptoms and treatments associated with EB (e.g., pain, itch, adverse effects of treatment, and social isolation) can negatively impact the physical and emotional health of the person living with it, both require equal consideration when providing care [10, 12, 24, 55, 56] necessitating that medical providers consider the whole person living with EB that is beyond the visible lesions [57]. **R37** ↑↑ Similarly, addressing the mental health of parents, carers, and providers is also important [10, 21, 47, 56–60]. **R38 GPP** Just as with families unaffected by EB, the mental health of the parents and of the child living with EB impact each other [59–61] and family functioning is improved when focus is placed on the emotional needs of the family and of the parent/child relationship [60]. **R39 GPP** The panel believes that people living with EB must have the same opportunities to feel that they are a useful and contributing member of society as those individuals unaffected by EB by allowing them to grow emotionally, spiritually, and intellectually rather than holding them back because of any actual or perceived physical challenges that they may have. **R40** ↑↑ Similarly, children living with EB should be encouraged to develop psychosocially and live their lives despite having EB while either accepting or distancing themselves from the disease [10, 60].

**R41** ↑↑ While not all costs of EB are financial, medical providers should help reduce the costs to the person living with EB and his/her family through judicious use of materials [62].

**R42** ↑↑ Assessment measures of QoL should be utilized to assist in monitoring for present and evolving levels of life satisfaction and identify those in need of additional psychosocial support [50, 51, 55, 56, 63–66]. Additional measures that differentiate between individual versus assumed independence, participation, or other values are also needed by the EB community to support individualized care and ensure inclusive healthcare experiences [13, 15, 16, 40, 51, 55, 59, 63, 67–71].

### The importance of support and public awareness

**R43** ↑↑ Formal and informal support and advocacy groups and solutions-focused psychotherapy, while being mindful of trauma, can help people living with EB, their families, and their carers learn to better cope not only with EB itself, but also with stigma, bullying loss, challenging decision-making, and bereavement while also encouraging fuller participation in society, a sense of self-realization, a fulfilling social life, and good peer support. [10, 48, 56–58, 60, 65, 72, 73] **R44** ↑↑ Increasing public awareness of the biopsychosocial challenges caused by EB as well as addressing fears of contagion or abuse is necessary to improve the general public’s view of people living with EB [13, 73].

**R45** ↑↑ Family members need long-term support while helping to improve coping with the emotional burden associated with providing care and with bringing in respite [10]. **R46** ↑↑ Perhaps at no other time, however, is psychological support of the person living with EB, their families, and carers more important than after a diagnosis of squamous cell carcinoma (SCC), in end-of-life decision-making, or following bereavement [9].

### B.2 Quality of life for people living with EB and their carers

### Interdisciplinary team

**R47** ↑↑ While it is well documented that EB influences the QoL of both the person living with EB and their family [61, 63, 74], involvement of an IDT aids in the improvement of overall QoL [75]. As the clinical severity of EB increases, the need for a holistic treatment approach becomes increasingly important [12, 59, 76]. Specifically, specialist services and specialist interventions working as part of the IDT team, in tandem with the person living with EB who is deciding their own therapeutic goals [75, 77], have been shown to improve QoL in multiple areas of functioning [2, 23, 26, 29, 32–34, 40, 53, 78–84]. It is important that all clinicians providing treatment for people living with EB have expertise with the condition [13] as they will be more likely to understand specific concerns of patients living with EB, such as negative societal perceptions due to the presence of skin wounds and their effects on social interaction and employment, body self-image issues, symptom management, and practical issues, such as choosing appropriate clothing.

### Symptom management and support

**R48** ↑↑ Symptom management by the use of medical or surgical interventions such as placement of artificial feeding devices (e.g., gastrostomy feeding tube) [23, 34, 40, 84]; endoscopic management of oesophageal strictures [35]; pain [2, 10, 26]; itch and its consequences (e.g., difficulty sleeping, scratching, loss of concentration, disturbed routine activities, bad mood), especially at bedtime [2, 24, 27, 85, 86]; and periods of inactivity [24, 85] can significantly improve QoL in people living with EB.

### B.3 Life satisfaction

**R49** ↑↑ Health care providers should provide multi-faceted health care education to benefit patients and carers to encourage active engagement in the process of one’s own medical decision making throughout the lifespan. This should include, but not be limited to, the topics of nutrition, palliative care services, valued occupational/activity participation, and end-of-life care approaches [9, 13, 14, 16, 20, 23, 34, 43, 49]. **R50** ↑↑ Utilization of established models for practice is recommended when addressing these needs [13, 16, 20, 34, 43, 49], an example of which can be found in the 2012 article by Budych, Helms, and Schultz [16].

**R51** ↑↑ Interventions provided by healthcare teams should target the development of personal support systems for people living with EB. This may include areas related to family/personal values, community and home contexts, psychosocial function, perceptions of control, healthcare experiences, and other psychosocial and physical aspects of life experience/participation [15, 47, 49, 59, 87]. For a thorough examination of the psychosocial care recommendations for people living with EB, and of supporting sexuality for people living with EB the panel directs the reader to the 2019 Psychosocial recommendations for the care of children and adults with epidermolysis bullosa and their family: evidence based guidelines [10] and the 2021 Supporting sexuality for people living with epidermolysis bullosa: clinical practice guidelines [88], respectively.

### C: Grief

Although the evidence meeting criteria for inclusion within the systematic review included only five articles that addressed grief, the panel found considerable consensus among healthcare providers and the EB community that identifying individuals who are grieving because of EB and helping them identify effective coping mechanisms is important for those who live with or manage EB. Here, we discuss the best practices to do that. It is important to emphasize that grief is a topic not only reserved for cases of death, but also at any point in life when physical, emotional, spiritual, or cognitive loss is experienced. See Table 4 for a summary of the recommendations in grief.

**Table 4:** Recommendations table: grief

No.	Recommendation	Strength of recommendation	Key references*
R1	See R1 in Table 2 above	↑↑	[8, 9, 11–19] → [9, 10]
R52	Psychosocial support for parents is necessary to address the special kind of trauma unique to parents of a child with EB	↑↑	[59]
R53	Provide a strong system of support for individuals throughout the dying process and beyond	↑↑	→ [9]
R54	Health care providers should offer ongoing bereavement support following a death	↑↑	→ [9]
R55	Allow health care providers to attend funerals of their patients if allowed by the family	GPP	[47]

Recommendations are based on the results of the literature review. In addition, other recommendations relating to palliative care were added during the process of guideline development from expert consensus, and the experience of the guideline development group. The recommendations in this table are not arranged according to outcome; rather they appear sequentially and are grouped in accordance with the clinical questions. If a recommendation fit within multiple subheadings or within multiple clinical questions, it was listed in each. Recommendation strength was strongly influenced by expert panel decision-making, which accounts for observable gaps between evidence levels and recommendation strength. The evidence level is very low for all recommendations. For the strength of recommendation ratings see Table 1. EB, epidermolysis bullosa; GPP, good practice point; G-tube, gastrostomy feeding tube; JEB, junctional epidermolysis bullosa; IDT, interdisciplinary team; QoL, quality-of-life; RDEB, recessive dystrophic epidermolysis bullosa; SCC, squamous cell carcinoma. *Right-pointing arrows ( →) denote a guideline document

The first time that EB treatment teams are confronted with severe grief often occurs as early as shortly after the birth of a child who is diagnosed with EB. When expecting a child, parents envision their future baby as being healthy. However, several strong emotions, including grief, can occur after realizing the unexpected challenge of having a child born with an obvious and incurable health problem. In this period, grief can be intense and is often related to the loss of, or significant changes to, the parents’ original hopes and dreams. **R52** ↑↑To address this special kind of trauma that is specific to parents of a child with EB, we strongly recommend psychosocial support to help them accept the condition and to move forward in their lives [59].

Grief is not only experienced by parents, but also the person living with EB. Several periods over the lifespan of a person living with EB can cause grief, such as the heightened challenges of daily routines associated with increased disability, dreams of job trainings or partnerships not coming true due to health issues, or new pain negatively affecting quality of life. While there are no guidelines on how to grieve “best”, it is important to understand that everyone grieves differently and over different periods of time [57].

The period shortly before death is one of the most vulnerable times in a person’s life. For the affected individual, their family, and their carers, grief in all its manifestations can be a strong and consuming companion not only during the terminal phase of the illness, but also in the time that follows. **R53** ↑↑ A strong system of support for these individuals throughout the entire dying process and beyond is highly recommended. **R54** ↑↑ Health care providers, especially mental health professionals, who work with people living with EB, should offer ongoing bereavement support following a death, as may be welcomed by the family and carers [9].

Families and carers are not the only ones who can experience grief after the death of a person living with EB. The entire treatment team is also suffering a loss. Over the course of what is often many years of intense treatment, relationships between people living with EB, their families, and their health care providers can become very close. **R55 GPP** If invited or allowed by the family, health care providers should feel free to attend funerals of their patients as this can bring closure and support to everyone involved [47].

## Discussion

Although EB is a potentially life-limiting condition with a multitude of significant complications and yet no cure, all care for the person living with EB must be considered palliative. Treatment by an IDT should begin shortly after birth and, over the course of the lifetime, focus on the reduction of suffering while improving physical and emotional comfort and overall quality of life. The recommendations gathered in this systematic review demonstrate that healthcare providers have many options to achieve those goals.

### Future research

Table 5 identifies recommendations for several areas of future research.

**Table 5:** Areas for future research

1	Develop best care practices for healthcare providers specific to the needs at end-of-life
2	Examine how cultural and ethnic beliefs as well as local, state, provincial, and or national regulations affect the provision and reception of palliative care services within the EB population both globally and during individual visits with the medical provider(s)
3	Develop best care practices for healthcare providers in areas where access to interdisciplinary team and/or medical resources are limited due to things such as geography, economic conditions, and geopolitical tensions
4	Develop best care palliative care practices for healthcare providers that are specific to EB subtype
5	Develop best care palliative care practices for healthcare providers that are age group-specific (e.g., infancy, toddlerhood, childhood, adolescence, young adulthood, adulthood, older adulthood)
6	Evaluate the generalizability of the palliative care literature and, specifically, EoL comfort care literature related to people with other potentially life-limiting conditions such as cardiovascular diseases, cancer, chronic respiratory diseases, diabetes, and others to people living with EB
7	Further exploration of the experience of grief in people living with EB, as well as their families, carers, and healthcare providers and should pay special attention to examining EB-related grief across cultures and ethnicities
8	Develop additional pharmacologic treatments and nonpharmacologic skills to reduce pain and itch without increasing negative side-effects
9	Examine the potential satisfaction differences between those living with EB, their caregivers, and their treatment team(s)
10	Develop surveys that a) are EB-specific, wider-reaching, consider mental health conditions, and that measure QoL in small children to better understand the needs of people living with EB and b) differentiate between individual versus assumed independence, participation, or other values that are needed to support individualized care and ensure inclusive healthcare experiences
11	Explore the challenges and process of, and bioethics related to, medical futility and the decisions to provide, alter, or withhold specific aspects of care, especially at end of life, including terminating life-sustaining therapies (e.g., artificial nutrition) as well as the use of comfort care, palliative sedation, and the Groningen Protocol/euthanasia, especially in cases of the most severe subtypes of EB such as severe JEB subtype

### Implementation and dissemination of guideline recommendations

DEBRA International aims to ensure that the EB guidelines address the needs of patients internationally. These guidelines will be translated into other languages and patient versions will be made to aid accessibility. It is important for the reader to understand that future studies may require a change in these recommendations just as some situations may warrant a deviation from these recommendations. Additionally, these guidelines are not to be used to replace clinical judgement, but rather should be used to aid its implementation. DEBRA International would value your feedback on the findings to continue to improve CPG quality.

### Updating procedure

This CG should be updated every 3–5 years after publication, or earlier if there is a significant breakthrough in EB palliative care treatment. The panel recommends a literature search to determine whether a full review is warranted at any stage.

## Conclusions

Palliative care for people living with inherited EB begins at diagnosis and spans their lifetime. Despite the lack of a cure, understanding that there are not a just few, but rather many, potential interventions that can improve quality of life and reduce suffering. Because of EB’s rarity and multiple comorbidities as well as the impact on the life of the person living with EB, it is critical that all treatment is interdisciplinary with the person living with EB, and his/her/their family and carers at the treatment team’s center.

### Supplementary Information

**Additional file 1**: EB palliative care PPI survey.

**Additional file 2**: EB palliative care clinical survey.

**Additional file 3**: Stakeholder involvement, peer review, and methodology.

## Data Availability

The datasets used/analysed during this study are available from the corresponding author on reasonable request. A patient/family version of this CG will be available to better disseminate this article and will be available on the DEBRA International website in due course.

## Abbreviations

AGREE II: Appraisal of guidelines for research and evaluation II
CG: Consensus guideline(s)
CPG: Clinical practice guideline(s)
DEB: Dystrophic EB
DDEB: Dominant dystrophic epidermolysis bullosa
DEBRA: Dystrophic epidermolysis bullosa research association
EB: Epidermolysis bullosa
EBS: Epidermolysis bullosa simplex
EoL: End-of-life
GER: Gastroesophageal reflux
GI: Gastrointestinal
GS-JEB: Generalized severe junctional epidermolysis bullosa (previously called Herlitz-type)
IDT: Interdisciplinary team
EBK: Kindler syndrome
NICHD: Eunice Kennedy Schriver National Institute of Child Health and Human Development
NZ: New Zealand
PICOS: Participants, Interventions, Comparisons, Outcomes and Study design
PPI: Patient population involvement (people living with EB or their carers)
QoL: Quality of life
RCSI: Royal College of Surgeons in Ireland
RDEB: Recessive dystrophic epidermolysis bullosa
SCC: Squamous cell carcinoma
UK: United Kingdom
USA: United States of America
WHO: World Health Organization
