# Supplementary material for: Consensus-based guidelines for the provision of palliative and end-of-life care for people living with epidermolysis bullosa
Source: Orphanet J Rare Dis. 2023 Sep 4;18:268. doi: 10.1186/s13023-023-02870-8 (PMC10476410; doi:10.1186/s13023-023-02870-8)
Supplement: Supplementary file 1 — Additional file 1: EB palliative care PPI survey. [file 13023_2023_2870_MOESM1_ESM.pdf]

EB Palliative care: PPI Survey

1. Are you a person living with EB?

Number of responses: 125

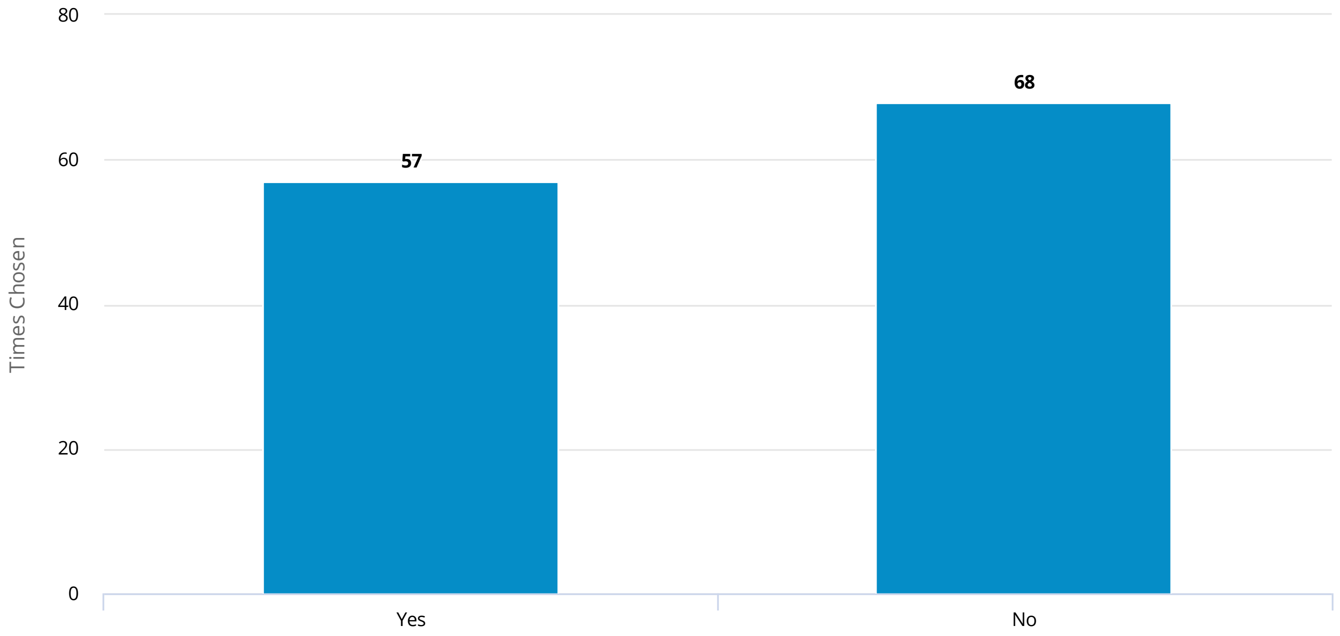

i) Are you

Number of responses: 56

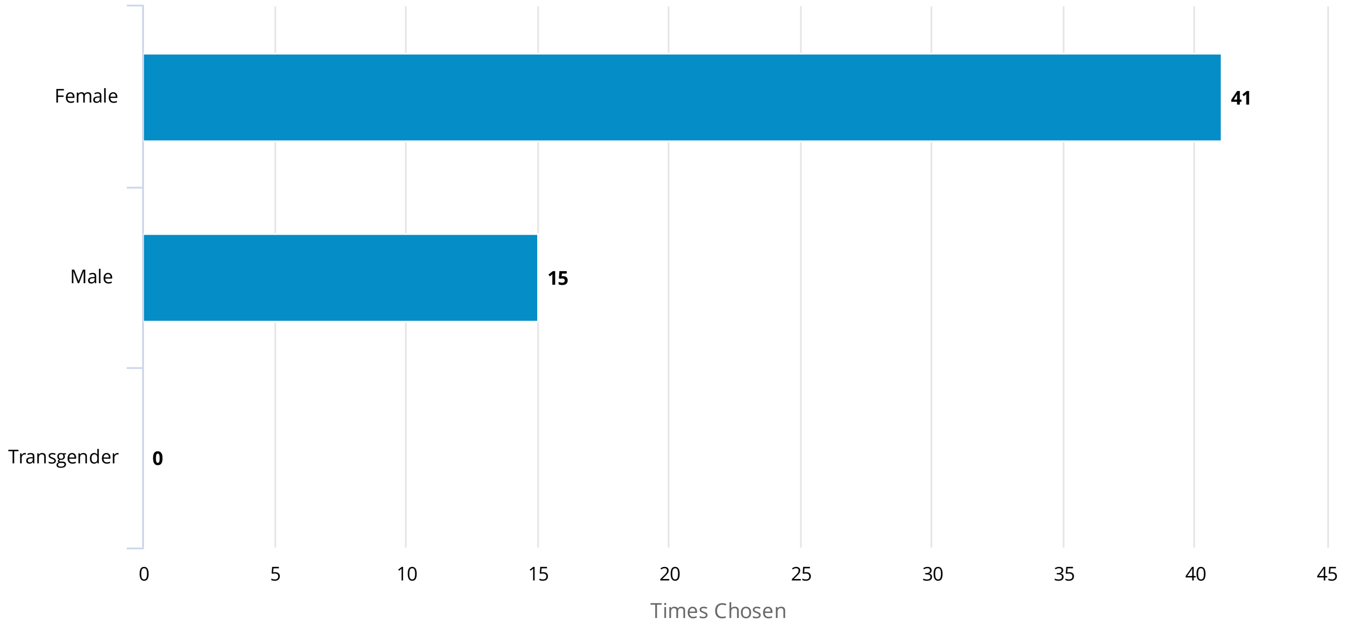

ii) How old are you?

Number of responses: 56

Text answers:

41

29

5

60

31

65

30

36

47

25

85

26

26

55

45

37

26

31

32

42

I am 65 years old

28

48

52 years old

23

64

26

76

81

46

30

21

61

35

25

38

25

72

29

39

35

44
32
34
38
63
38
43
39
39
56
61
36
26
37
20

2. Are you a parent or a caregiver of someone living with EB?

Number of responses: 124

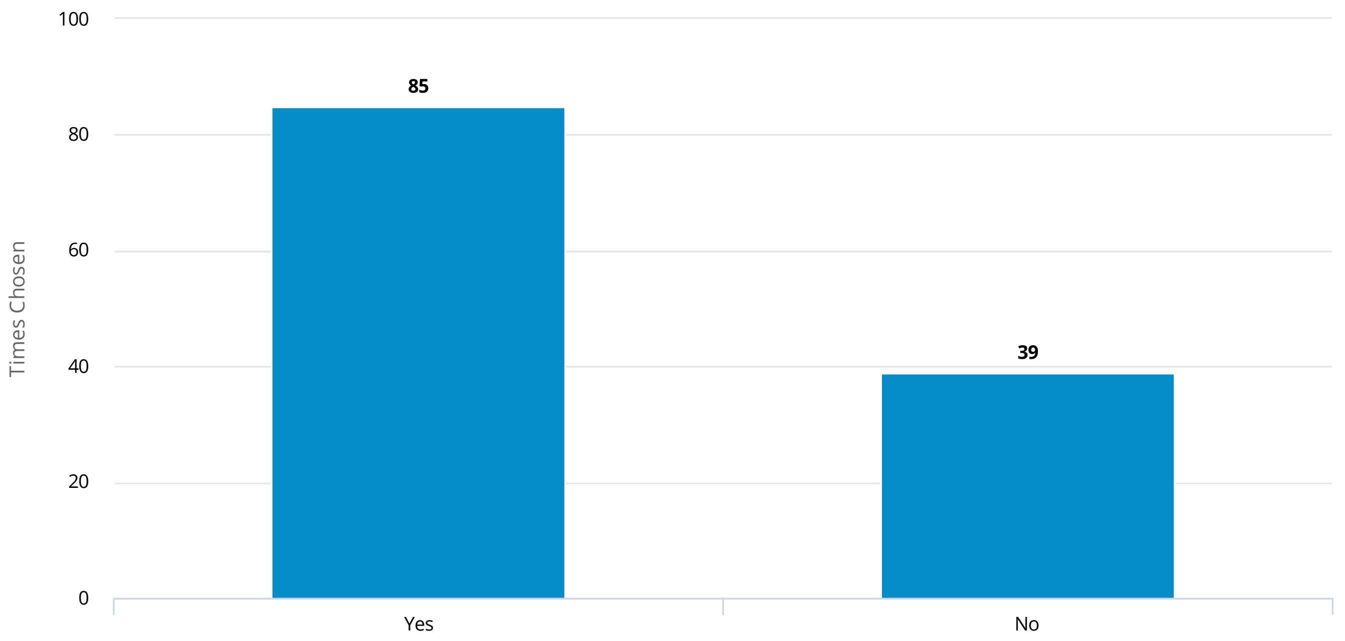

ii) Is the person you care for

Number of responses: 85

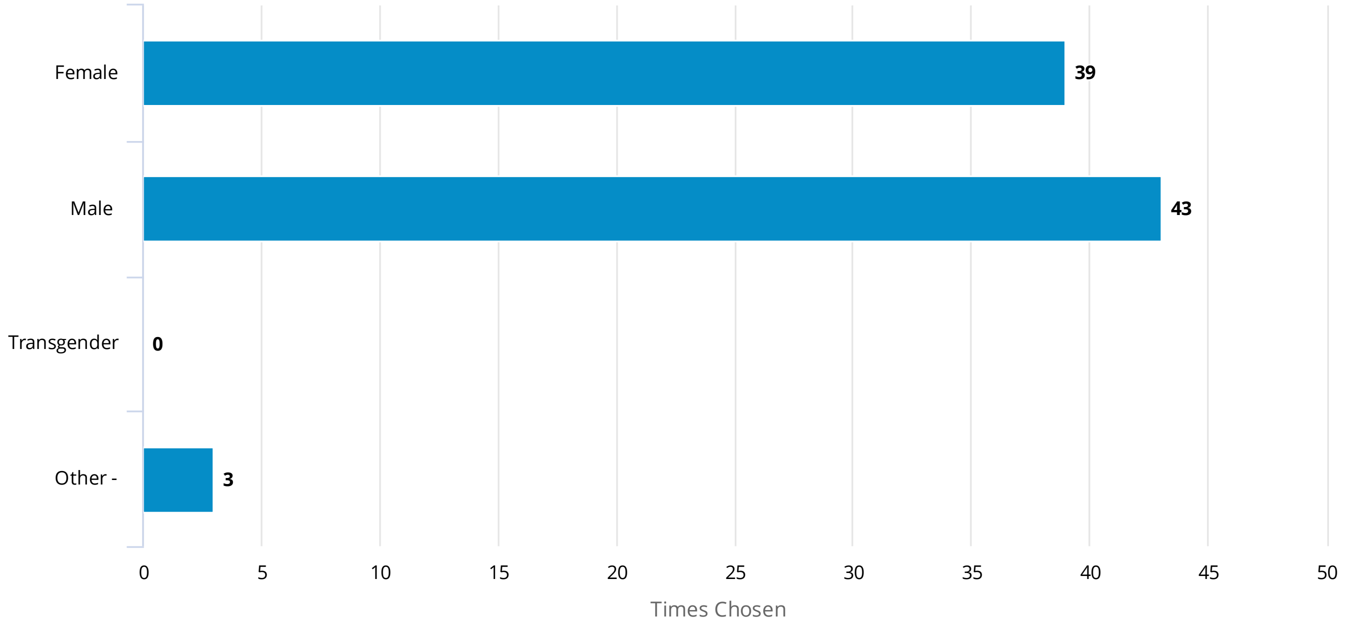

"Other -" text answers:

My son and daughter, male and female
I have 2 boys and 2 girls with RDEB
It was my son

iii) How old are they?

Number of responses: 85

Text answers:

5
3
5
9
2
4
4 years
4
19
20
13
26
6 weeks
9
13
3 Years
87
18
12

12
3.5 years old
34
23
31
5
5
14
3
2
8
6
6
25
10 months
26
5
5
12
6

6
39
67
24
4
6
4
16
21 & 12 y
8 months
14
14
7 months
20
9
10 and 4
10
9
11
12
7 months

32

Deceased at 11yr old. Would be 21 today

11

3

12

46

18

Almost 2 years old

19

He was 20 when he passed

24

Avery has 28 months when she passed away

7,8,9,17

6

10

8

35

3

3.5

8

14

5 months

2 yrs

She passed away in 2017 but was 17 years old

5

3. Based on the image below, please select the area in which you live.

Number of responses: 121

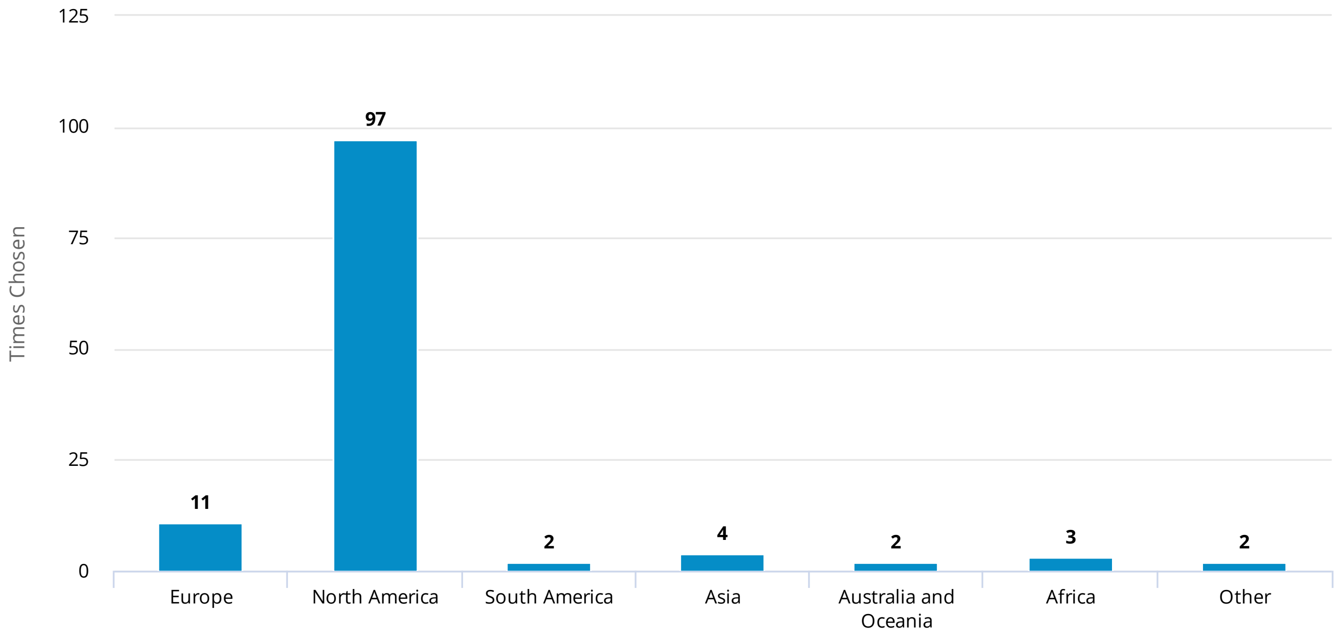

a) Can you tell us what city, state and country you live in?

Number of responses: 121

Text answers:

Surprise, AZ US

Huntington Indiana USA

Eugendorf, Salzburg, Austria

Bridgeport. Ct

Österreich
South Padre Island, Texas
Pakistan
San Antonio TX USA
San Antonio TX USA
Pakistan, karachi
Alabama
Columbus Ohio USA
Appleton WI
Cape Town
Utica NY
Lee's Summit, MO 64063, USA
Orange California
Champlin, MN
Lahore, Punjab, Pakistan
Gulf Shores, Alabama
Moncton, NB, Canada
California
Kansas City, MO USA
Minneapolis, Minnesota. USA
Centreville, VA USA

Bethlehem, Pennsylvania USA
New Orleans, LA. USA
Cotia, São Paulo , Brazil
San Diego, CA
Huntsville, Alabama
mississippi
Lancaster, CA
Nobleboro , Maine , United States
Crosby, tx
Chapel Hill, NC USA
New York, New York
Hagerman Idaho United States
Coventry, CT USA
orlando, FL USA
Florence, South Carolina
Indianapolis, Indiana USA
Boonville, IN
Muretta, CA USA
I live in BRISTISH COLUMBIA, CANADA
Atlanta, Georgia

Redding California

hamilton, ohio

College Station, TX USA

Mastic   NY

Boston, MA

UK. Greater Manchester

Crosby, TX USA

Gibson Iowa USA

Snellville, Georgia

Greensboro, NC, USA

Queen Creek Az United States

Port Jefferson Station, New York 11776

Harrington De USA

Erie, PA United States

Denham Springs LA USA

Mission,Texas uSA

Floyds Knobs, IN USA

Austin, Texas, USA

Indianapolis Indiana USA

Noblesville IN

Melbourne Florida

Rochester, PA USA
Misurata Libya
Anaheim, California
Sheridan, Wyoming USA
Columbus OH
CHICAGO, IL, USA
New Jersey USA
Augusta, GA USA
San Antonio, Texas, USA
Houston, TX USA
Indiana, USA
Chester Springs, Pa.
OKLAHOMA city
Lithia, Florida US
Ontario, Canada
Minneapolis, Minnesota, USA
NYC, NY
American Fork, UT USA
Ocala ,Florida
Chicago, IL USA

Erie, Colorado

JOHOR MALAYSIA

Sayreville, New Jersey USA

Pittsburg, California, USA

Mannheim, Germany

Hamilton, Alabama, USA

South San Francisco, CA, USA

Ohio

Ireland

Portugal

Cleveland Ohio United States

Columbia, KY USA

Scotland

Toledo, Ohio - United States

Radnor, Ohio USA

Bristol Connecticut. United states of America

Michigan

N.Y. N.Y. USA

Gales Ferry Ct US

Bountiful, Utah 84010

Lakewood, CA USA

Phoenix, AZ, USA

Zagreb,Croatia

Casa Grande, AZ, USA

Tucson, Az

Gilbert, AZ, USA

fbdb

New Delhi India

Delhi, India

Melbourne, Victoria

Brasilia, FEderal District, Brazil

Cape Town, Western Cape, South Africa

Zagreb, Croatia

Lyon, France

Caloundra QLD Australia

4. What is the highest level of education you have received?

Number of responses: 114

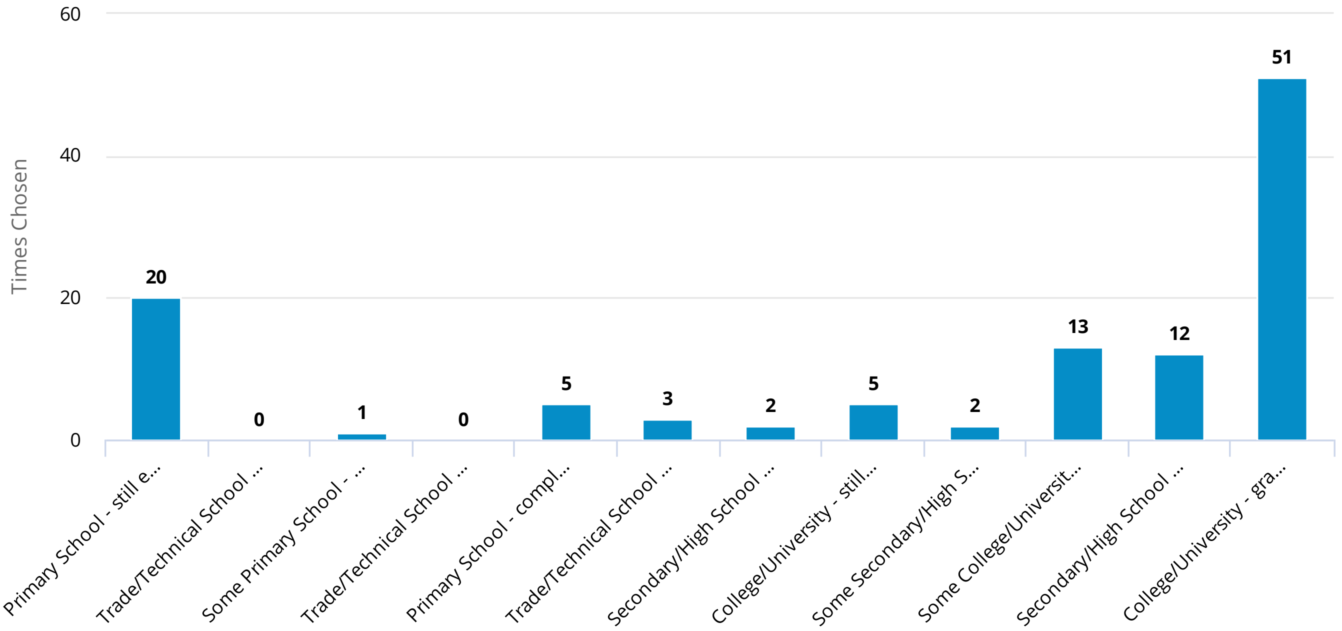

5. Who lives in your home with you?

Number of responses: 118

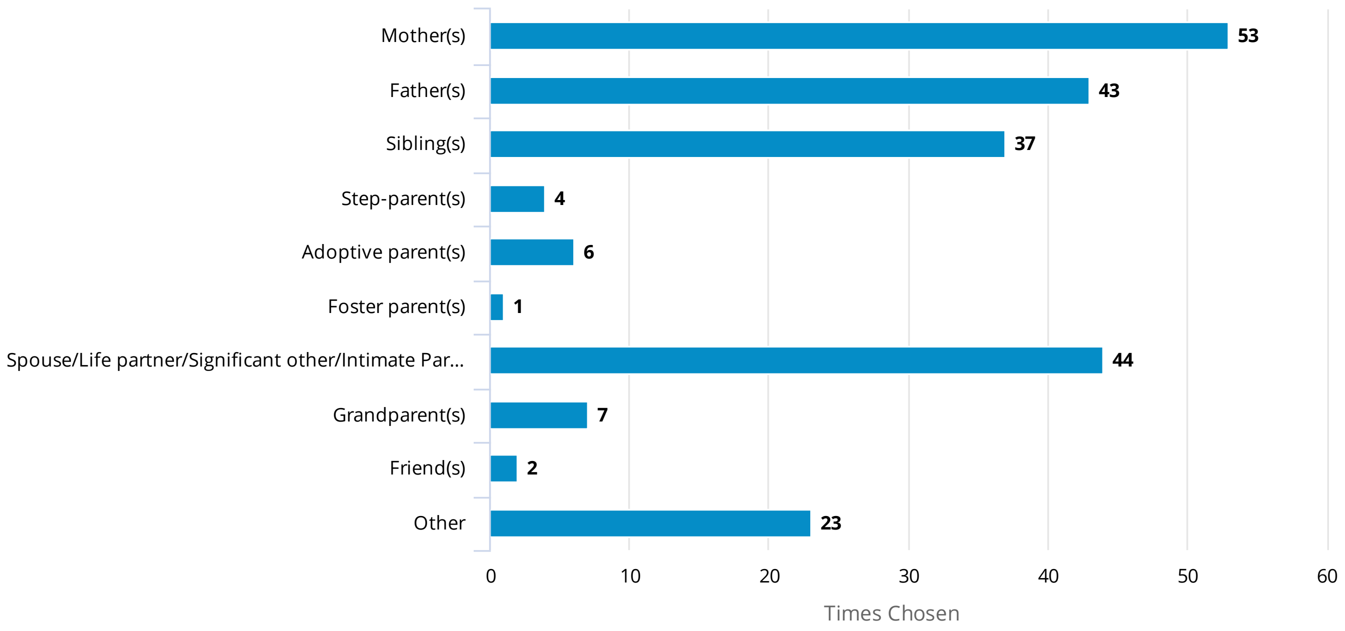

"Other" text answers:

Pets
Kinder
Child, grandchild
Wife ,childrens ,father & mother
His son and my son
My children
Children
my kids
Brother
3 kids
Daughter
Kids, grandkids

0

Alone

child

Me and my children

Children

Son

Child

No-one

Older Son

Children

I live by myself

6. How many people in your household have been diagnosed with EB?

Number of responses: 120

Text answers:

1

1

1

2

1

3

1

1

1

1

01

1

2

2

1

0

1

Just me

1

7

Just me

1

1

1

1

3

1

1

1

2

1

1

one , ME

2

1

1

1

3

Just me

1

1

1

ME

1

3

1

2

Only me

1

1

1

1

2

2

1

1

1

0

2

1

Only myself

1

1

1

1

1

2

1

2

1

1

1

1

Just me

3

1

myself only

3

1

One

1

2

1

1

None but me

1

1

Self and oldest brother growing up. I live on my own.

1

0

1

1

1

1

2

Just me

2

1

1

2

1

1

1

1

4

1

1

Just 1

1

1

```
1
```

```
\bfs\b
```

```
1
```

```
1
```

```
1
```

```
1
```

```
1
```

```
0
```

```
1
```

```
1
```

7. What type of EB do you have?

Number of responses: 124

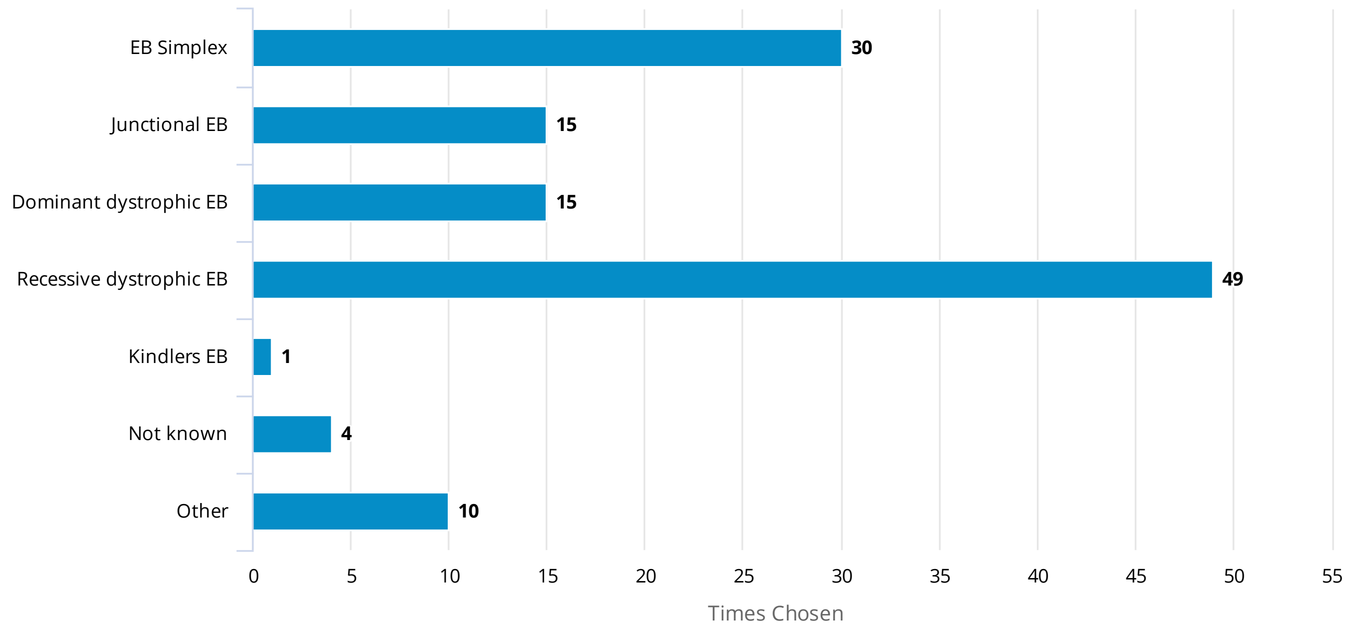

"Other" text answers:

Dystrophic
Epidermolysis bullosa pruriginosa
Still waiting for gene tests. Probably DEB
Dystrophic EB - Inversa
Still waiting on genetic
Dystrophic
Weber-Cockayne
Other
Dystrophic, don't know which type
I don't have it

8. Do you follow a religious or have a cultural affiliation ?

Number of responses: 116

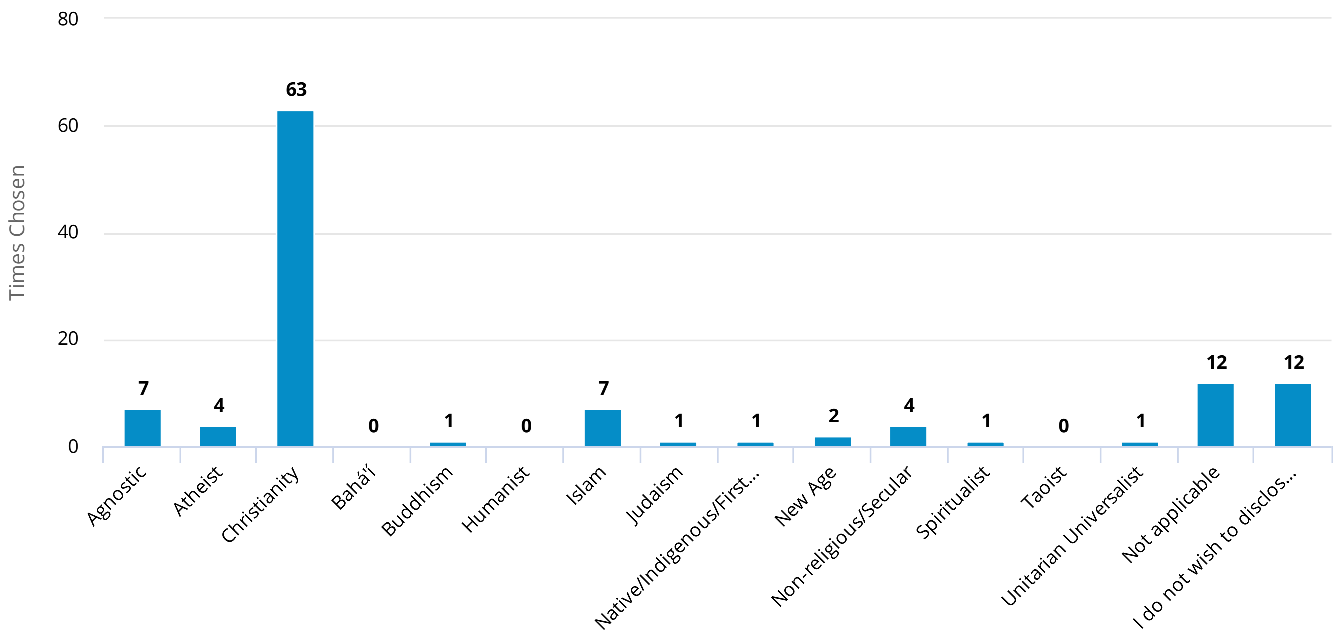

a) Can you tell us more about the level of affiliation with religious/cultural affiliation?

Number of responses: 90

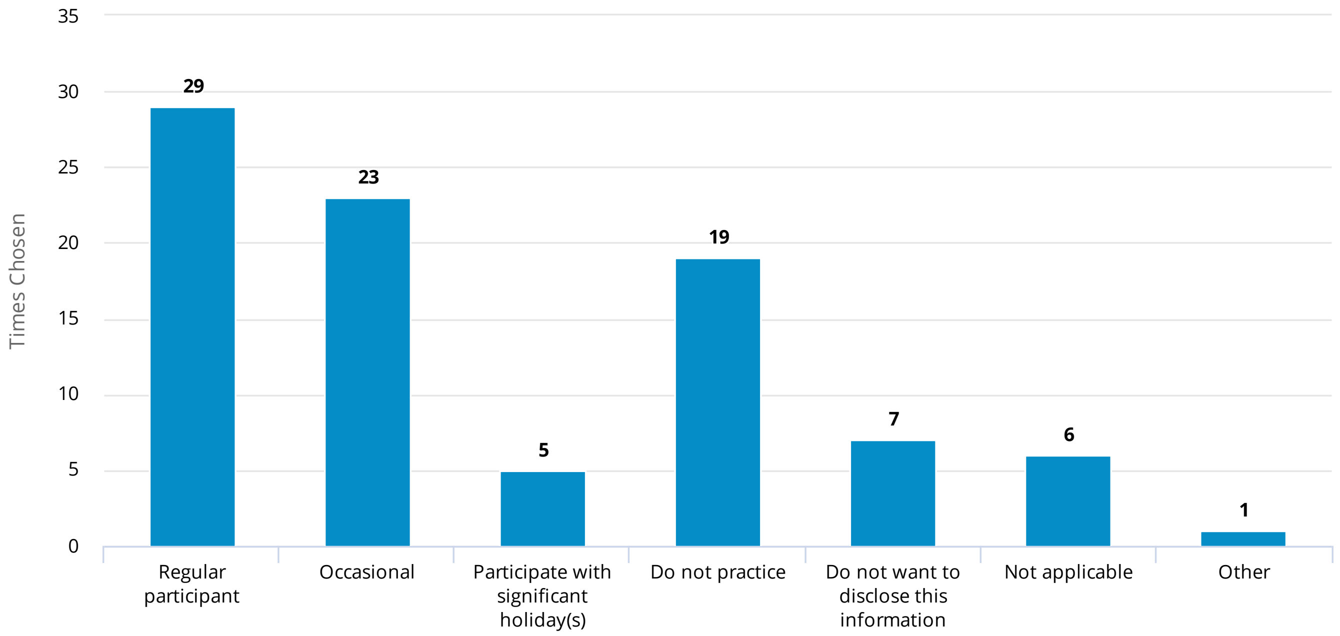

9. Are you currently a member of the Palliative Care CPG Panel?

Number of responses: 115

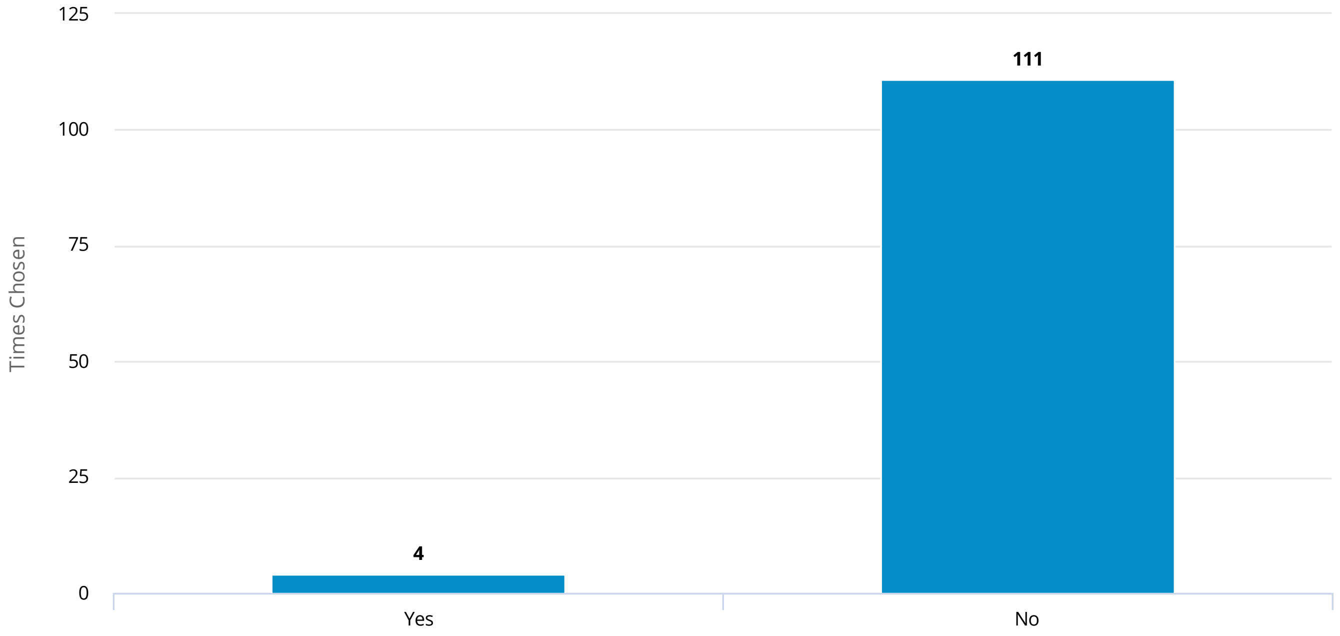

1. Select all healthcare or support professionals that you utilize.

Number of responses: 70

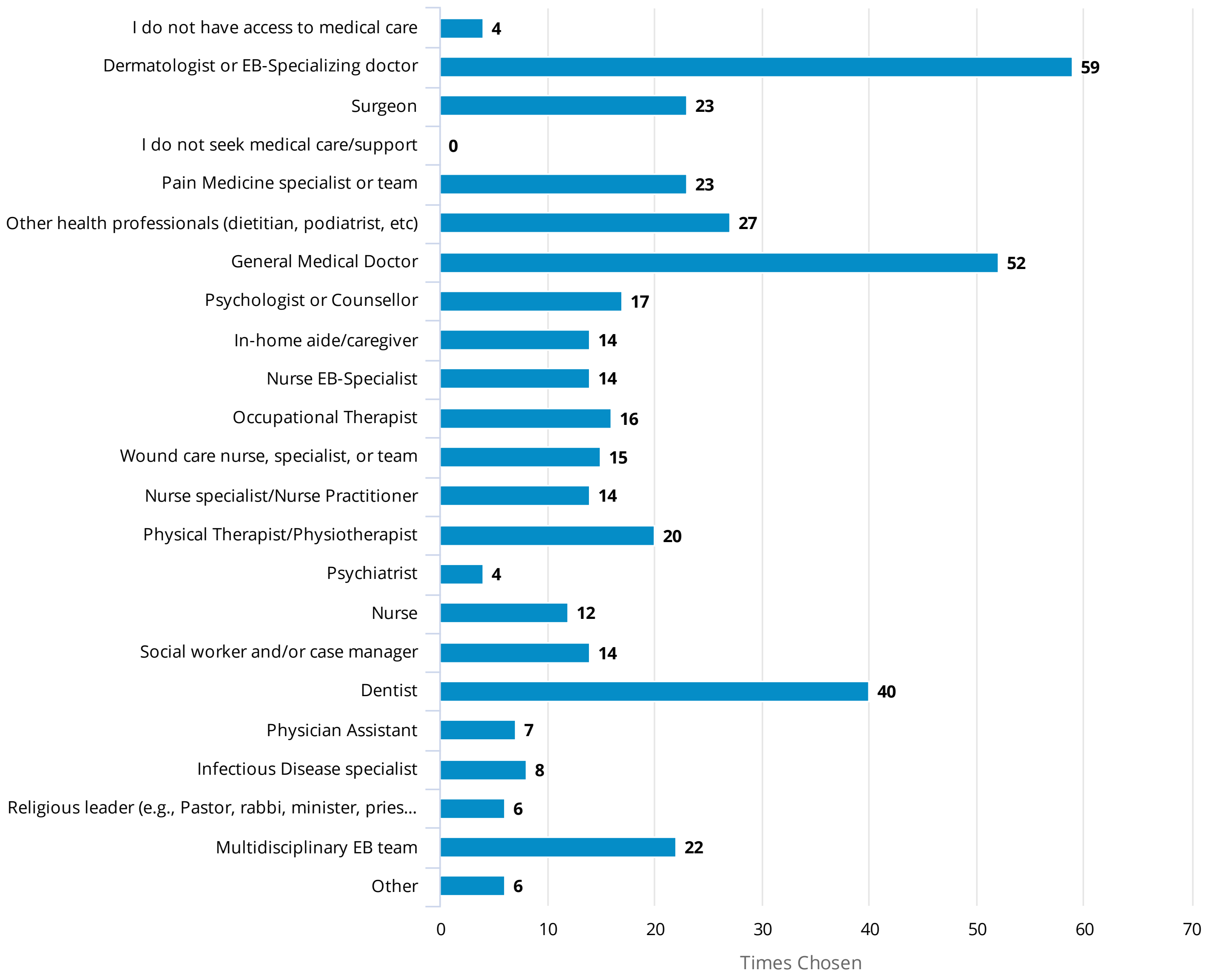

"Other" text answers:

Oncology/BMT
speech therapist, palliative care team
Periodontice
GI doc, endocrinologist
Nurse and CNA for bath and wound care
Other: Hematologist and orthopedic surgeon

2. Select all healthcare or support professionals that you believe should be part of your Palliative Care Team.

Number of responses: 71

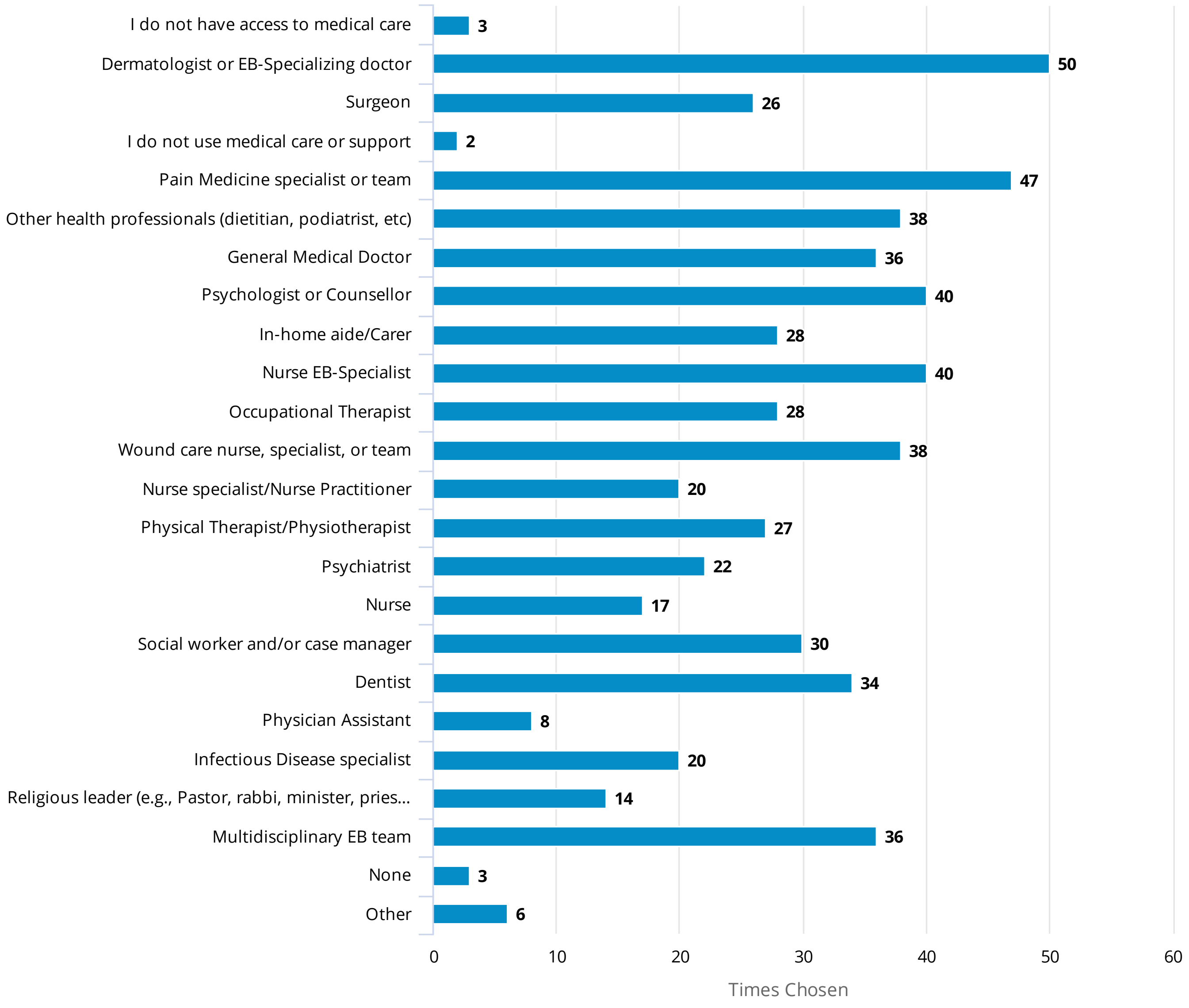

"Other" text answers:

Immunology, rheumatology,
infectious diseases
I have everyone I should and have access to additional team members as my health needs evolve
Gastroenterologist
All of the above AND Palliative care team
reimbursed psychological support for parents

3. What support systems do you utilize to help you to cope with EB?

Number of responses: 70

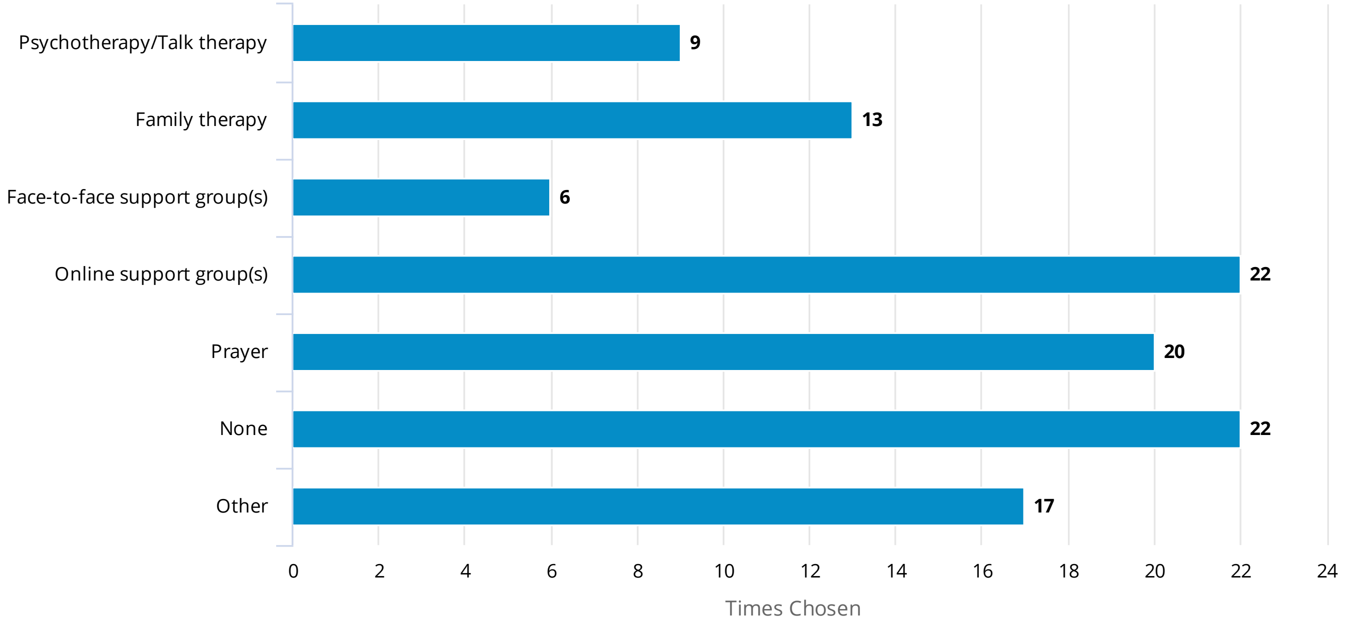

"Other" text answers:

Xbox friends
Camp and other activities with other kids with EB
Family & Friends
play therapy (as a part of palliative care team)
Speaking with parents (who happen to be mental health professional)
Meditation and smudging
spouse, Medplex wound care bandages EB Dermotologist
Other Eb parents
Other
iPad, movies, video games
Family
DEBRA USA Patient Care Conference

Dbt

Friends

She's 3.5

Family Support within the primary family group

Holistic therapy

4. If you have participated in a support group, what kind was it?

Number of responses: 23

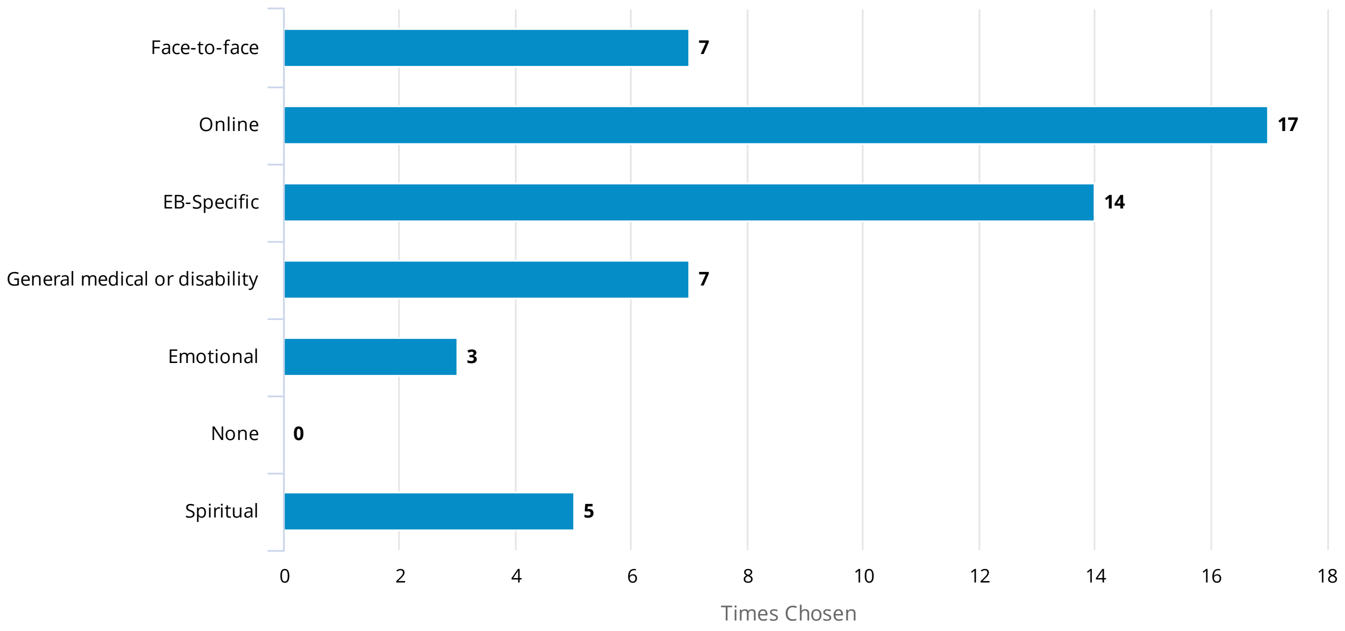

a) If you used a support group, was it helpful?

Number of responses: 70

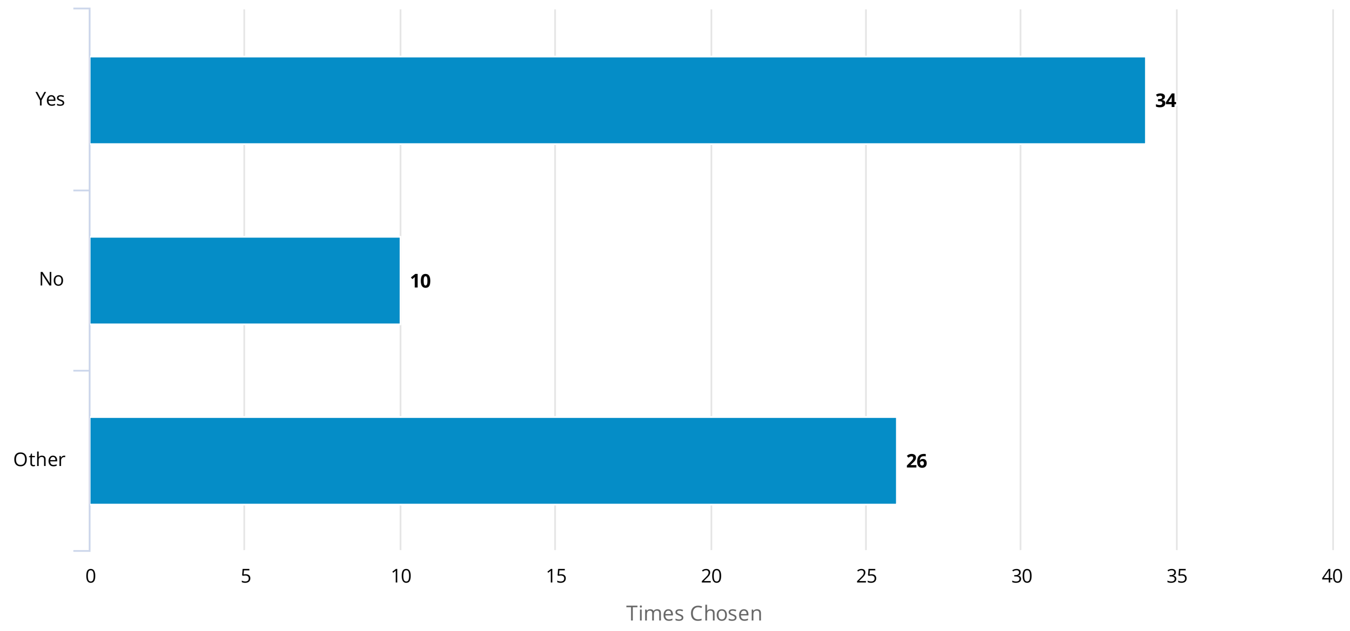

"Other" text answers:

Not applicable
I haven’t
I helped young parents with EB children.
At times
na
N/A
Other
na
Na
somewhat
Didnt
NA

I did not

N/A

Sometimes

Other

Have not used

Other

N/A

Have not used one, so can´t tell

Na

Mainly dependent upon the understanding of the rarity of the disease and the true facets of challenges we face - no just what you see.

Never been

Na

Other

NA

Please tell us more:

Number of responses: 24

Text answers:

Get useful tips and ideas how handle care thins better, exchange news, excange expiriences, recommendations, support, talk about not EB-related topics if possible too;

Young parents with EB children had no idea what they were in for.

It is highly helpful to seek advice from others experiencing same issues and can cut some of the trial and error out of things to try. Others recommendations have greatly impacted my life.

No

Prescriptions for the first aid are helpful and i get moral support by connecting with different people living with EB in Pakistan

Living in New Brunswick I have a difficult time seeing even my family doctor, it is very scary and causes me stress.

the Epidermolysis Bullosa Private Lounge on Facebook to communicate with care givers and those living with EB for advice; I've learned more from them than any of our doctors or other EB organizations.

just playing with others

It would be nice to speak to others who have EB

do not go to support groups

online groups help me feel like I'm not alone but I wish that there was a face to face group in the Houston area.

I am a very private person and I do not discuss my condition with anyone except my doctors and my immediate family.

Nice to share day to day living experiences with others

My grandfather, two uncles, father, cousin, & my children have EB. We have used family for knowledge and care

Tips and tricks of what people have tried and worked. Peoples thoughts on wound care products to try.

Haven't used one. Would be open to it.

DBT— Dialectical behavior therapy

Helps validate feelings and fears and hopes.

Good tips on care management on the EB Facebook groups

She's 3.5, but we have introduced her to other children with EB and shown her photos of other chikdren and adults. She's very interested.

rtjharjr

People share their observations

Support groups help us feel we're not alone.

Support groups in general are always helpful for me for 3 reasons a) friendly medical advice b) emotional support and c) being reminded that there are others out there - I can often feel isolated with EB in South Africa

5. Whom do you seek when you are in need of emotional support?

Number of responses: 71

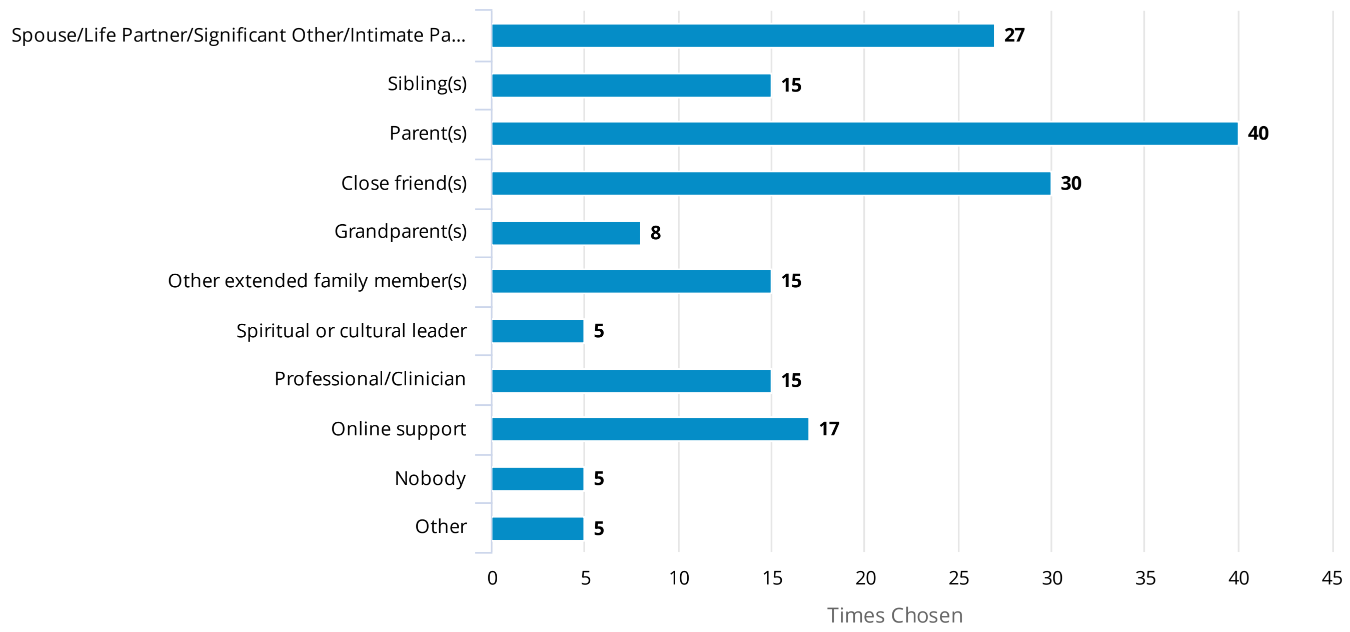

"Other" text answers:

Jesus

My mother and father are deceased but were wonderfully supportive when I was growing up.e

Service dog

Sports

brother, holistic therapist

6. Have you ever heard about Palliative Care?

Number of responses: 71

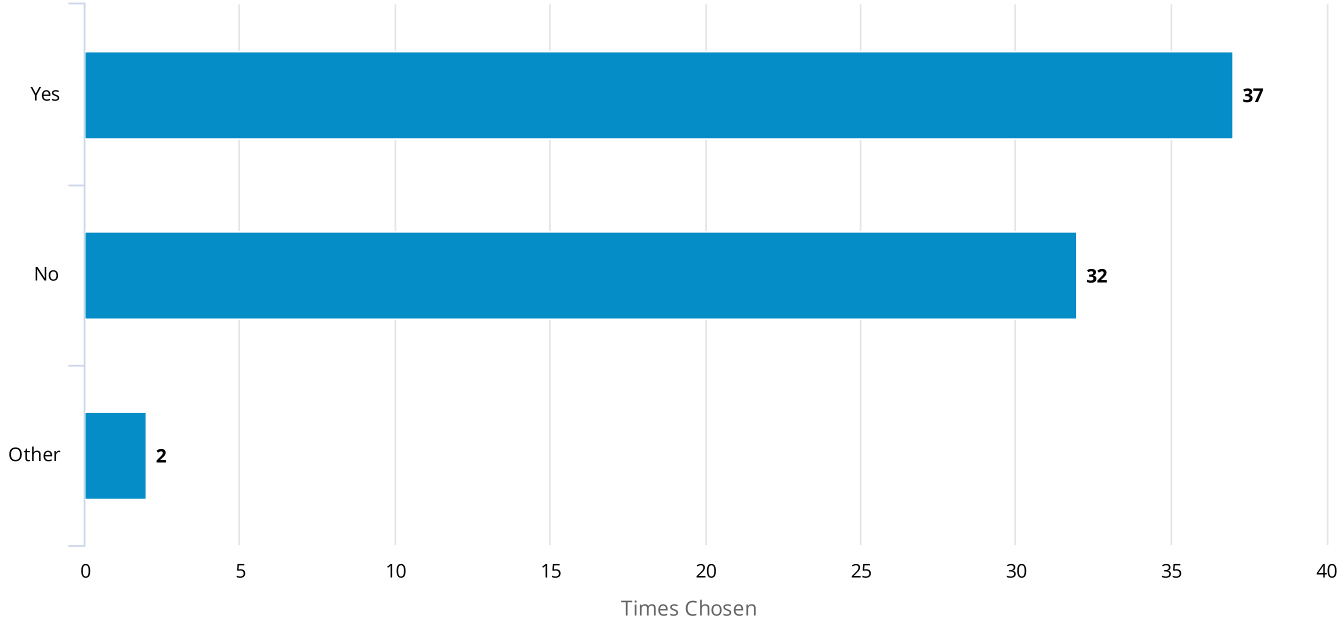

"Other" text answers:

Answering this from perspective of my 5 year old daughters with EB

My pain management Doctor is a palliative care dr.

7. What educational resource(s) have you found or are you using regarding Palliative Care?

Number of responses: 70

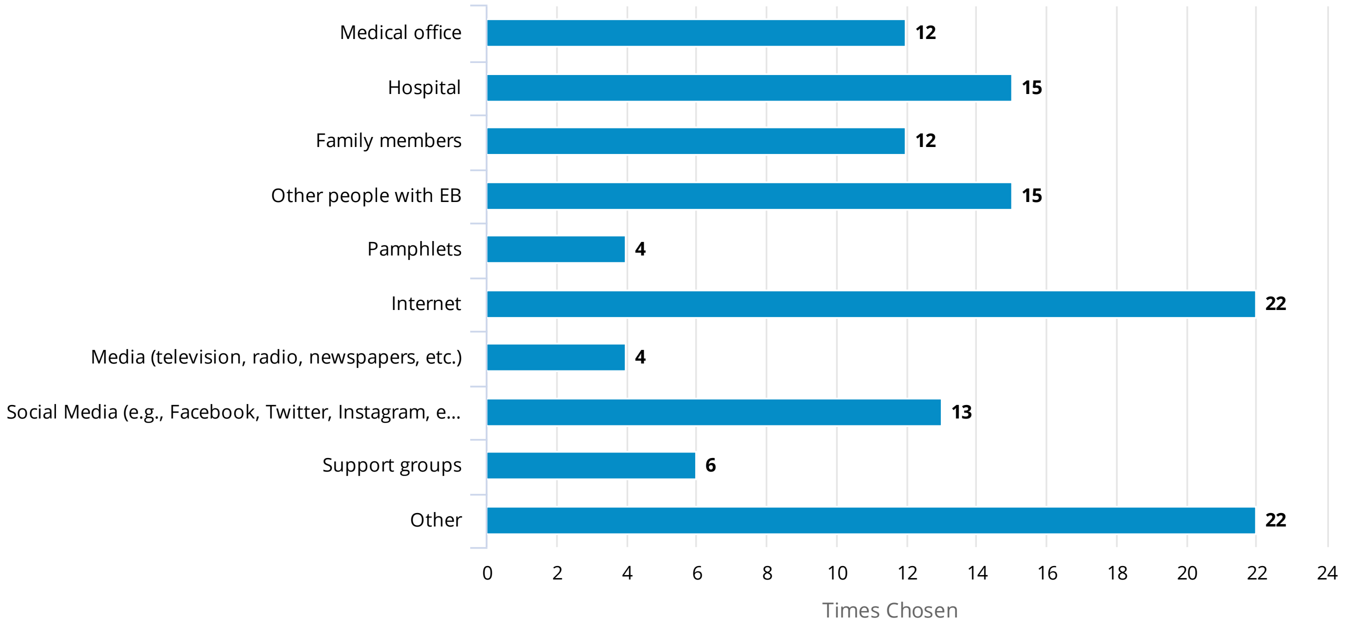

"Other" text answers:

Never knew what it was
I don’t know what it is
None. Did not know it existed.
Nothing
None
haven't looked into it
TransitionsKids (Raleigh, NC) palliative care team, comes to our home
NA
Na
DEBRA
NA
None

None

Do not know

Work- I work in the health care insurance industry

Dr Amy Paller

I worked as a Hospice Nurse.

I’m aware of it professionally

Na

Other

NO one

Palliative care team at royal Children's hospital

8. Palliative Care is for you...

Number of responses: 70

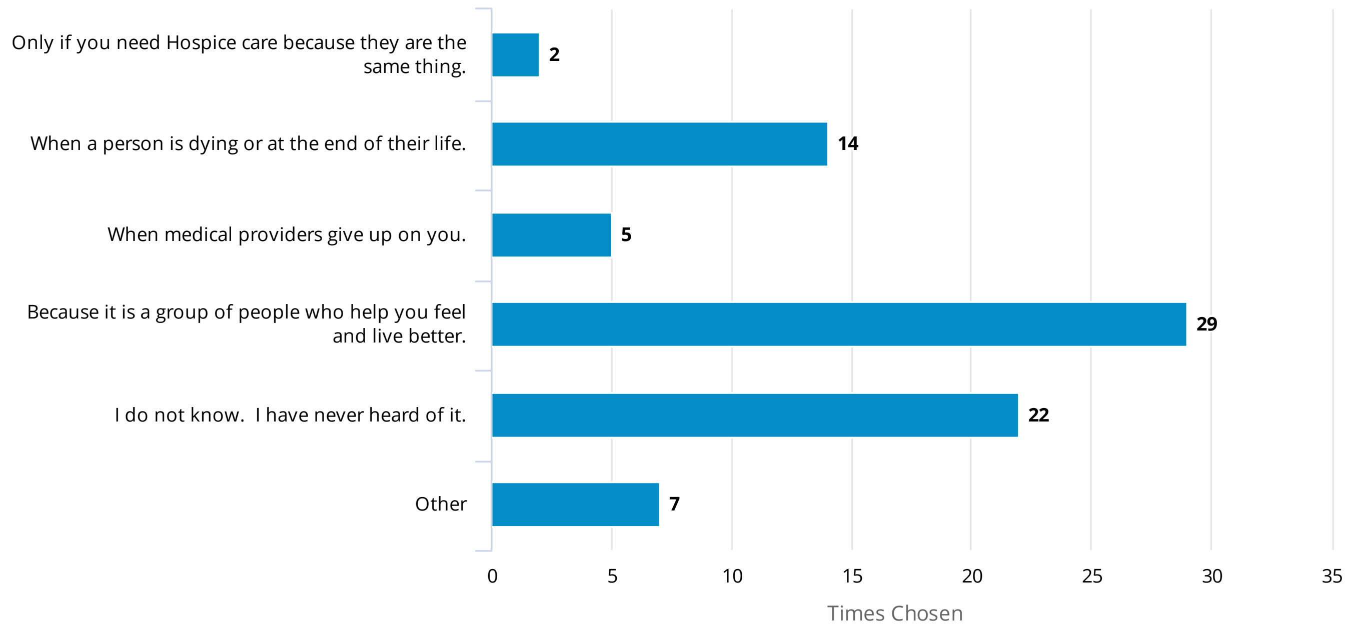

"Other" text answers:

People who can advocate for you that are NOT your parents

Answering as parent of EB children, I used to work in hospice

if you need pain medication to make it through the day.

Palliative care here is for those with a permanent chronic illness that needs pain managment abd other doctors fear losing their license when they are regularly providing opioid/narcotic medications regularly to one patient. It's an epidemic here where many abuse it and doctors don't want to prescribe anymore. So we are forced to seek these end of life type doctors to cover our pain management needs.

Keep a patient comfortable.

Palliative Care is the caring, maintenance and aid needed in dealing with life altering chronic illness to establish the best quality of life and care within reason

To improve quality of life whilst addressing pain issues for life limiting diseases.

9. Did you know that Palliative Care encompasses the care during any chronic life-limiting/threatening illness and it is not limited only to treatment at the end-of-life and differs from Hospice Care?

Number of responses: 71

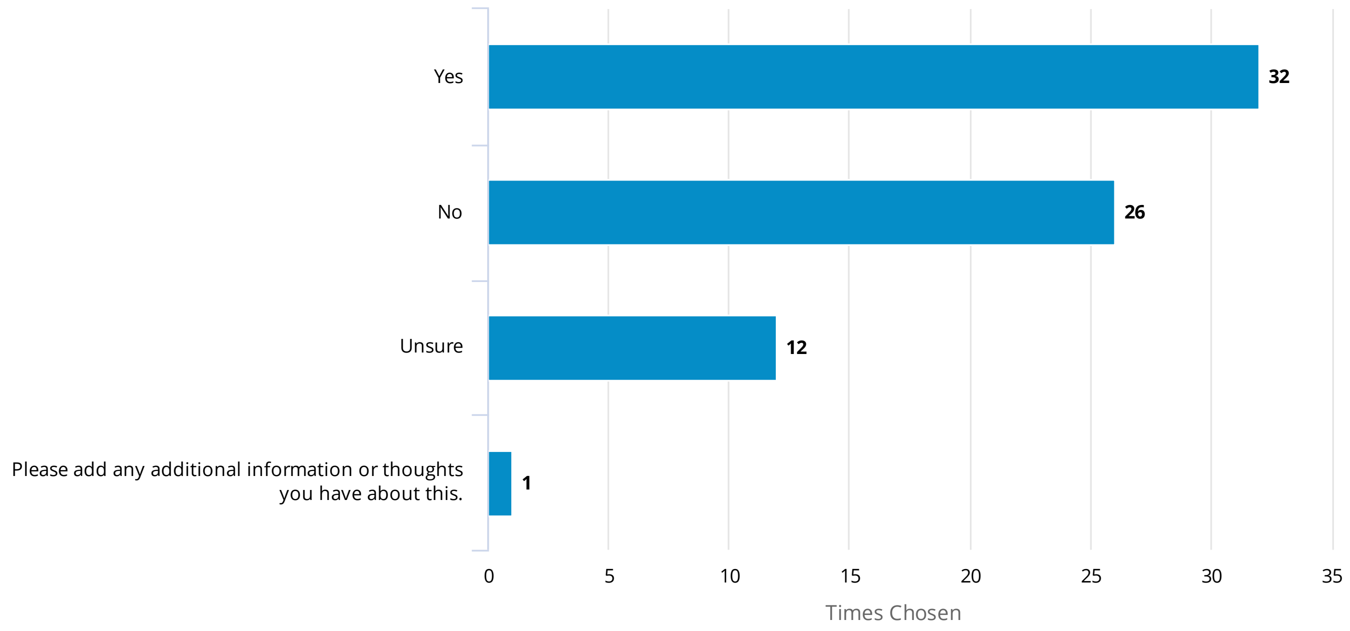

"Please add any additional information or thoughts you have about this." text answers:

Just found out recently!

10. Has anyone ever told you that all treatment of EB is palliative?

Number of responses: 71

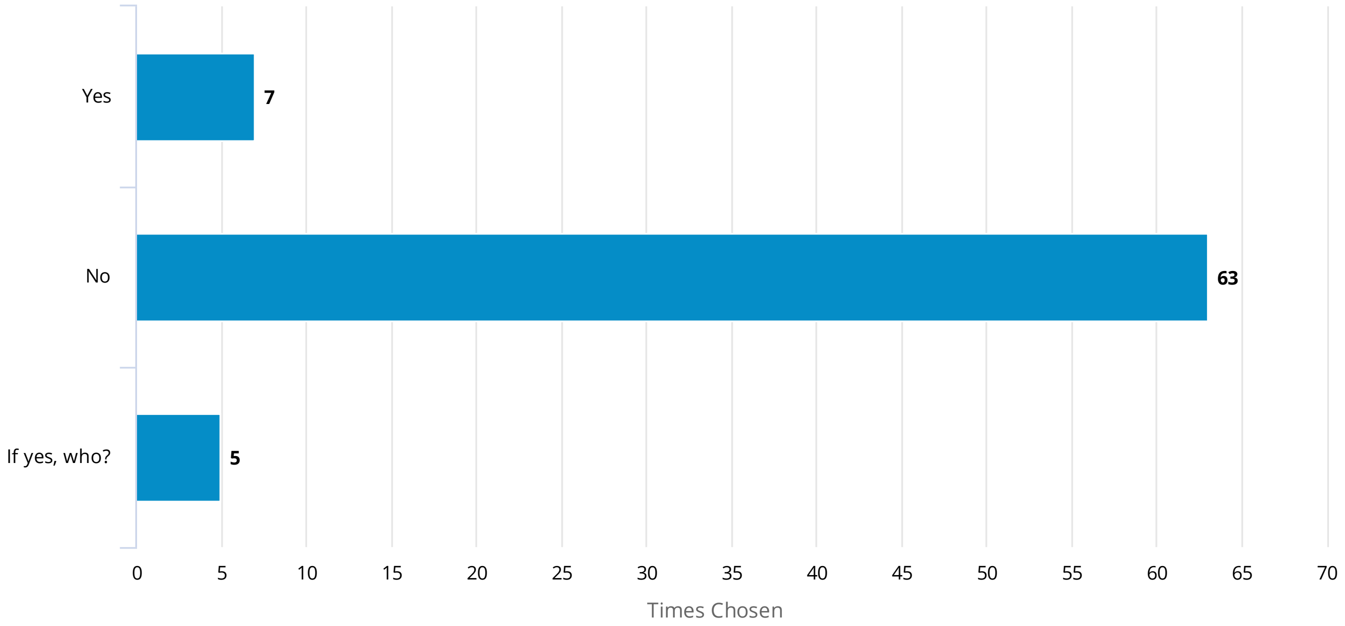

"If yes, who?" text answers:

RN caregiver

But I understand this concept

doctors, other EB families, etc.

All of the specialists we have been able to have Archer meet with - and also, based upon decisive reasoning of the situation as there is no treatment or cure

Dont know about it

11. When were you told about Palliative Care as a treatment approach in your care?

Number of responses: 71

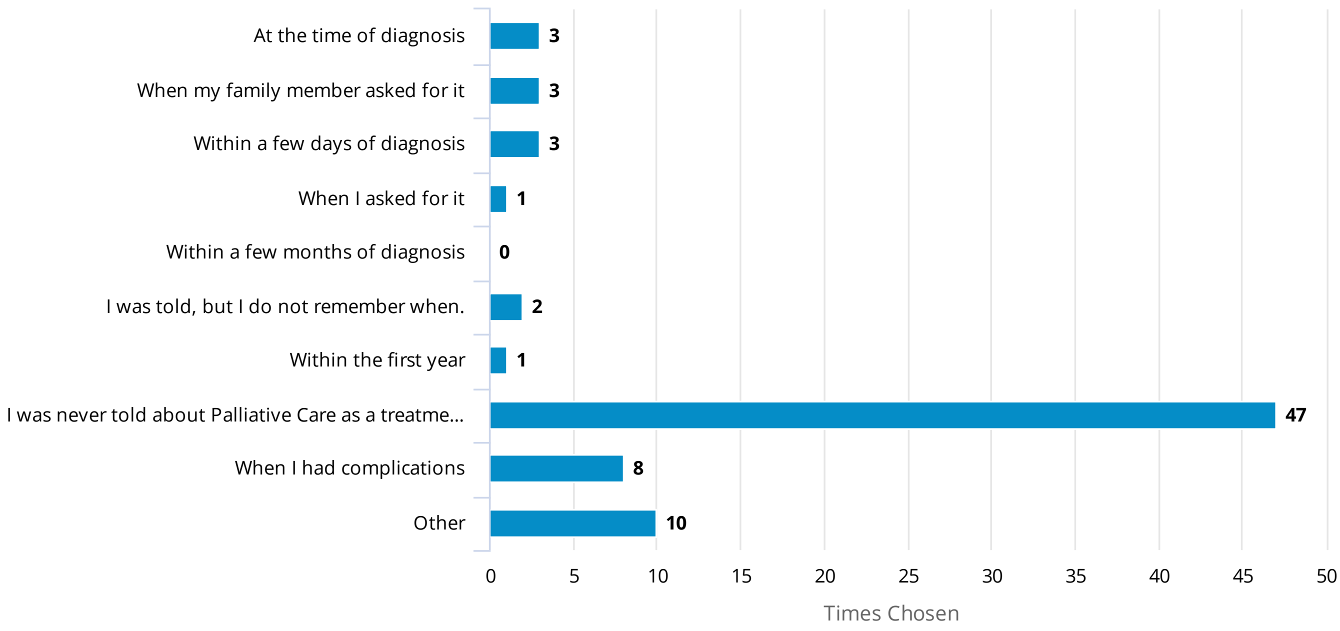

"Other" text answers:

by family members and RN caregiver

I wasn't told

I only know about it through my Facebook support group

But my daughters EB presents mildly and they are not significantly impaired by it

when I needed pain management services

This survey is the first I've heard of it

Never told

When my doctors and regular pain management doctors didn't want to help me anymore other eb patients informed me about palliative care doctors being a good option to help since our condition is not going to naturally improve.

Other

Dont know about it

12. Are you now or have you ever received Palliative Care for EB?

Number of responses: 71

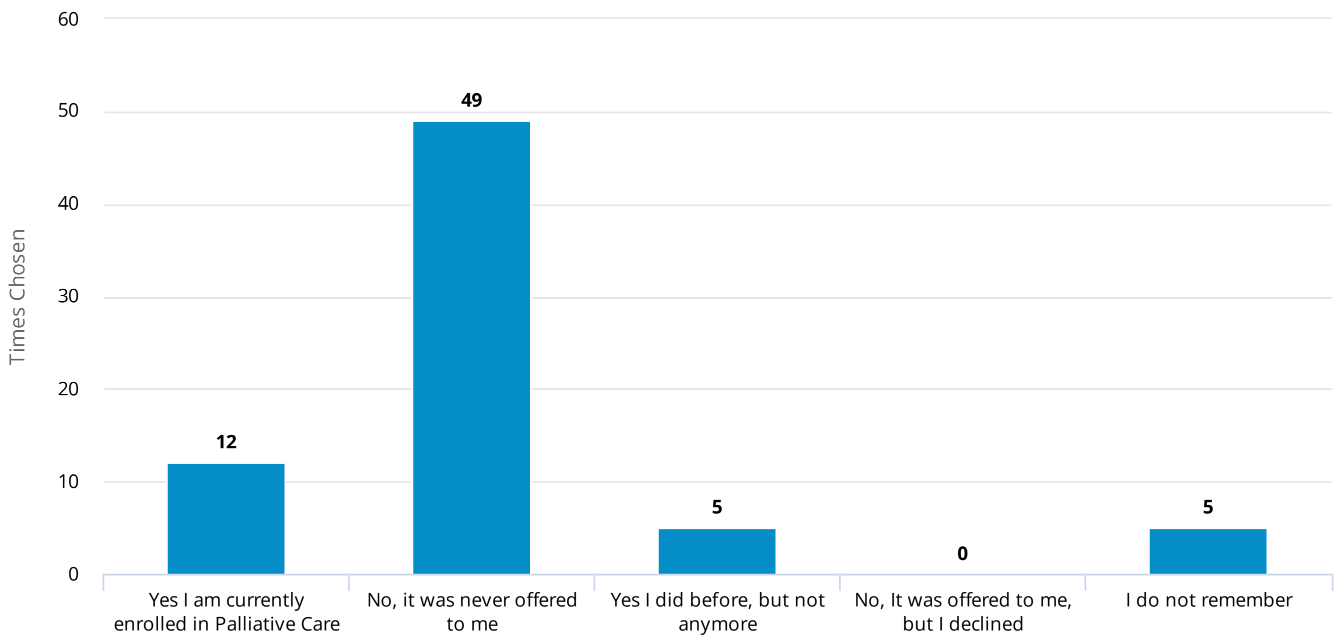

a) If you are enrolled in Palliative Care, looking back...

Number of responses: 68

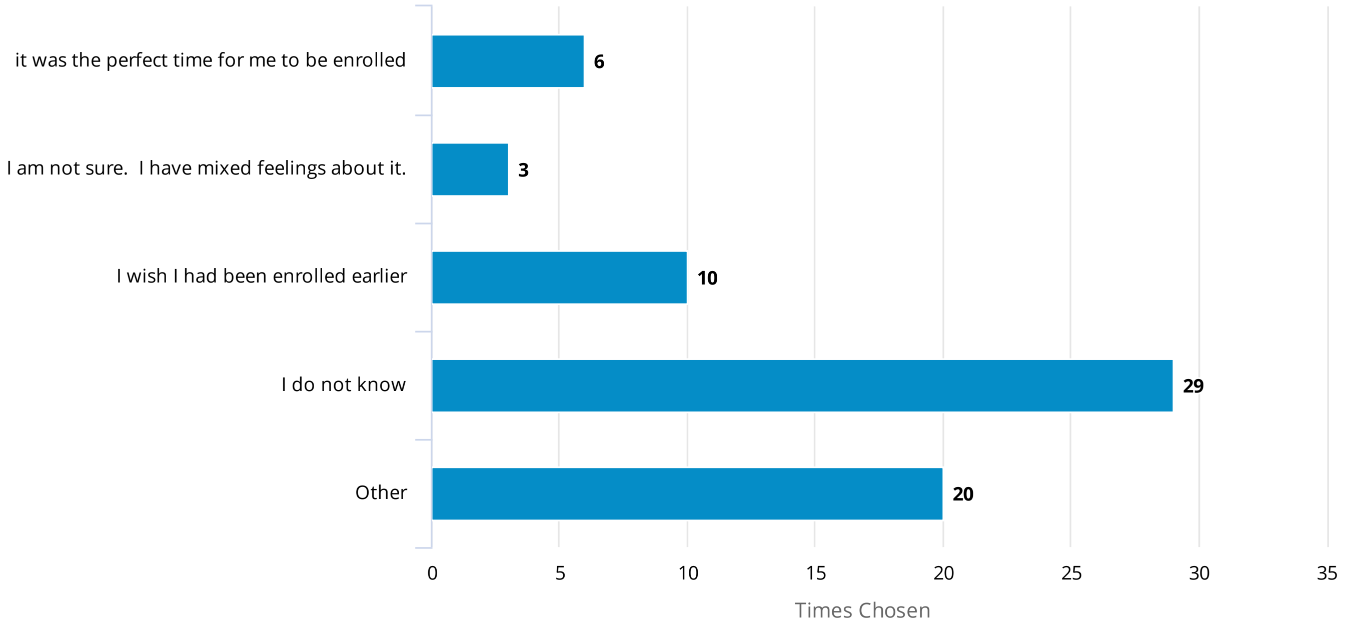

"Other" text answers:

Never heard of it
Not applicable
I'm not enrolled in any formal program
na
not enrolled
NA
Other
NO
na
Na
I wish that it were available to me now.
NA

N/A

Not enrolled

Other

N/A

Na

I'm not.

I was not enrolled

Na

13. What do you fear or worry about with respect to Palliative Care?

Number of responses: 69

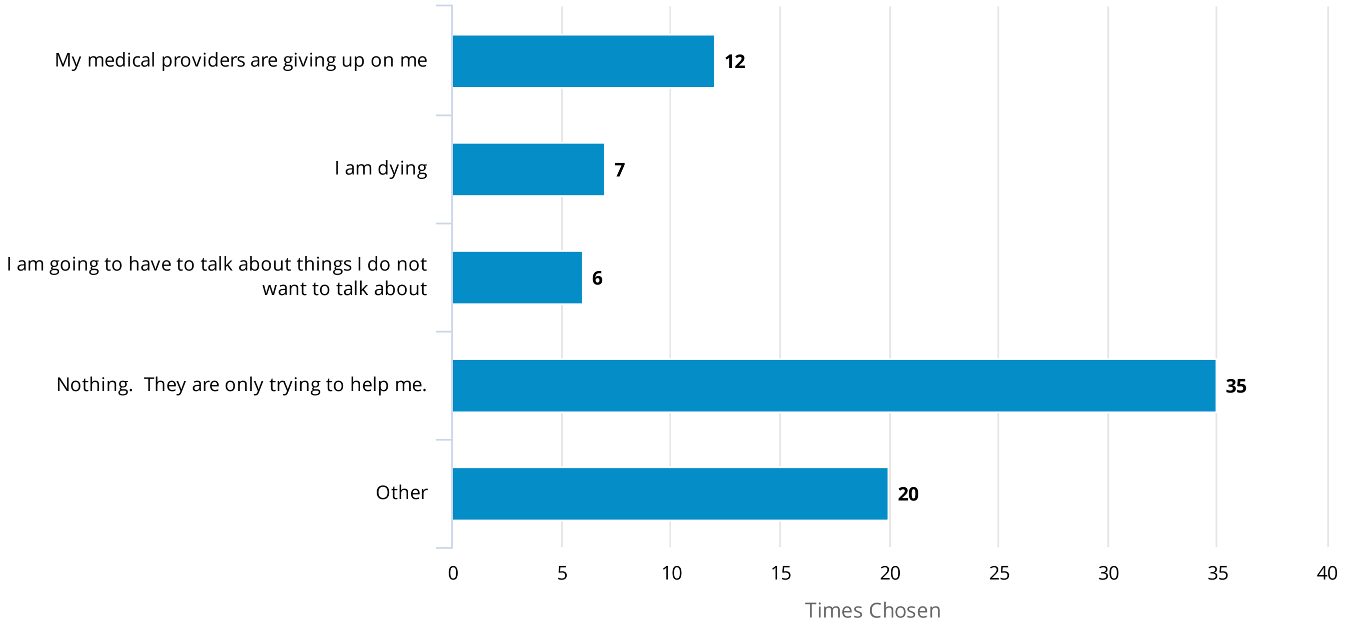

"Other" text answers:

I don’t know what it is

Nonapplical

I don't feel like this is for DDEB

NA

na

This would be my assumption if my daughters had a need to be enrolled in it

No

NA

Other

Other

I’m not sure but feel it could possibly benefit my children

Don’t know anything about it

Me outlasting my doctor(he’s old) when I’m unable to have the energy to fight for new doctor to follow me to the end.

That they won't help

Other

Harsh realities given with the situation and diagnosis

NA 3.5 year old

Dont know about it

A professional that doesn't understand EB properly force me to do something that will worsen my conditions.

My daughter currently uses pain killers before baths and cares

14. When do you believe is the appropriate time for a clinician who has diagnosed a person with EB to begin the referral process to Palliative Care?

Number of responses: 71

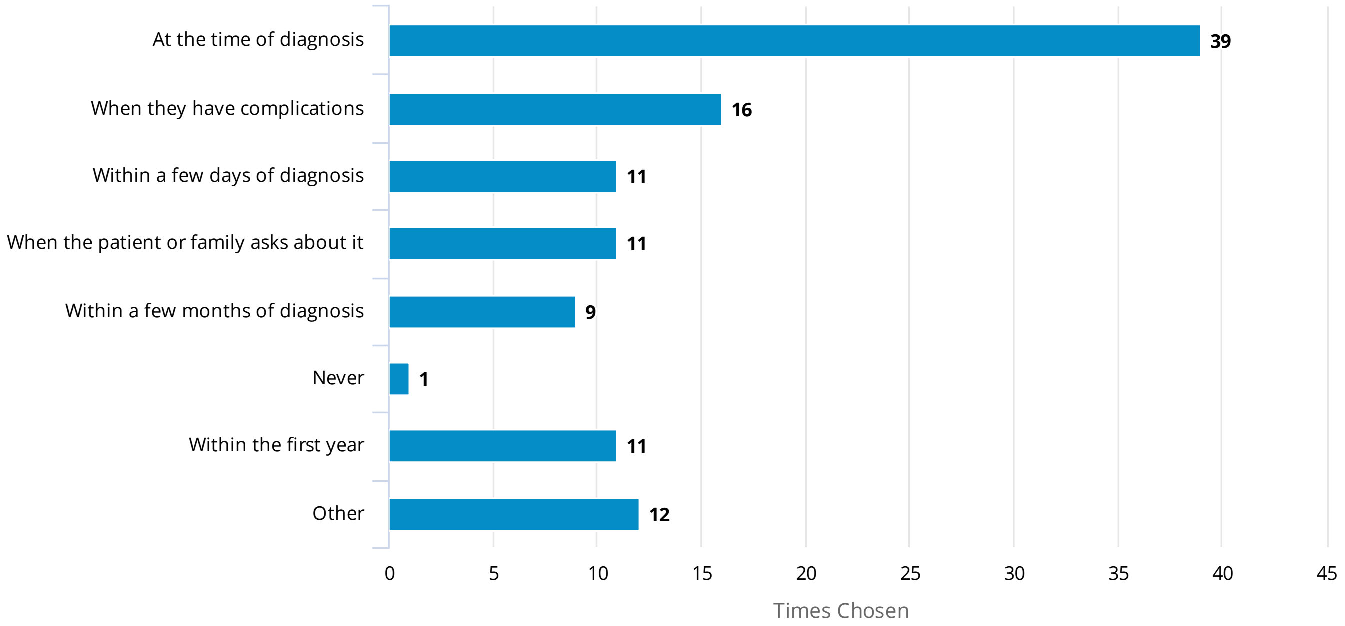

"Other" text answers:

I don’t know what it is

Depending on the form and how serious it is

when the need arises for pain management or psychiatric issues

If practical given the severity of the diagnosis/presentation

EB wasn't diagnosed until I was 17. Before that doctors didn't know what to make of it.

When the doctor believes the patient will benefit from such care

If the symptoms of EB is more than a general practitioner can handle. Eg: If the pain is getting worse

Depends on patient and family and their needs but definitely within the first year

They should always know about it but be pushed to seek palliative care when their eb becomes too difficult to manage with one doctor and one quarterly visit. And when more pain management is needed

I don't know enough about implications.

Dont know about it

An initial introduction to the team would be beneficial in the early stages of diagnosis and then Palliative care could float in the background until the patient shows signs of needing extra support whether it be when they have complications, extra pain or deteriorate.

15. What symptoms/issues have you found to be the most difficult to control in day-to-day care?

Number of responses: 71

Rank	Choice	Distribution	Score	Times Ranked
1.	Pain		609	67
2.	Itch		588	67
3.	Wound management		572	71
4.	Infections		503	68
5.	Mental health concerns (e.g., depression, anxiety, etc.)		469	67
6.	Access to other supplies		446	65
7.	Feeding		438	65
8.	Family stressors		437	65
9.	Access to medications		403	66
10.	Access to proper care		396	64
11.	Hydration		351	64
12.	Breathing		276	62
13.	Other		262	60

Lowest — Highest

16. What aspects of medication management have been most difficult for you/your family member that lives with EB?

Number of responses: 69

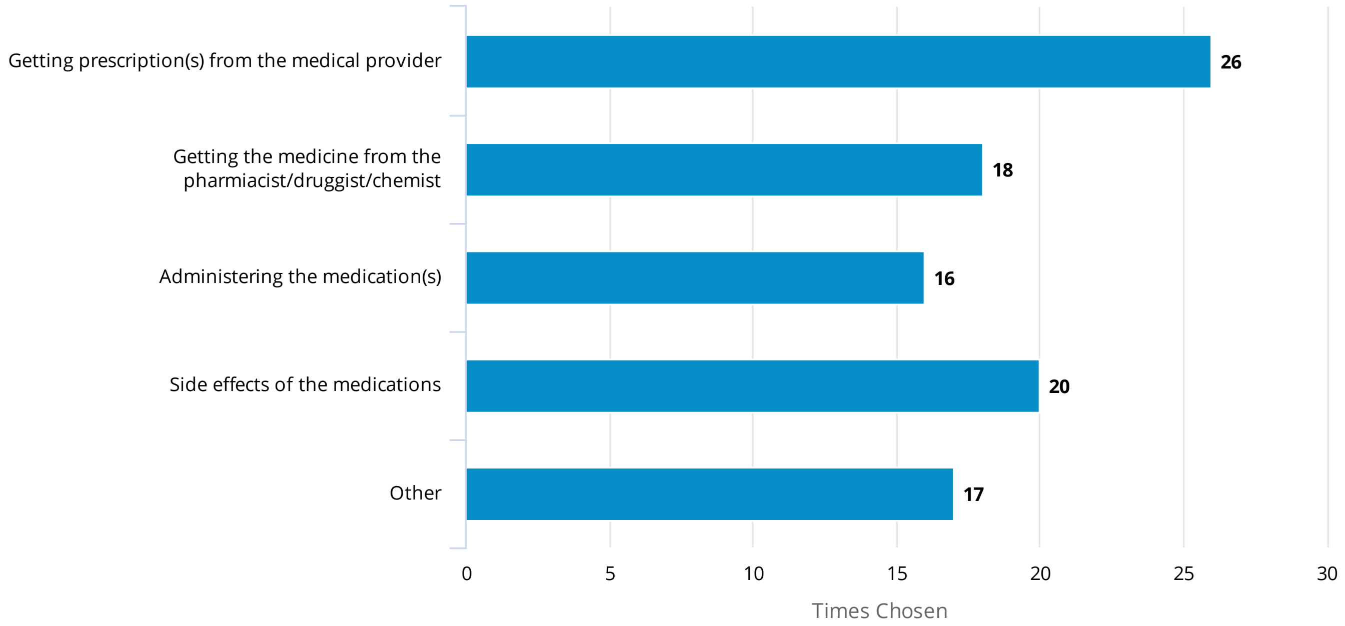

"Other" text answers:

Getting prescription(s) approved by health insurence
None
Getting compounded liquid zinc formulation/coverage for supplements, Miralax and OTC wound supplies
None
NONE
None
I have to manage for the most part on my own with the help of my wife.
None
Insurance coverage
Other
Trying to figure out all optionsn getting the clinical prior authorizations from insirance

Insurance coverage and time restraints

All have their peaks and valleys as well as challenges

The EB patient hates medication. Period.

Dont know about it

Once a solid patient / doctor relationship was established then accessing medication was easy and streamlined but it takes a dedicated medical team to ensure this happens.

Getting prescription of medicine that still work for me

17. What word would define your emotional needs when you were:

Number of responses: 64

An infant?	A toddler?	In Elementary/ Primary/ Grammar school years?	In High/Secondary school years?	A young adult?	A middle-aged adult?	An older adult?
Need Human touch	Need touch	Understanding	Support	Support	Support	Support
feeling of security	ideal	principal supporter	friend and ideal	free space, companion	friends, partner, companion	partner, friends, network
Support due to pain	Support due to pain	Support due to pain	N.a	N.a	N.a	N.a.
s,nb	gfg	gf	f	t	y	f
None	None	Teased, bullied, different	Teased, bullied	Self esteem,	Self esteem	Self esteem, depression
Painful	Painful	Anxiety	Embarrassment	Adjustment	Patience	Resignation
□	□	□	□	□	□	□
Nonverbal cues	Painful	Painful unfair missing out on everything being isolated, sad and angry for not being included	Painful and still missing so much feeling isolated.	Able to manage a little better yet still so much pain and having to be mindful in all activity	Mindful, purposefulness in any activity chosing to take part in.	Less active yet still painful isolated moments

An infant?	A toddler?	In Elementary/ Primary/ Grammar school years?	In High/Secondary school years?	A young adult?	A middle-aged adult?	An older adult?
Happy	Anxious	Fearful	N/a	N/a	N/a	N/a
Saftey	Saftey	Anxiety	Not applicable	Not applicable	Not applicable	Not applicable
Family's understanding about disease	social acceptance	Acceptance as a normal child	Understanding of EB challenges	Lack of friendships	marriage	moral support
-	-	-	-	-	-	how to live with pain and depression
I don't remember, harder for my parents then it was for me	I don't remember, harder for my parents then it was for me	Difficult time to be different	Got a bit better	Bit of a struggle to be independent	not there yet!	not there yet!
Love	Trust	Compassion	Acceptance	Empowerment	na	na
love and care	parental support, preschool willing to take me, developing friendships	community, friends willing to slow down so I can participate, being included, parents willing to let me be included	N/A	N/A	N/A	N/A
Medo	Medo	Ansiedade	Ansiedade	Alegria	Vivendo bem	Sobrevivido
strong attachment needs	social anxiety	n/a	n/a	n/a	n/a	n/a
basic	basic	severe	very severe	very severe	n/a	n/a
Lived in an orphanage and was neglected, most needed a family	Adopted at age 3.5, attachment and related-concerns were most consuming	Anxiety related to gtube changes, eye contact changes, and very frequent surgeries	N/A	N/A	N/A	N/A
normal	normal	normal	n/a	n/a	n/a	n/a

An infant?	A toddler?	In Elementary/ Primary/ Grammar school years?	In High/Secondary school years?	A young adult?	A middle-aged adult?	An older adult?
Wound care	Pain management	Wound care	0	0	0	0
High	Higher	Highest	NA	NA	NA	NA
love	kisses	hugs	understanding	love	kindness	gentle
fragile	encouragement	support	understanding	understanding	understanding	support
unsure	unsure	different	frustrated	worsening	frustration	na
G	G	Shame, awareness of difference, desire to be like peers	G	G	G	G
Need for comfort	Need for comfort, explaining situations, dealing with medical trauma	same as above with a deeper understanding	not there yet	not there yet	not there yet	not there yet
satisfied	satisfied	complicated	extreme	not met	not there yet	not there yet
-	-	Difficult	Challenging	Challenging	Challenging	-
bless my mother and grandmother.	I was beginning to know I was different.	Kids werre cruel.	Kids were merciless if they knew. PE teachers were not at all accomodating.	Afraid to get intimate with anyone especially women who might reject me.	Kept my condition as private as possible except for my wife and immediate family.	Same only the circle of trusted individuals is much smaller now.
None	Blisters	Blisters when walking	Blisters when walking	Blisters when walking and racking	Blisters under bra waistband feet hands	Blisters under bra, waistband, feet & hands
Met	Met	Not talked about	Pain management	Embarrassing	Sharing	Sharing
Entertainment	Entertainment	Anxiety	Depression	Anxiety	Not there yet	Not there yet
Security	Assurance	Freedom	NA	NA	NA	NA
pain	support	support	support	being normal	support	support

An infant?	A toddler?	In Elementary/ Primary/ Grammar school years?	In High/Secondary school years?	A young adult?	A middle-aged adult?	An older adult?
-	Pain management	Pain management	Pain management	-	-	-
Unknown	Unknown	Normalcy but it's ok to be different	I could have benefited from counseling to better adapt with differences and to better care for myself	Counseling occasionally	N/A	N/A
Painful	Happy	Lonely	Outsider	Different	Not there yet	Not there yet
Scared and anxious	Scared and anxious and grumpy	Scared and anxious	N/A	N/A	N/A	N/A
Needed love and support and patience	Needed love and support and patience	Needed love and support and patience	Embarrassing, trying not to stand out	Fine	Fine	Fine
Don't remember	Don't remember	Don't remember	Rejected	Depressed	N/A	N/A
-	-	-	-	-	-	-
Protection	Protection	Understanding	Depression	Ashamed	Stable	Na
Happy	Happy	Happy	N/a	N/a	N/a	N/a
Insecurity	Scare	Scare	Be sick	Issues for survive by herself	Scare	Issues for survive by herself
Don't know	Don't know	I was made fun of so much I missed school because I was scared to to	Missed school due to infections and my emotional issues fear of the outside	Anxiety and fear and lots of pain and depression	Anxiety and depression	Not there yet lol
Normal	Normal	Extra attention. Sensitive to new social interactions positive and negative	Difficult	Very difficult	Exhaustion management	Fearful
Dependent	Frustrated	N/A	N/A	N/A	N/A	N/A

An infant?	A toddler?	In Elementary/ Primary/ Grammar school years?	In High/Secondary school years?	A young adult?	A middle-aged adult?	An older adult?
Friendship	Friendship	Friendship	Friendship	Friendship	Friendship	Friendship
Na	Na	Na	Na	Na	Na	Na
No idea	Being comforted when injured, pain management	Wanting to be taken seriously when I was in pain and afraid of participating in gym class. Support on handling invasive questions	Acne and scarring management	Support on dating with EB	Dry skin management	Not sure
Good	Good	Good	10°	Good	Good	Good
Primal need to be fed and cared for.	Disappointed because I couldn't get around with other children.	Self-conscious	Embarrassed	Frustrated	Acceptance	Acceptance
Simpler	Demanding	Unknown	Unknown	Unknown	Unknown	Unknown
Pain and comfort	Sense of control, pain and itch, delays in gross and fine motor impacting socialization	Visible differences, not keeping up on playground, time constraints with medical care that impact social life, having a 1:1 at school	N/a	N/a	N/a	N/a
Normal	Normal	Becoming aware of my disease	Not there yet	N/a	N/a	N/a
Don't know	?	?	?	?	?	?
Touch	Trust in my own ability	Na	Na	Na	Na	Na
N/a	N/a	N/a	N/a	N/a	N/a	N/a
\sbsfg	Gsg	Gssfg	Gg	Gsgsg	GHrg	Grgrdhdfhd\f

An infant?	A toddler?	In Elementary/ Primary/ Grammar school years?	In High/Secondary school years?	A young adult?	A middle-aged adult?	An older adult?
In pain	stable	stable	emotional from pain and fear	emotional from pain and fear	NA	NA
touch	let me walk	let me play	love	liberty	not there yet	not there yet
Content	Content	Coping	Coping	Coping	-	-
angry	NC	NC	nc	nc	nc	nc

18. What non-medicine strategies/skills have you found to be fundamental to promoting Quality of Life and self-determination through your life?

Number of responses: 61

Text answers:

This doesn't define me. I define it.

open-minded, be more direct, be respectful, try to understand; do not always think what others think about you, do what you think is right; give power, and enjoy silence and nature; belive on a God; trust in other people;

Im not sure

er

Spiritual principles

Do not give up. Know your boundaries and limitations. Do let how others react to you/eb bother you. Forge your own future.

▯

Epsom salt soaks, Cbd salve and emu oil applied daily, thick padded socks, widest shoe available, purposeful activities chosen wisely. Cbd has been a god send. I would spend an hour after a 12 hour shift popping blisters and soaking my feet crying the entire time. Now I maybe get 1 or 2 blisters per shift and sometimes none. My son is still foolish and will not do the same skin regimen to prevent blistering.

Distraction from pain thru gaming

Wound care. Support, love, breathing slowly, redirection, icing, pain and anxiety support, great doctors,

1. Complementary Medication (taking bath with NEEM leaf water)

Being able to ask for help when I need it.
Luckily found a job working from home!

Seeing a psychologist/therapist weekly.

extra activities, meditation, listening to music

Apoio da família e Deus

A whole food blended fresh diet and a g-tube improved my son's skin and wound healing time drastically. It helped him get back on the growth chart, and greatly improved his quality of life. I think every person with a severe form of EB should have a g-tube. It no doubt has added years to my sons life. BUT there is a big problem with hospitals only advising people with g-tubes to use formula, while within the EB community there is a big push for a real food blended diet. My son was only drinking formula prior to his g-tube at 2 years, and when he switched to real food his inflammation improved greatly, all the old redness in his old scars disappeared and his wounds began closing in 1 week instead of 2 weeks.

playing videogames takes my mind off the pain

Deep breathing, finding things that are within control when everything else feels out of control

Distraction, formation and keeping of relationships outside the home, a connection to the general community

None

Relaxation techniques

play dates

Friends, hobbies, volunteering, education

taking one day at a time, prayer, supportive family and friends, being proactive, not playing the victim

Accepting what cannot be changed and changing the areas you have influence over.
Self compassion
(These are concept I imagine my daughters utilizing as they age)

Activities of interest, family time

I distract myself with hobbies as much as I can.

Supportive friends and family

I keep pushing through as much as possible. I do not think of myself as a handicapped person even though I am sure I qualify. My wife helps a great deal with physical and emotional support. If you give in you may never get up again, so I don't.

Bring careful

The right socks and shoes...preventive

Showing and raising llamas, participating in 4-H

Freedom to make real choices in regards to treatment such as when and whether to have limbs wrapped or free, ability to choose own clothing, and choice in when to initiate bathing routine.

Reducing complications

Creating strong friendships

I stay positive and remind myself that I can do as much as I allow myself

Loving people surrounding me including my Homecare nurse and family. Having silly and funny people in your life to make you laugh even you when you are sad. Doing something you like eg, bird watching, playing video games, watching movie, having friends come over.

Rest, planning ahead, correct footwear, knowing my limitations

Meditation, psychotherapy

I have never received medicine or supplies for my or my children's EB. We received a sample bandage and some larger needles to pop blisters for my daughter at dermatologist. We soak our feet with epsom salt, stay off feet, pop blisters, bandage if needed.

Meditation, bleach baths, ice packs for itch,

Be on computer and be able to talk and have new friends

Family and my own strength

Move it or lose it. Gravity helps a lot. Vibration stops nerve pain immediately. Looking at the bright side of EVERYTHING. Try and stay out of a dark low mindset because your mental health leads the physical well being of your body.

Keeping busy with activities (arts/crafts, playing with toys, music, etc.)

Sports, e. g. running
Activities with friends, e. g. going to the Soccer-Stadium
Holidays, e. g. Skiing or Running

Play video games

Not sure

do things my way, never cover the body with bandages don't pass too many body creams, let the skin breathe!

Prayer, worship music.

Establishing and building confidence through others in immediate and surrounding community.

Having the reasons for joy readily available.

Self coaching to understand this is not a punishment, and realize to stop asking why and start learning how.

Relationships. Meeting others with eb. Sleep.

Laughter

Live a life not EB.Work,socialize ,learn,....
try to avoid farmaceutical products, take care about your food

Trust in trying new things.
Breathing through pain/itch
Social community of people who play with and are not afraid of me.

N/a

aeert

To have a good solid family unit around you

Friendships, boyfriends, holistic therapies

- prayer
- essential oils
- aromatherapy
- reflexology
- cannabis oil
- counseling
- family/friend support
- sea water

meditation, relaxation, hypnosis

19. Which of the following have played a role in making decisions regarding your EB treatment(s)/life with EB?

Number of responses: 69

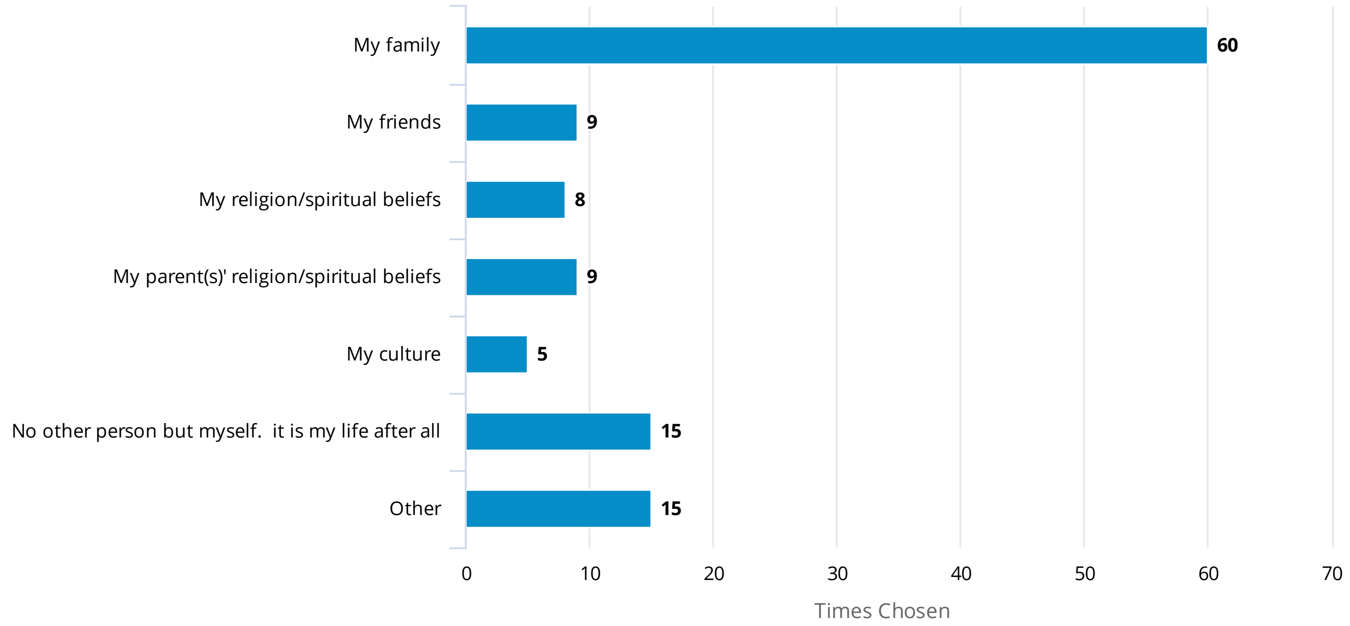

"Other" text answers:

Best solution for live quality with EB and therefore also the family
Social media eb groups
Myself
my present caregiver
Online support group, other caregivers advice
team at EB clinic
My nurses
Myself
My spouse
My strength
Doctors

Doctors

EB family

My doctors

Doctors

20. Do you think somebody with EB, but of a different ethnicity, cultural background, and/or religion/spiritual beliefs would make different treatment decisions than you made?

Number of responses: 70

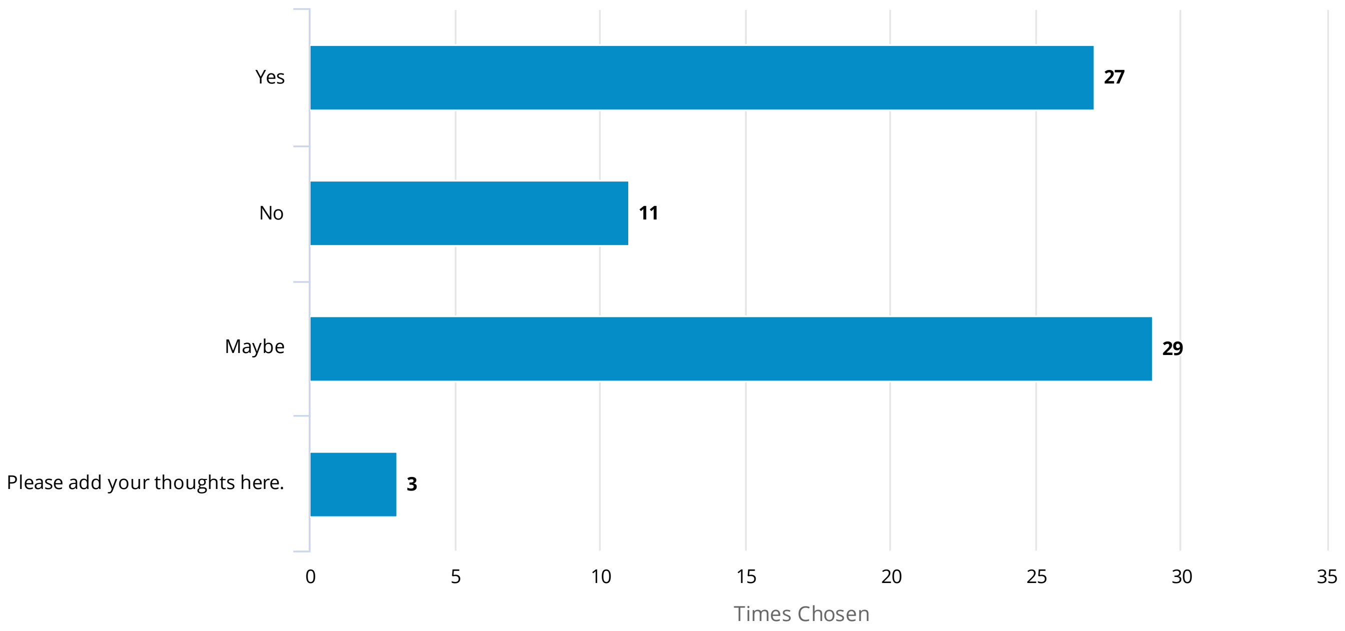

"Please add your thoughts here." text answers:

Sometimes but not always

Our cultural lense informs everything we do and have access to.

Please add your thoughts here.

21. Do you think your medical team considers and respects your cultural background, and/or religion/spiritual beliefs and personal values when providing care to you?

Number of responses: 69

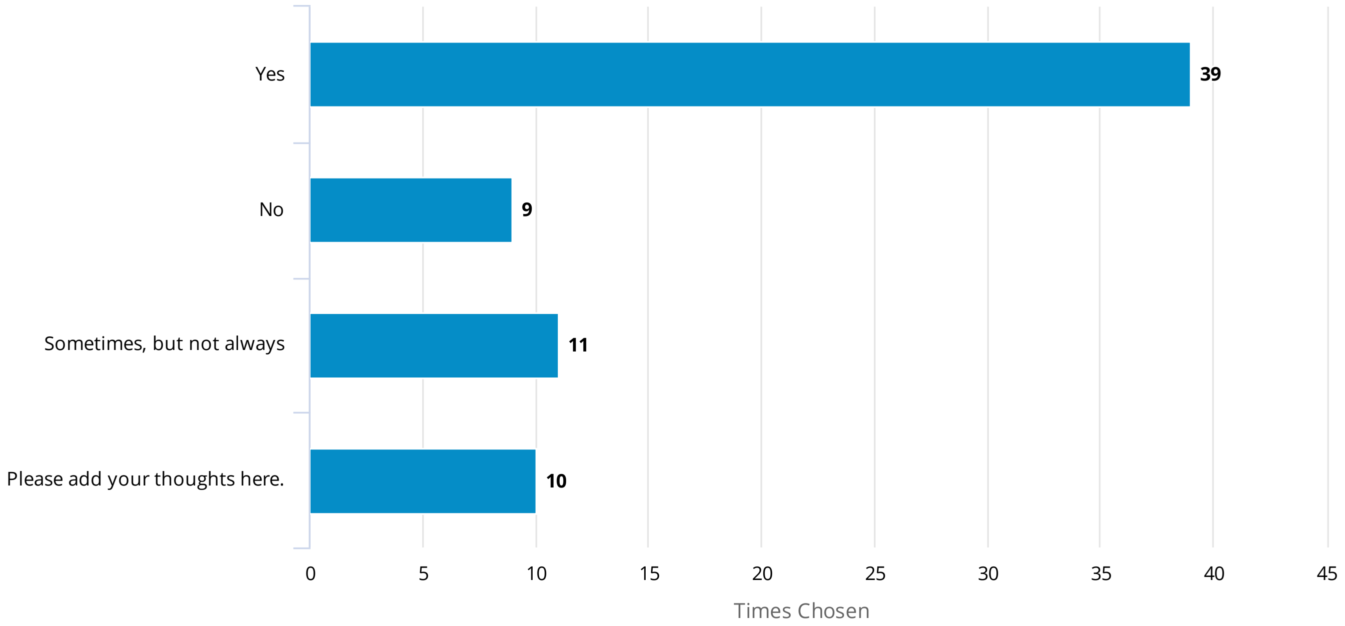

"Please add your thoughts here." text answers:

It has not been discussed.
Don't know
I think they would if we had beliefs that led us to make different decisions, but that has not been a factor in care
Not applicable
not known
No medical team
Doesn’t come into play
I'm part of the dominant culture in the US.
Please add your thoughts here.
Dont have

22. Please name the five most important things that define your Quality of Life

Number of responses: 60

Text answers:

Self sufficient. Self care. Pets.

Less pain, less wounds, less itch, good nutition, most possible movment

Support

fyt

Financial issues
Mental health issues
Exercise
Spirituality
Family

1.- Freedom to have the ability and mobility to get around.
2.- Still able to make my own decisions.
3.- Besides EB, relatively healthy.
4.- Have a home-care provider.
5.- Have a health-care nurse.

[illegible]

Cbd salve, anti blister socks, wide shoes, choosing purposeful movement wisely, resting

Xbox, family, friends, pain reduction, wound healing/reduction

Family support
Wound care
EB Physicians/ doctors
Medicines
Counceling

1. Availability of EB experts healthcare professionals
2. Availability of required medical supplies for wound care
3. Handsome monthly income to support disease management
4. Financial Assistance
5. Social and Moral support

pain and pain management, itching, the indignity of having people tend to me in all stages of daily life, having medical professionals who give up on my care

I feel like I live a pretty normal life. My EB effected me more growing up not being able to do sports of physical activities. It is effecting my life again as I am wanted to start a family and its a hard choice knowing the 50% chance.

Ability to live independently
Ability to cook/clean/work
Ability to attend college
Receive SSDI to afford to live alone
Ability to be in a relationship

Family, mobility, social interaction,

Deus, Autoestima, superação, vencer e alegria.

nutrition
access to wound care supplies
pain and itch management
love and support
positive outlook

no pain, medications, supplies available, not needing to go anywhere

daily symptom management (itch, vomiting, GI concerns), pain management, wound care taking as little of the day as possible, reducing need for monthly esophageal dilations

Pain, infection, wound care needs, nursing needs, mobility, adaptability, relationships

Being active
Pain free

Ability to play
Ability to eat
Ability to go to school
Ability to sleep
Ability to make friends

managing wound care, easy to put on and take off bandages, being able to play outside or in the water

Family
Well being
Community
Hobbies
Education

relationship with God
mother
family
friends
career

Cannot answer for my 5 yr olds

Controlled pain, controlled itch, proper nutrition/hydration, happiness, time to just be a kid

Free from infection, minimal itching, free from or limited pain, friends, shelter

Independence, self supporting, great family support, resources readily available, love and acceptance

Practicing my faith.
Loving wife.
Determination for us to have the best life possible realizing there are certain aspects of EB we have to work around.
Helping others less fortunate than I.

Self care
Wound management
Pain management
Religion
Family

Ability to train and show llamas, ability to make my own decisions regarding my medical care, ability to walk, ability to be independent, ability to handle my own medical care schedule.

Family
School
Friends
Fun
Choice

lees pain, getting medical care, getting medical supply

Family
Self care
Friends
Faith
Sports

Loving and caring family
Loving and caring home health nurse
Feeding birds in the backyard and watching them.
Caring for pet fish
Playing video games and watching shows in iPad

Health, time alone, family, financial stability, love

Being happy
Manageable pain levels
Having friends
Having a purpose
Financial security

Able to do things that others can. Run, walk at the zoo, play sports, wear any shoes we want to, play with kids.

Mom
Dad
Physicians
Teachers
Other family members

Meds
Medical supplies
Special wheelchair
Special bed
Computer

Family
Bandages
Friends
My docs

Relationships
Goals
Productivity
Good hygiene
Stability

Family, control, pain, choice, understanding

Family, Running, Friends, Sports, Mountains

Family
Friends
School
Phone
Bed

Freedom from pain, mobility, appearance, freedom from itchiness, emotional well-being

accept me as i am .. have a normal life ... have family ... get married .... have children

Pain free
Good mental health
Ability to work
Ability to care for my family
Free from scars

Comfort of Love
Confidence from Education
Ability from Action

Worth from Belief
Inspiration from Experience

Friendships and family, ability to learn, ability to play, ability to eat what others are eating, manageable pain.

Independence
Feeling normal
Participating in activities
Love of family
Support system

Independance,access to dressings,understanding and support

Love
Social Group
Nutrition
Holistic health Care
Peppa Pig

N/a

heheth

Pain free or at least being comfortable
Being with family and Friends, good company
To be able to access the wider community
To be able to holiday with family

Have a job, a house and a car; my knowledge about EB; being able to take care of myself on a daily basis, receiving dressings and other supplies from the local government, multidisciplinary EB team

- faith
- purpose
- belonging
- relationships
- learning

Itching is the big problem of that disease that sometines drive everybody crazy.

23. Has anyone talked with your unaffected family members (e.g., siblings, sons/daughters, extended family, etc.) about EB with respect to Palliative Care (including end-of-life care)?

Number of responses: 68

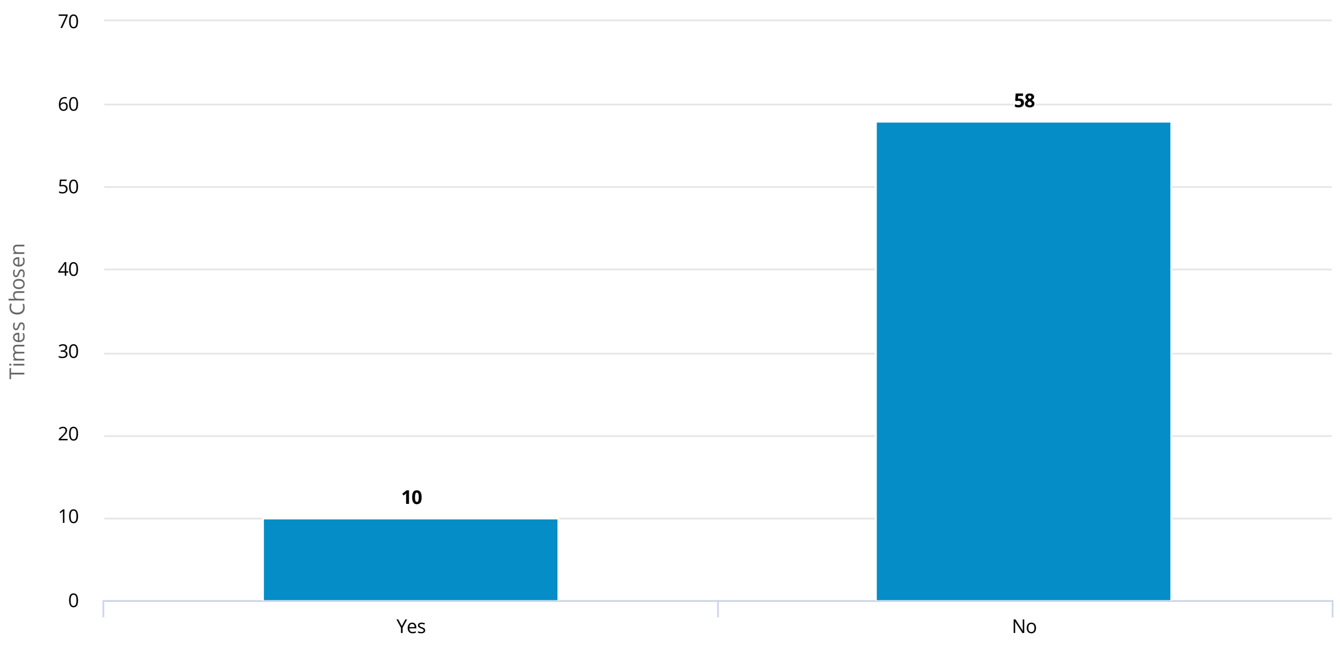

a) What did you like about this?

Number of responses: 6

Text answers:

Basically about also this kind of possibility.

That they understand better what I go through

I informed them of my daughters condition

The forthcoming honesty to tackle a stigmatized label

ngdfn

They kept us informed along the way so we didn't find it confronting or overwhelming

b) What could that person/team have done better?

Number of responses: 5

Text answers:

Not talking around a possibility about a hospice, instead of palliative care possibilities in first place.

been more patient and understanding

Our palliative care team has been one of our strongest foundations throughout this transition

\ddg\g

Informed us about the end of life breathing challenges and struggles

24. Has anyone talked to you/your family about death and dying as part of an ongoing conversation?

Number of responses: 69

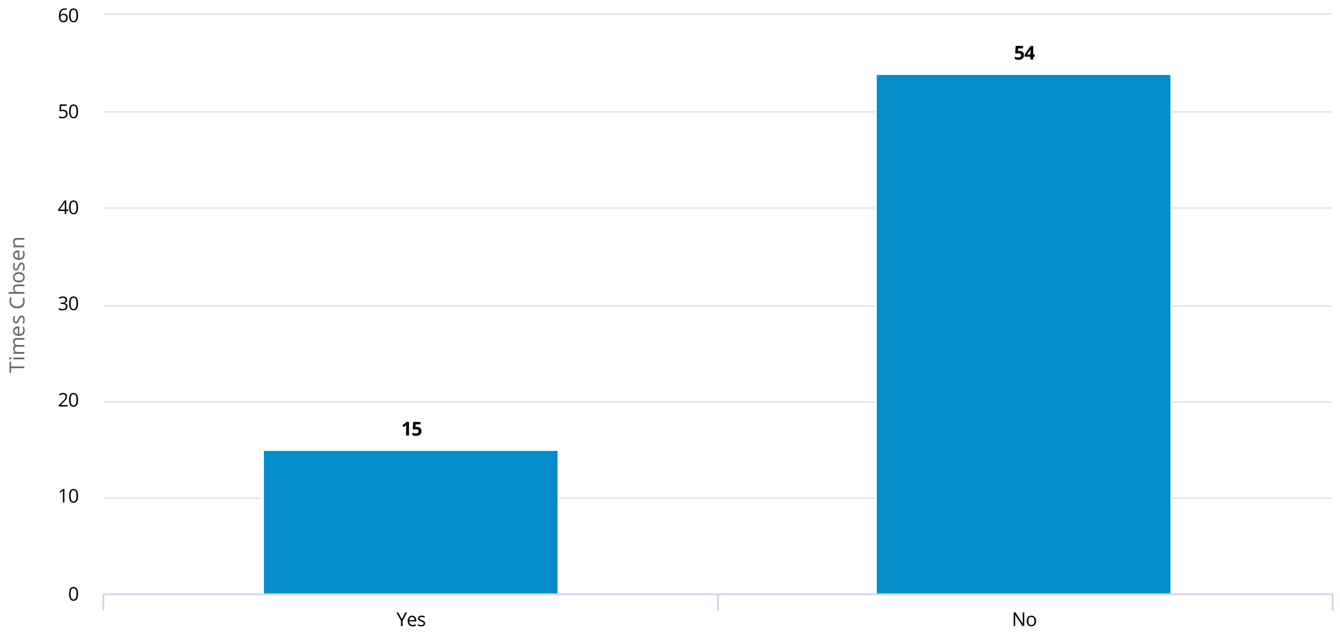

a) At what point in your care did they do that?

Number of responses: 15

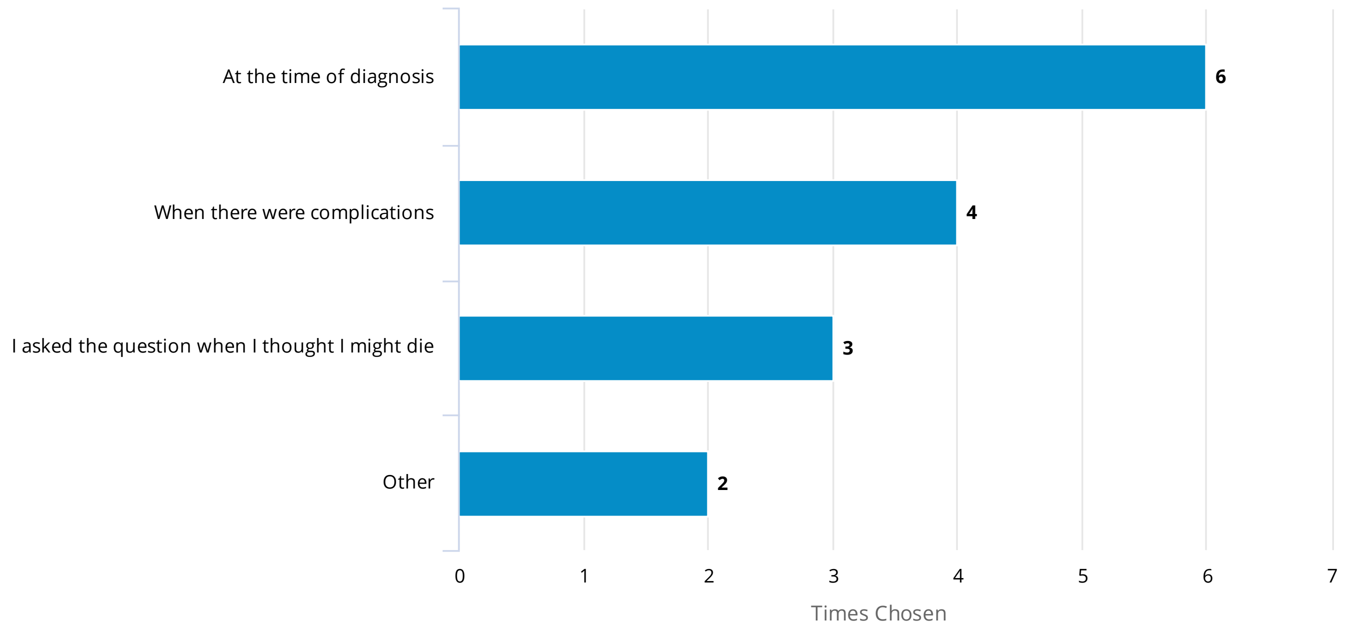

"Other" text answers:

At psychotherapy

Other

b) Who started that conversation?

Number of responses: 15

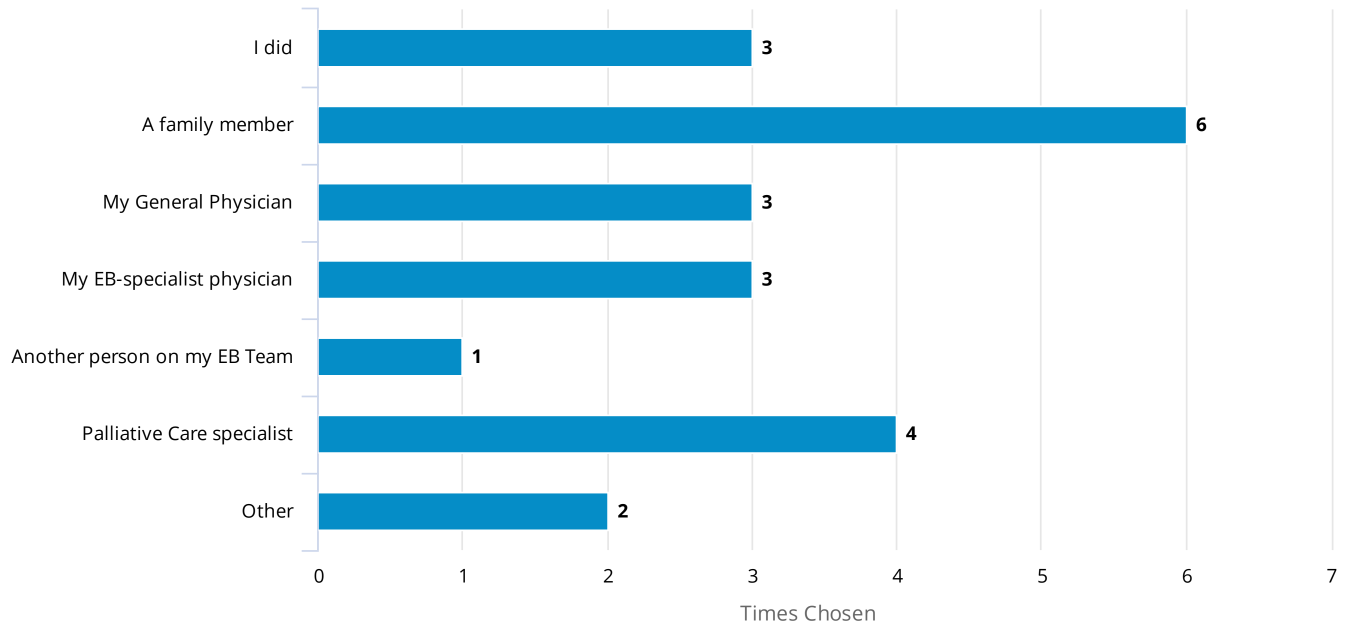

"Other" text answers:

Delivery drs/ eb specialists later when genetics were done
Other

25. Has anyone talked to you/your family about implementing an Advanced care plan, Living will, Last will, or Legacy?

Number of responses: 53

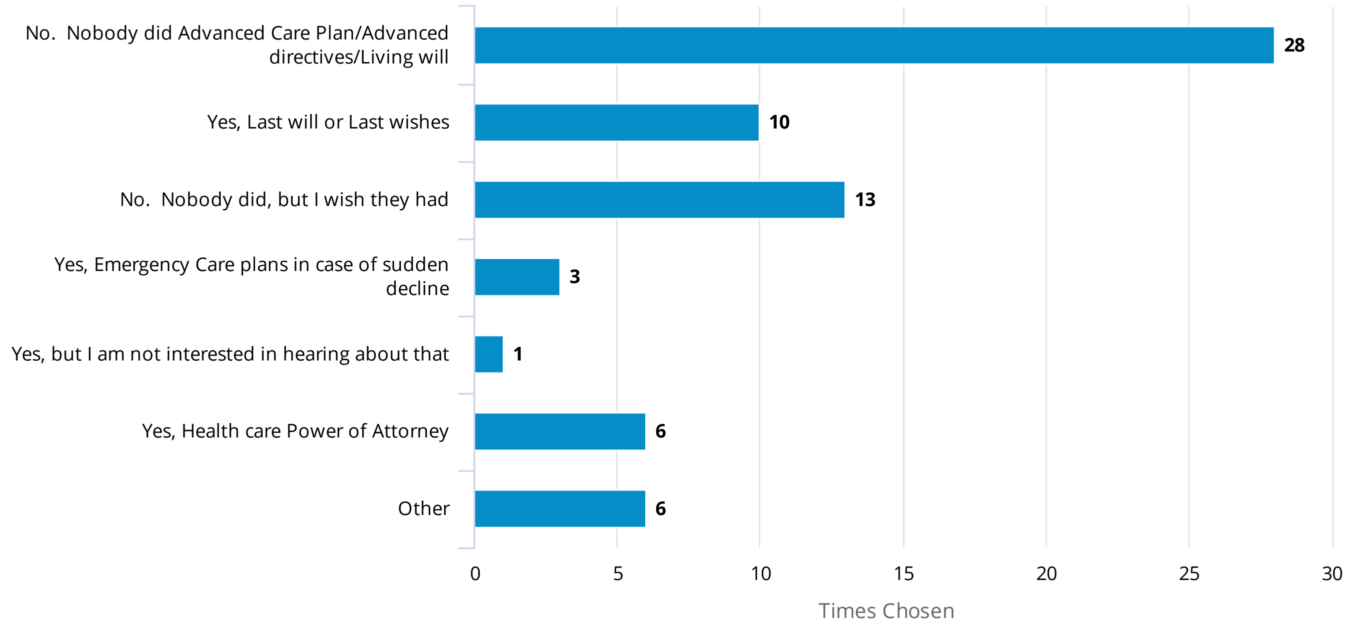

"Other" text answers:

- Special Needs Trust
- I am a child
- I know about this due to my background as hospice social worker.
- no one talked to my family about it but my parents researched it
- I took this upon myself
- Mom is fully aware of planning issues for child with EB

26. If you have an Advanced directive/Care plan or a Living will, what could your clinicians do to help you better?

Number of responses: 58

Text answers:

- No
- Try to clarify the induvidual real needs for your Quality of Live for the Right direction/ Actions.

Better support

DNR is my wish

Keep me as comfortable as possible.

▯

Not applicable

Talk with teachers and educators to help them understand what is important in life to ME....not to them.

N/A

Right treatment with positivist and hope

don't know if they would try to get better educated about EB

I dont know

na

Don't have one

Usar células tronco

we do not have this

n/a

N/A

it is my child so discussing changes in plan as health condition and needs change

Not sure

NA

do not have

Ensure the plan is followed

have one on my own not from EB team

Z

do not have one

Make sure that my wishes are followed if I can't communicate to them anymore.

NO

Not

Nothing

Follow the plan? I'm not really sure what this is asking.

NA

take care of my wounds

Not sure

N/A

No

I don't understand these questions. They're poorly written.

I'm not sure

No

Keep me calm

Don't have one, but should.

N/A

Provide treatment strategies especially agaings itching

Na

Not sure

I don't now

N/A

Understand the uniqueness of each person particularly in EB being affected differently than the next. No two are the same.

Talk about it

N/a

Do what you want

Na

N/a

htdhtdhtd

Nothing. they were fantastic and very accommodating towards my needs

I don't have that

-

Not concerned, my daughter is 5

27. What was the best thing you were told by your medical community?

Number of responses: 60

Text answers:

Nothing

"It is as it is." (- at a painful Feeling situation; - accept the Situation and make the best out of it for life)

Informing me about Debra

Nothing. I am more knowledgeable about my condition then they are.

I was a true example of strength and determination.

□

That I could qualify for a handicap placard

That there is hope for treatment

Keep doing what you are doing. You are doing a great job.

Life with EB is an on going painful journey throughout the life. We ll have to face it with courage.

We will do the best we can for you

I don't know

Getting PT to find exercises to do safely and get some fitness for my long term health

Don't know

Incentivo a fazer as coisas

To listen and learn from other parents/caregivers because they will know more than any doctor, since they are living it. That we can't determine his future, it is his story to tell. (From our dermatologist Dr Eichenfield in San Diego).

There might be possibly a cure someday

Not sure, I feel like I have to do most of the research and digging myself and then I bring it to my medical team

nothing. I always seem to be the bigger source of information

N/A

That I have EB simplex generalized severe, not RDEB

be sure to keep blisters drained and covered

I heal fast

giving me resources

Z

To enjoy life and be a kid as much as possible

Nothing.

Live life to the fullest, do not let this disease dictate what you can or cannot do

After so many years for not havinga doctor who knew what EB was, we finally found one.

Nothing

Medication instructions

That I am doing all that I can do to live my best life

To let the child with EB do whatever he thinks he can do and not limit him because we as parents are worried about his pain/skin/health.

I don't know

Not aure

N/A

None

I don't tend to hear a lot of good from my doctors.

I’ve mostly educated any specialist we’ve seen on EB simplex

We can try everything to see if it will work. Allow her to do what she can do without limits to the best of her ability.

You are strong

I would not live past 20 and here I am at 35

Never give up. We need you. And palliative care is best option for pain management

Never give up hope.

Don´t know

Na

Not sure

I don't remember

Keep hydrated and free from infection.

The honest facts about the challenge ahead

Aim for as much normalcy as possible.

EB is not one size fits all. Every patient has a different path.

Do what you want

Wait and See...equally infuriating and supportive.

N/a

\dhd\hd

We are here to help you

That i'm a person out of the statistics, because of my age and quality of life.

I am often told by my GP that she has learnt so much over the years about approach to life and living a quality life through a diagnosis by my and my families example. She has also said that she has implemented these new mindsets into her treatment of others. This has been the best thing to hear from her - that our families’ positive example of approach to disease is reaching others.

to not hesitate to reduce pain as much as possible, in preventive, to avoid blocking situations.
Once my daughter said "i want to die". A docto, much later said to me "imagine the pain to make her say this"

28. What do you wish you had known or your medical community would have told you that you know now?

Number of responses: 57

Text answers:

Eb isnt the end.

Overview about relevant runnig clinical Trials an possibilities where attend.

There is hope

I don’t find them helpful with my eb. I only go when I have infection for antibiotics

I was not diagnosed with EB until I was in my early twenties. No doctor/physician before that knew what it was.

□

To try to always live your life not sit back and make excuses

That meaningful treatments are much farther away.

Not sure

That EB has multiple complications and involvements

About PGD IVF

That palliative care isn't just for end of life planning

Don't know

Células tronco

That CBD balm helps tremendously with itch and pain, reduces inflammation and shortens wound healing time. This is the ELEPHANT IN THE ROOM. Everyone in the community knows, but none of the medical community seems to know.

every patient is different

About palliative care

how important maintaining range of motion is to keep body moving, infection control,

N/A

NA

there is help out there in terms of supplies and moral support

More about aging and EB

long term effects

Z

preparing for end of life care

I can't think of anything.

?

Not to be embarrassed

I wish I would’ve listened when I was told to wear hand splints

NA

know more about EB

Not sure

N/A

Day to day living tips

Doctors will give up on you and you'll have to find new ones whether you want to or not

There is more than home care treatment

More wound care guidance, discussion around infection

More understand about EB , more training to doctors and nurse

Do not use adhesives

Get a gtube young. It’s not an easy decision as an adult to get.

Everything that is or is not available to a patient with EB (eg: most insurance doesn't cover bandages, so here are ways you can get bandages... eg: here is a list of all the types of care you need & have access to, such as palliative care or pain management)

Don´t know

Na

How to manage pain when I was younger

Everything about EB

N/A

A bit more in regards to the effects surrounding all involved, and the multi layered facets that chronic illness attacks besides just the physical attributes.

Would appreciate more anticipatory guidance as we move through life stages. School in particular.

My daughter could live without bandages on 24/7.

I should not follow all doctors advices

People on FB are sharing their worst days. And, we only have statistics from people who are accessing care, often the most severe cases.

N/a

\hdhd

NA

That I would be able to live a great life.

My family was told I wouldn't make it to see my first birthday. I wish, for my parents' sake, that that information would've been less incorrect. I wish my parents would've been told that a quality of life could be obtained, just with a bit of extra work and commitment.

to not hesitate to reduce pain as much as possible, in preventive, to avoid blocking situations

29. What group(s) of people should be the focus of education resulting from this guideline?

Number of responses: 67

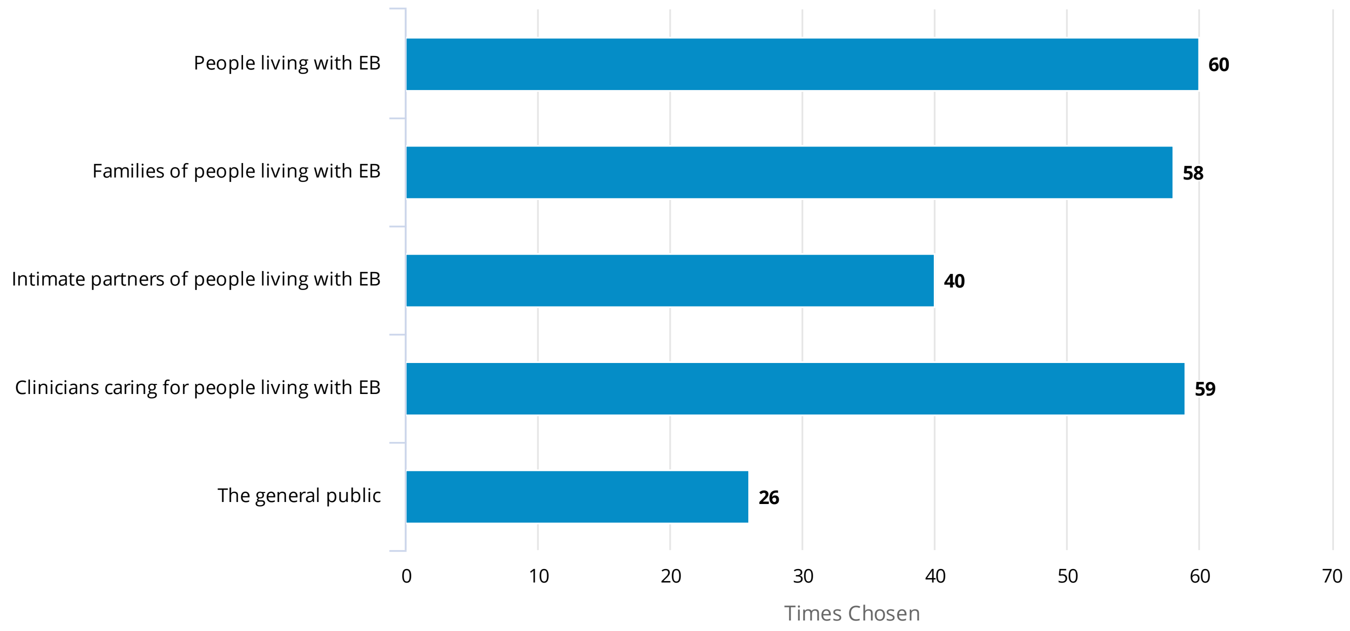

30. Is there anything else regarding Palliative Care for people living with EB that you would like us to know or consider?

Number of responses: 58

Text answers:

No

Pillative Care - which ways of care / support are possible, available Information online for overview and to get in contact, available offers near-by

No

I have no clue what it is

Do not know what Palliative care is.

▯

Eb patients pain tolerance is totally different than that of a normal person. Medical community should not treat eb patients as drug seekers. Our pain is real and needs treated

I would like educators and schools to learn "trauma informed care".

I don't know what it is really, even now. So spread the word or educate families.

.

Palliative care in EB is the most considerable sphere that is completely neglected in my state. We do nit have EB experts and the general physicians and nurses are unaware of the associated complications. They do not pay much attention to the people living with EB, especially in their last days because there is no prognosis which further lowers the moral and determination to live. Owing to this reason EB patients become a burden on the family and the family support system starts giving up.

don't know

I don't know

Just because a person w/EB doesn't present looking severe, their life is still very limited and they can often be overlooked or dismissed by medical professionals who don't know that person may be living very isolated to avoid causing damage to their body and be dealing with serious mental health issues as a result.

No

Não

Nutrition and a g-tube are the number one tools for managing EB.

no

Transitions Kids in Raleigh, NC USA has an amazing model for palliative care and we have been really happy with them. We had a very hard time with EB clinic pain team prescribing pain meds and made me (mom) feel like I was asking for something outrageously outlandish when all I was asking for was a PRN dose of oxy to use 1-3 times per month for my severe RDEB son.

Palliative as a word has a negative connotation. Perhaps renaming it to long term care or something of that nature. Or even EB palliative care so that it is specific to EB which is different to most other situations given the long term needs of the patients.

No

This should be age appropriate.

nothing

Should be one in Canada

having a dentist who knows about EB would be the best thing that could happen. there are no dentists that have a clue

medical professionals who have no knowledge of EB don't believe me when I tell them what I have.... very frustrating

Z

no

Palliative Care should be available to people who can't make it to an EB clinic. We don't live close to one and now that I'm an adult, it seems like no one cares about how EB affects my life.

I think you covered it.

...

No

NA

no

No

Not sure

No

No

No

It had been easier to live with as an adult because I know my limitations and are prepared if I exceed them. It does put limitations on life and what I can do but mostly this is hard to see my children experience growing up now.

Show the world we have nothing contagious and treat us with respect ,we want to live a normal life

No

Palliative care should be the ones who see we are not asking for anything but help getting through the day with less suffering. Any doctor should be willing to go above and beyond or think outside the box to help us achieve our goals with what little time we have here in the most severe forms of eb until there is a cure for eb. I am blessed my doctor guides me and lets me know the negative effects to the medications I feel work best for me while I try my best to work and get through a normal day. And I appreciate him offering new medications and to try new methods to reduce possible addiction and side effects to medications I'm taking. Having that great relationship gives me comfort in knowing I'm doing as good as I possibly can be with the diagnosis I'm given. And still able to achieve my goals at the end of the day I can't complain.

Our EB child’s pediatrician was under the impression that palliative care was only available for end-of-life care, so we had to push for the referral even from a medical professional. The only reason we knew about it was from other EB families. It needs to be more widely advertised as available and necessary as part of standard EB care.

No

Na

No

No

No

Real ease the stigma of the title - and educate to not just feel that it is a direct path to the end of life.

The change in rules in the US with narcotics. Role of cannabis. Focus on the social aspects and belonging in general.

No

Mom: I need to research palliative care. I've generally thought it was for terminally ill with a generally agreed upon life expectancy.

N/a

wtwt

No

Churches and religious people made things worse instead of helping. Holistic therapies and approaches help me much more, with both psysical and psicological aspects.
Doctors have a tendency to consider old literature and tell family members, at the time of the diagnosis, that they shouldn't worry so much about the child with EB, because it's going to die soon. Many mothers have told me that.
Psycological support, indvidual or in group, is fundamental for people with EB because family members without EB may not understand us.

I’ve found this survey very interesting so far. Whatever is put together I am sure will be a step in the best direction. Thank you.

It is amazing to see the different approches parents have regarding pain killers, even for "healthy" children with benign problems (growth of teeth por example). Some will give very easily pain killers, and others will give it really occasionnally.

Find solutions for itching should be a n°1 priority to improve quality of live.....

Derived from morphine (tramadol) might increase itching on some people .... think that what might reduce pain might increase itching is worrying ....

1. Are you filling this questionnaire for a loved one who passed away from EB?

Number of responses: 49

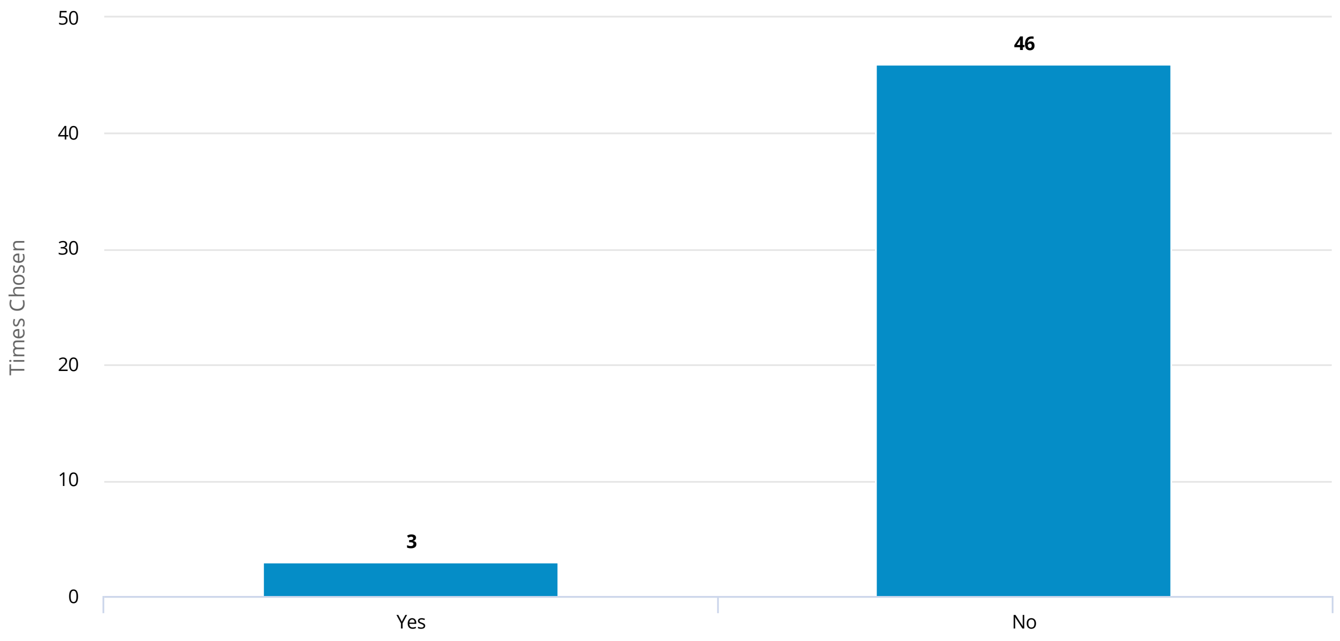

2. What symptoms/issues did you find to be the most difficult to manage at the end-of-life?

Number of responses: 3

Rank	Choice	Distribution	Score	Times Ranked
1.	Pain		32	3
2.	Breathing		28	3
3.	Infections		26	3
4.	Wound management		21	3
5.	Feeding/Hydration		20	3
6.	Access to medications		19	3
7.	Mental health concerns (e.g., depression, anxiety, etc.)		18	3
8.	Access to other supplies		18	3
9.	Itch		16	3
10.	Access to proper care		15	3
11.	Family stressors		13	3
12.	Other		8	3

Lowest — Highest

3. What did your loved one who passed away, family, friends or medical team do, if anything, related to legacy building?

Number of responses: 3

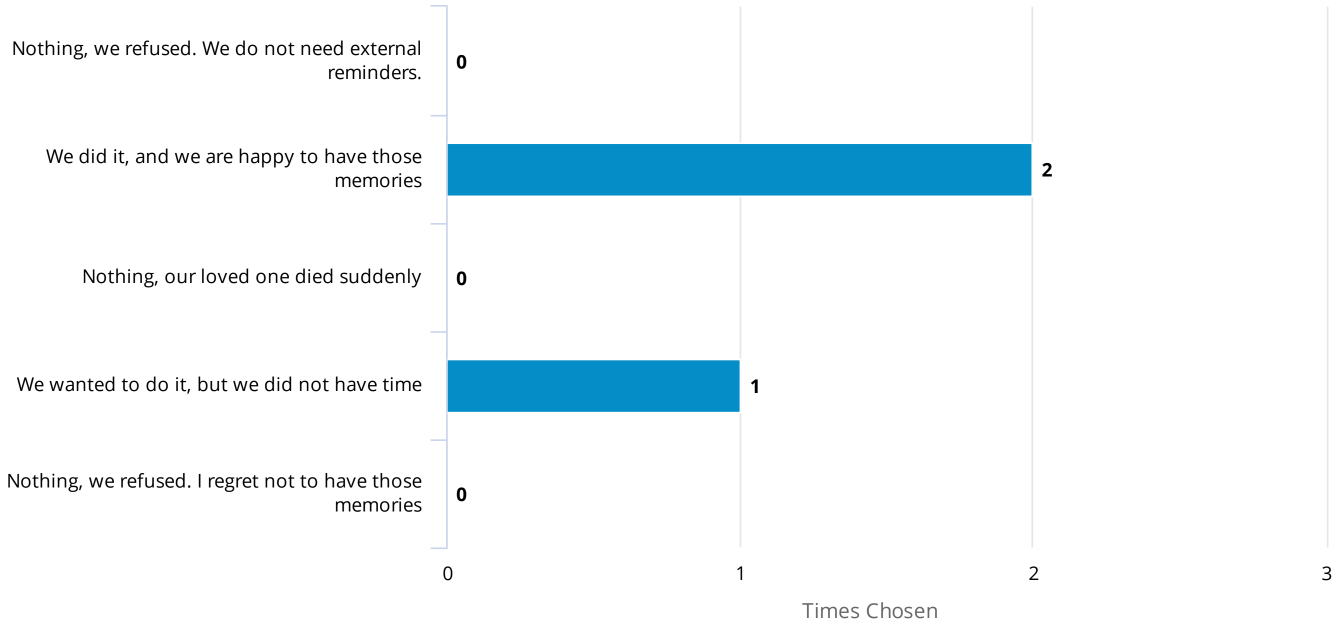

4. What type of support did you receive after the passing of a loved one from EB?

Number of responses: 49

Text answers:

No

(psychological)

N.a

I am an EB patient. I also lost a younger sister with EB. She passed away from EB complications along with other causes. No support was available.

Not applicable

Not applicable

N/A

Nothing

I answered no

na

N/A

Eu tenho EB

n/a

n/a

N/A

n/a

N/A

n/a

Received no support

na

Z

We made hand/foot prints/casts, had a service at the hospital where we collected memories/notes from caretakers/staff

NA

I don't know anyone who has passed any from EB.

No

NA

noting

N/A

N/A

N/a

This is the worst designed survey I've ever taken. This isn't relevant, but I still have to answer

Na

NA

?

N/A

-

Na

N/a

none, no loved one died with EB

N/A

NA

N/a

N/a

Na

N/a

Continuous emotional support from Palliative care and General medicine doctors / team. Two years on and we still have contact

not the case

When babies with EB pass away in South Africa and I’ve been significantly involved in their lives, I seek counseling during and after the passing.

NC

a) Was this adequate?

Number of responses: 3

Text answers:

no

yes

yes more than adequate.
I think we have been very lucky with the support received, it's exceptional

b) What changes would you like to see made that would best support families?

Number of responses: 3

Text answers:

easing the death or last day of life with EB by building EB clinics across the country equipped with best possible facilities

none

Introduction of Palliative care team after diagnosis. The purpose of the team to be explained to the EB individual and their family - it's not just because the patient is dying but more importantly can increase quality of life for the entire family unit during their journey.

Please enter your contact details:

Number of responses: 29

First and last name	Title	City and country	Institution	Preferred email address to use
Jennifer Rodriguez	-	BRIDGEPORT U.S.	1990	jennrod998@gmail.com
Richard Lopez	-	San Antonio - United States	-	mrzepol@yahoo.com
Muhammad Danish Hasan	Danish	Karachi pakistan	[illegible]	Danish.hasan@bankalhabib.com
Betty Jo Ulrich	Ms.	Columbus USA	Riverside Methodist hospital	Bjulrich001@msn.com

First and last name	Title	City and country	Institution	Preferred email address to use
Wendy Mansfield	Mrs.	Lee's Summit MO USA	?	w.mansfield@hotmail.com
FAIZA AMBREEN	Mrs.	Lahore, Pakistan	Debra Pakistan	faiza.ambreen1986@gmail.com
Emily Boros-Rausch	-	Moncton, NB Canada	-	emily.boros.rausch@gmail.com
Venus Stuhan	-	Phoenix USA	-	vstuhan@gmail.com
Luciana	EB	Cotia , Brazil	Portador	canoluciana@yahoo.com.br
Laura Dellicker	-	Chapel Hill, NC USA	-	lauradellicker@gmail.com
Bonnie-Jean Gale	-	Vancouver, BC Canada	-	bgale@novuscom.net
jan marhefka	-	hamilton ohio usa	-	jmarhefka@cinci.rr.com
Cyndi Daniels	-	Crosby, USA	-	cyndidaniels96@gmail.com
Kendall Lanham	Miss	Floyds Knobs, IN USA	-	Llamamama1998@gmail.com
Emily Smith	Mom to EB kid	Indianapolis	-	emily.storm.smith@gmail.com
Mukhtar Agoub	-	Misurata	-	moagob@gmail.com
Daniel Donnelly	Mr	Stafford	Mr.	dadonnelly@gmail.com
Tera Morgan	-	OKLAHOMA city United states	-	tlalbert111@gmail.com
Evelyn Naebe	Mrs.	Belvidere	-	evanaebe202@gmail.com
Michelle Hall	Ms.	Sayreville, New Jersey USA	-	Michellehall1572@gmail.com
Valerie Jones	-	Hamilton	1975	flamesnracin3829@yahoo.com

First and last name	Title	City and country	Institution	Preferred email address to use
Susana Saramago	-	Portugal	-	Susysaramago@gmail.com
Denver Chapman	Ms.	Columbia USA	HUMANA MILITARY	denrnchapman@gmail.com
Kirk Brazeau	Father/Caregiver to Archer Brazeau	Toledo, Ohio - United States	We are part of the specialized EB clinic network here in the US - with our main care facility being the University of Minnesota Childrens Hospital EB clinic	Kirkbrazeau@gmail.com
Lissa Fogel	-	Tucson	-	lissafogel@gmail.com
Brayce Campagna	-	Gilbert, AZ	-	Brayce_89@yahoo.com
Simone Baird	Ms	Melbourne, Australia	EB family / DEBRA Australia	simonebaird@bigpond.com
Toni Roberts	Miss	Cape Town, South Africa	University	roberts.toni26@gmail.com
ANGELIQUE SAUVESTRE	HEAD OF DEVELOPMENT OF DEBRA FRANCE	LYON FRANCE	DEBRA FRANCE	angelique.sauvestre@debra.fr
