# Supplementary material for: Consensus-based guidelines for the provision of palliative and end-of-life care for people living with epidermolysis bullosa
Source: Orphanet J Rare Dis. 2023 Sep 4;18:268. doi: 10.1186/s13023-023-02870-8 (PMC10476410; doi:10.1186/s13023-023-02870-8)
Supplement: Supplementary file 2 — Additional file 2: EB palliative care clinical survey. [file 13023_2023_2870_MOESM2_ESM.pdf]

EB Palliative care: Clinical Survey

1. Which of the following best describes you?

Number of responses: 35

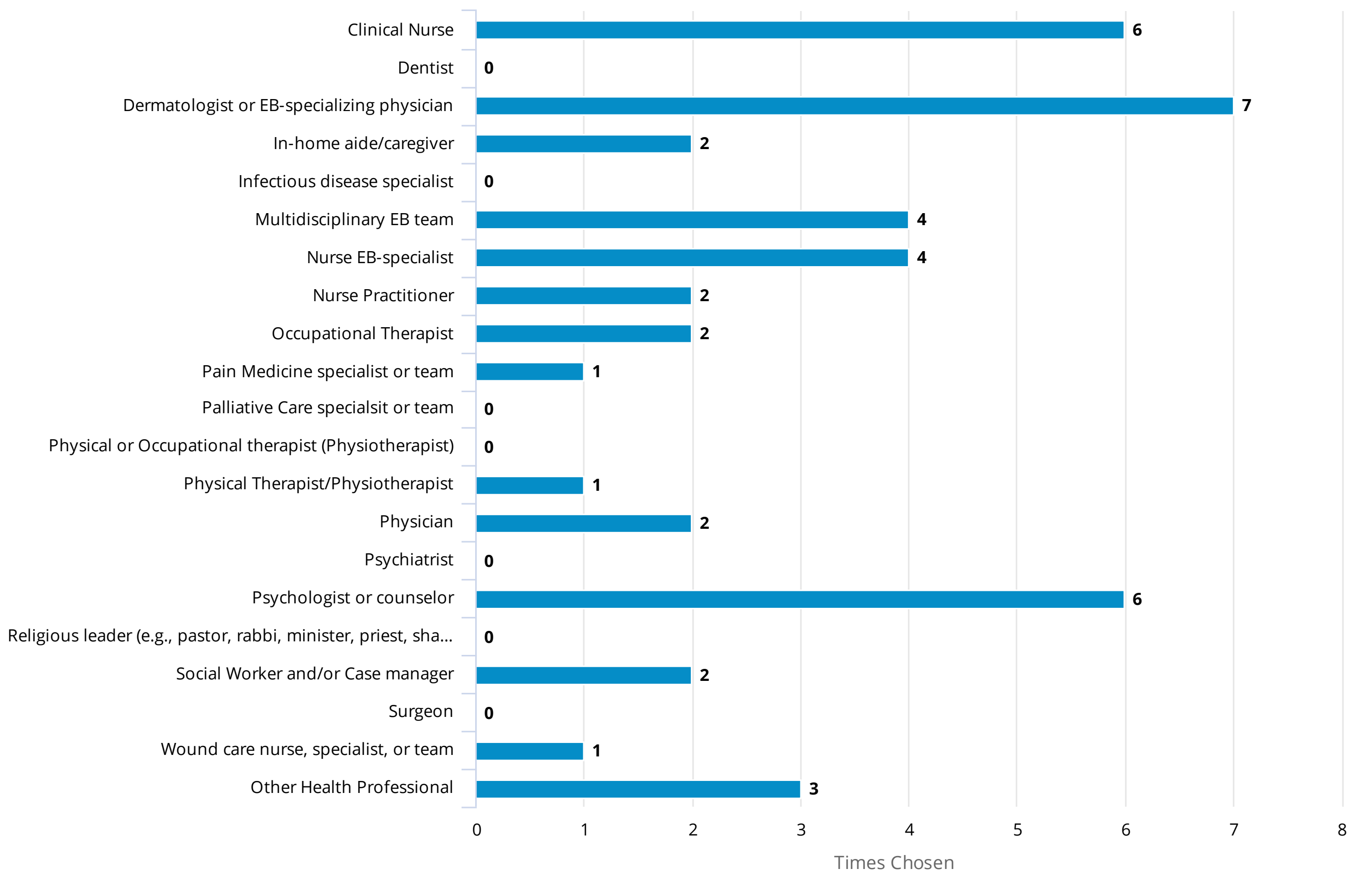

"Other Health Professional" text answers:

Mother of EB patient

Registered Dietitian

Pharmacist

If you have a specialty area of practice, please list it here.

Number of responses: 19

Text answers:

Dermatology -EB -Pediatrics

Pediatric Dermatology

Pediatric/Adolescent Dermatology (I do see adults with EB, however)

pediatric dermatology

Peds derm

Pediatric Dermatology

Volunteer Nurse Debra Brasil

NICU

Wound care and Palliative Care

Hand therapy

Pediatric GI physician

Clinical Psychology - Pain

pain

pediatric psychology consultation liaison

Pediatric critical care

Wound care and palliative care

pediatric dermatology

Dermatology-EB

Pain Management, EB

2. How many years of EB experience do you have?

Number of responses: 35

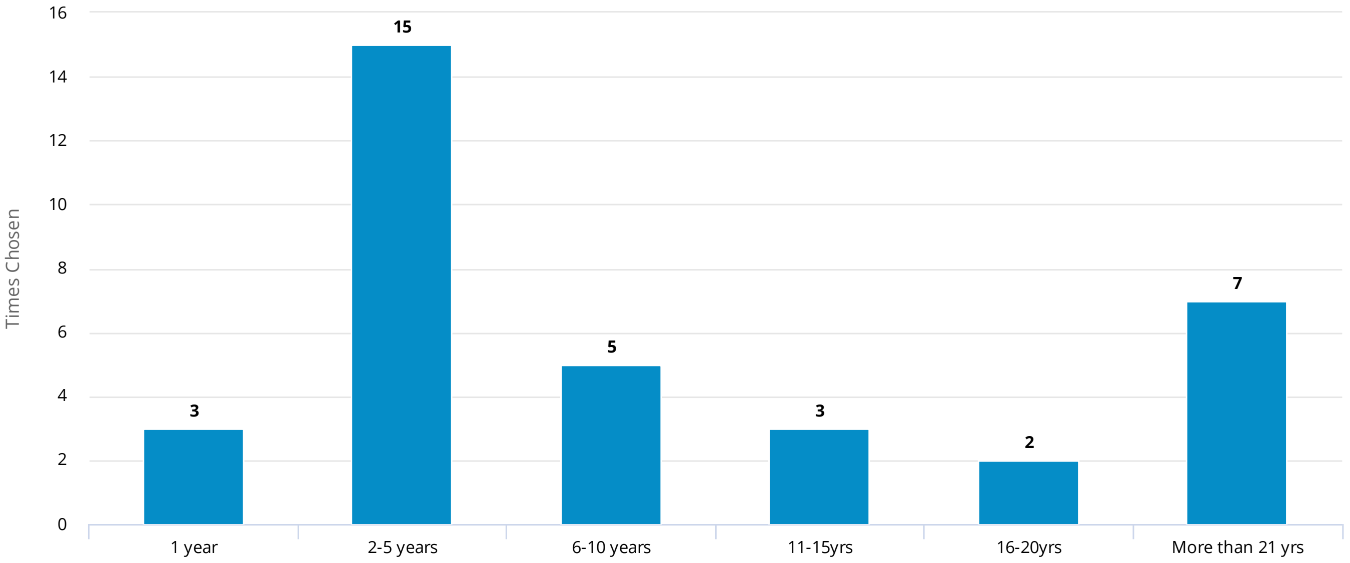

3. Based on the image below, please select the area in which you practice.

Number of responses: 35

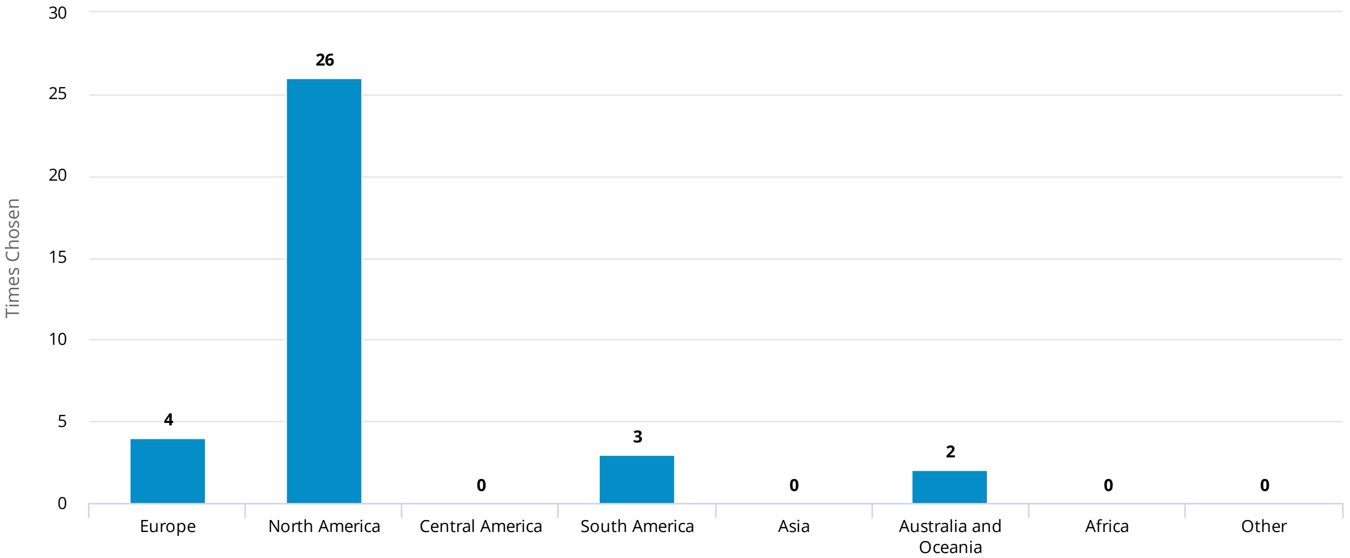

What city and country do you live in?

Number of responses: 33

Text answers:

Cincinnati Ohio USA

Montreal, Canada

Phoenix az usa

Austin, TX USA

Acton, Massachusetts, United States

New York, usa

Chicago

UK

Blumenau - Brazil

Grand Junction, CO. USA

CIncinnati, Ohio

Barnegat USA

Atlanta, GA USA

Wellington, New Zealand

Foster City, California/USA

Philadelphia, USA

Philadelphia, PA

Philadelphia, Pennslyvania

Philadelphia, PA USA

Philadelphia, USA

Brazil

Gulf Shores USA

Cincinnati, OH

Phoenix, US

ann arbor mi usa

gfgfkv

Phoenix, USA

Wellington, New Zealand

phoenix, USA

Chandler, AZ

Argentina

Barcelona, Spain

Phoenix, Arizona USA

4. Please select your Religious/Cultural Affiliation:

Number of responses: 32

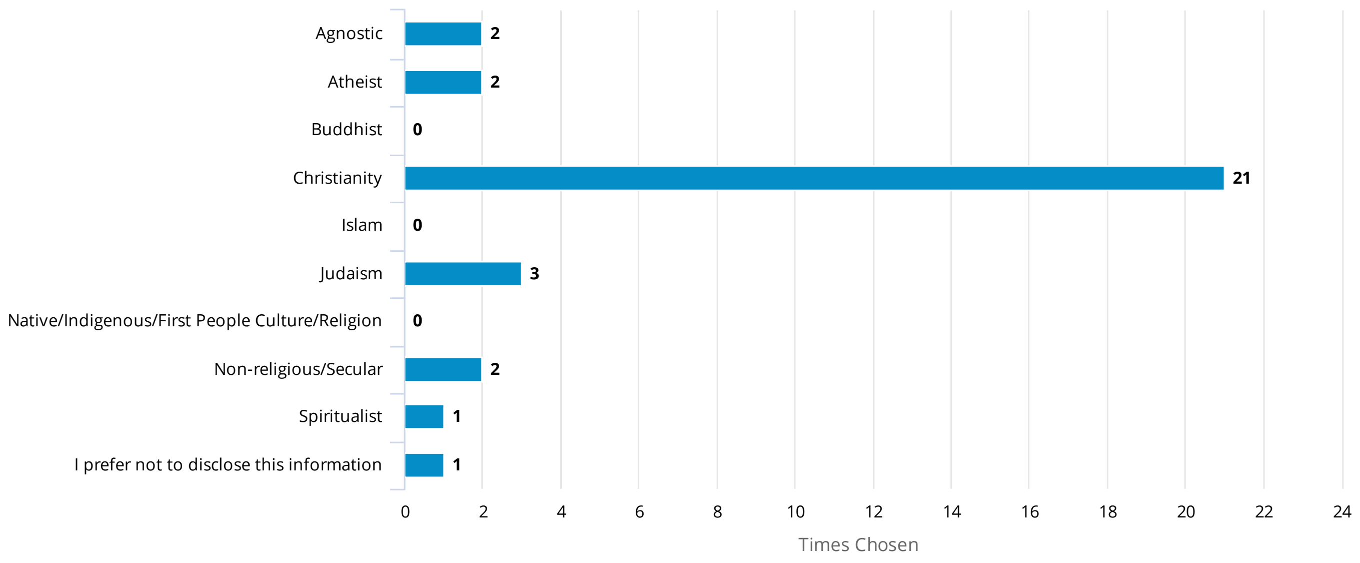

What is your level of participation with your religious/cultural affiliation noted above?

Number of responses: 32

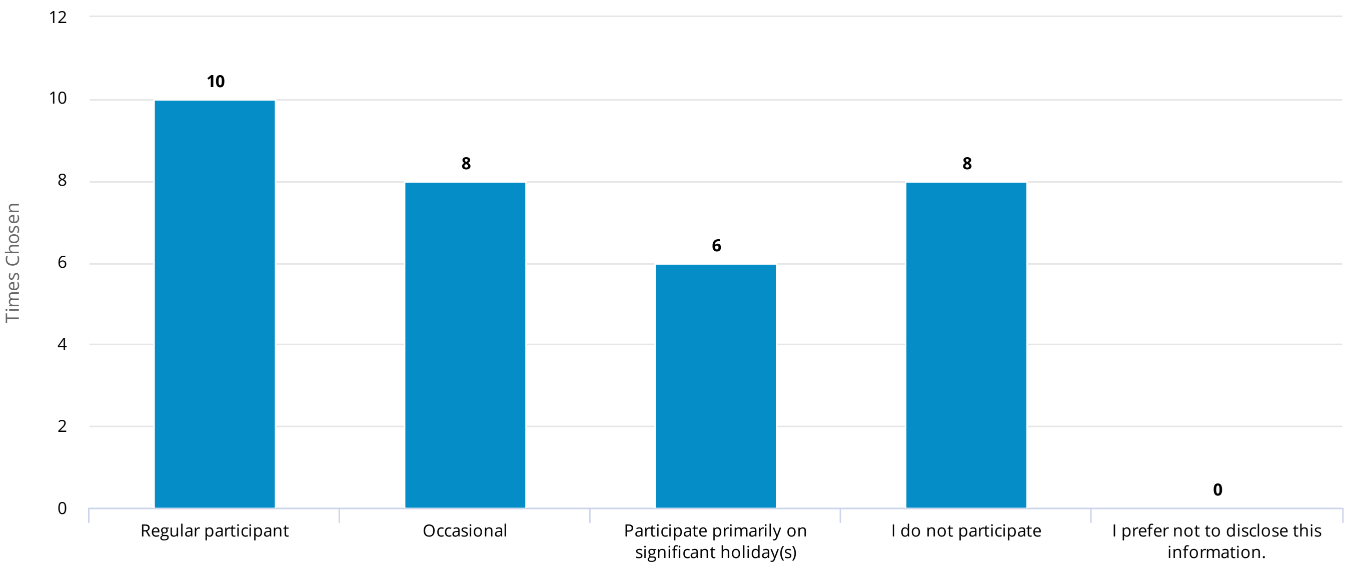

5. Self-identified gender of respondent?

Number of responses: 34

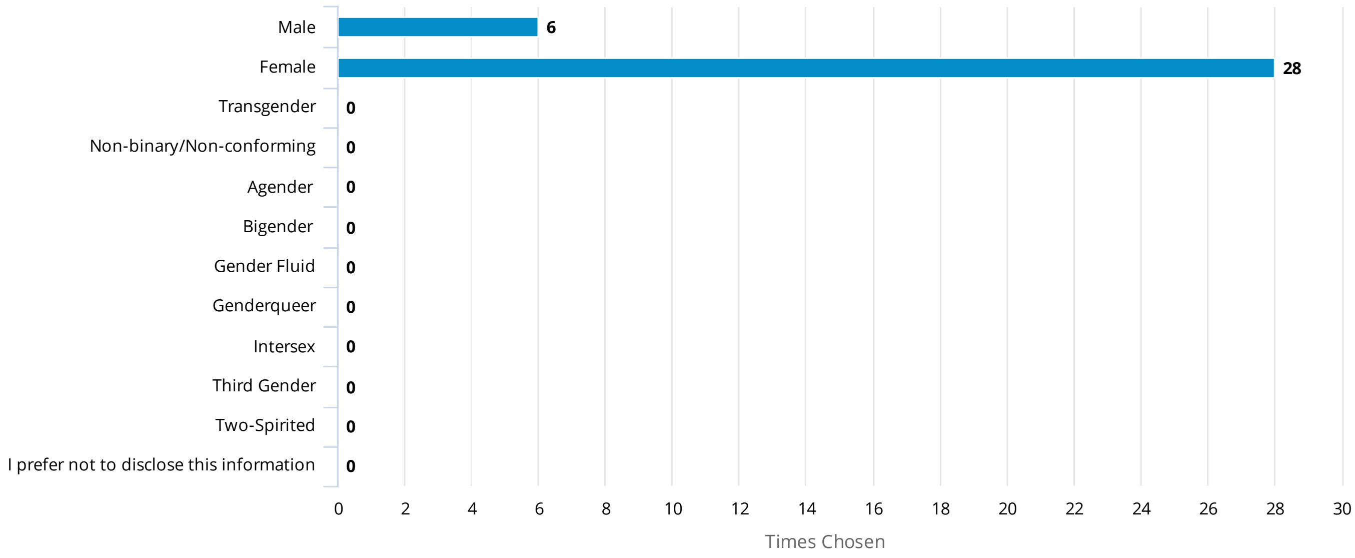

1. Which type(s) of EB do you see in your caseload?

Number of responses: 27

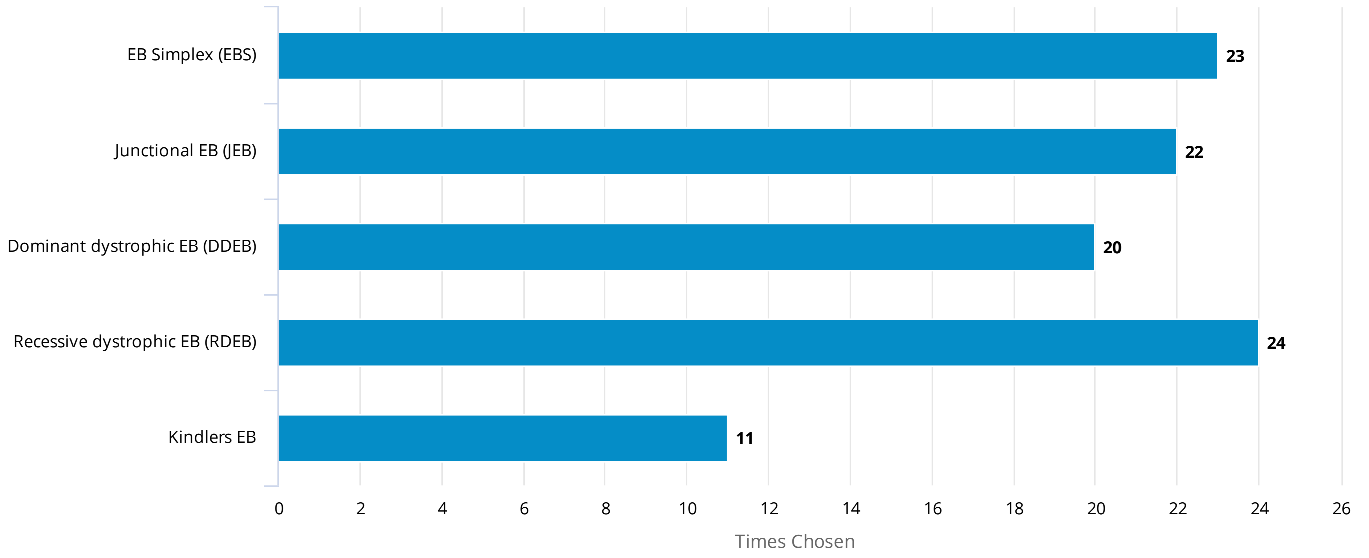

Please add any other details you think would be helpful. For example, do you cover children or adult care, how many people you see in a week or month, etc.:

Number of responses: 17

Text answers:

Children and adults. ~ 10/mo

Child-adult. Monthly clinic + ongoing treatment

numbers vary greatly; usually see pts at 3-6 month intervals depending on severity, problems, etc; I see both children and adults

I see primarily children but have some adults in my EB practice even though I don't see adults otherwise. I see about 10 EB patients per month.

Take care of ~75 with EB of different types; am regional expert
Adults and children - usually see 1-2 with every clinic

Acting with the multidisciplinary team in situations of imminent death, in the comfort of palliative states

In our NICU we see newly born, newly diagnosed EB patients. We have 3-4 patients a year,

RDEB Adult - once a week
Mainly adults. Have had a few new born babies.

I work in a children's hospital where we have a monthly EB clinic. We see Childrens from birth to young adult and occasionally see mature adults. Our clinic is multidisciplinary. I do not see that many patients regularly outside of the clinic as most come from a long distance away.

Pediatric multi-disciplinary EB clinic at a children's hospital. We see about 4 patients every 2 months.

Part of Multi-D team at Children's Hospital of Philadelphia (CHOP) which began in December 2017. Clinic occurs approximately 6-8 weeks where we typically see 4-5 patients during a 5 hour visit. Ages: newborn - 20 yrs old

Adult

Very rarely see EB, maybe 1-2 per year

We see EB of all ages. We generally have 3 patients in the multidisciplinary clinic once per month and on occasion see 2-4 patients in the clinic outside of EB clinic for urgent issues.

children and adult care, 4 patients in a week

We are a children hospital and we see 2 or 3 EB patients in a week

I work with all ages.
Our team sees 3/month in clinic and I see patients with EB when they are admitted to the hospital.

2. Select all healthcare or support professionals that you currently utilize in your practice with patients who have EB. (check all that apply)

Number of responses: 27

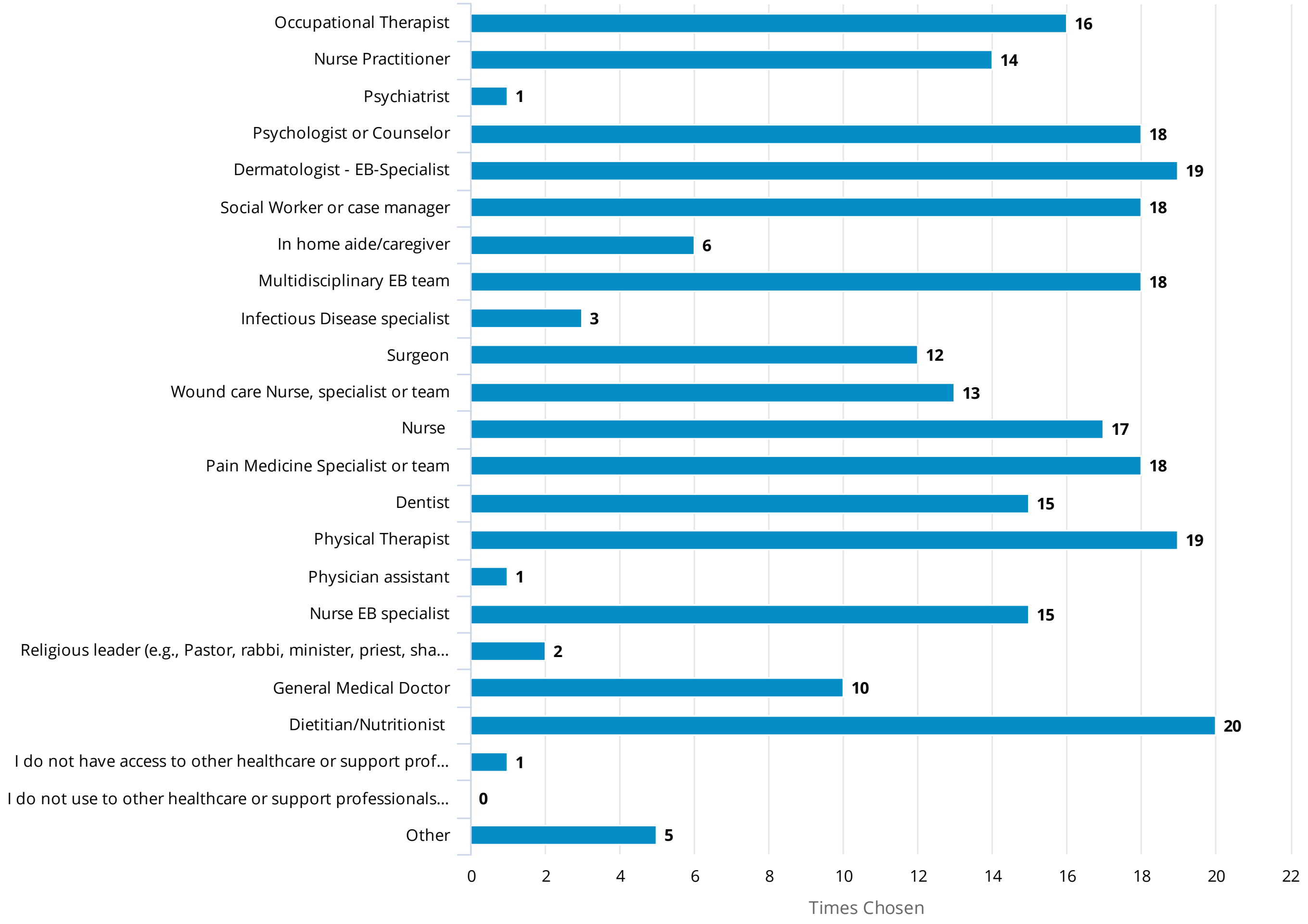

"Other" text answers:

rte
I am the derm/EB-specialist; who is utilized depends on type of EB and problems encountered
ophthalomologist
orthopedist
GI specialist

3. Which team members/resources do you believe should be provided, at a MINIMUM, based on the diagnosis of EB alone?

Number of responses: 27

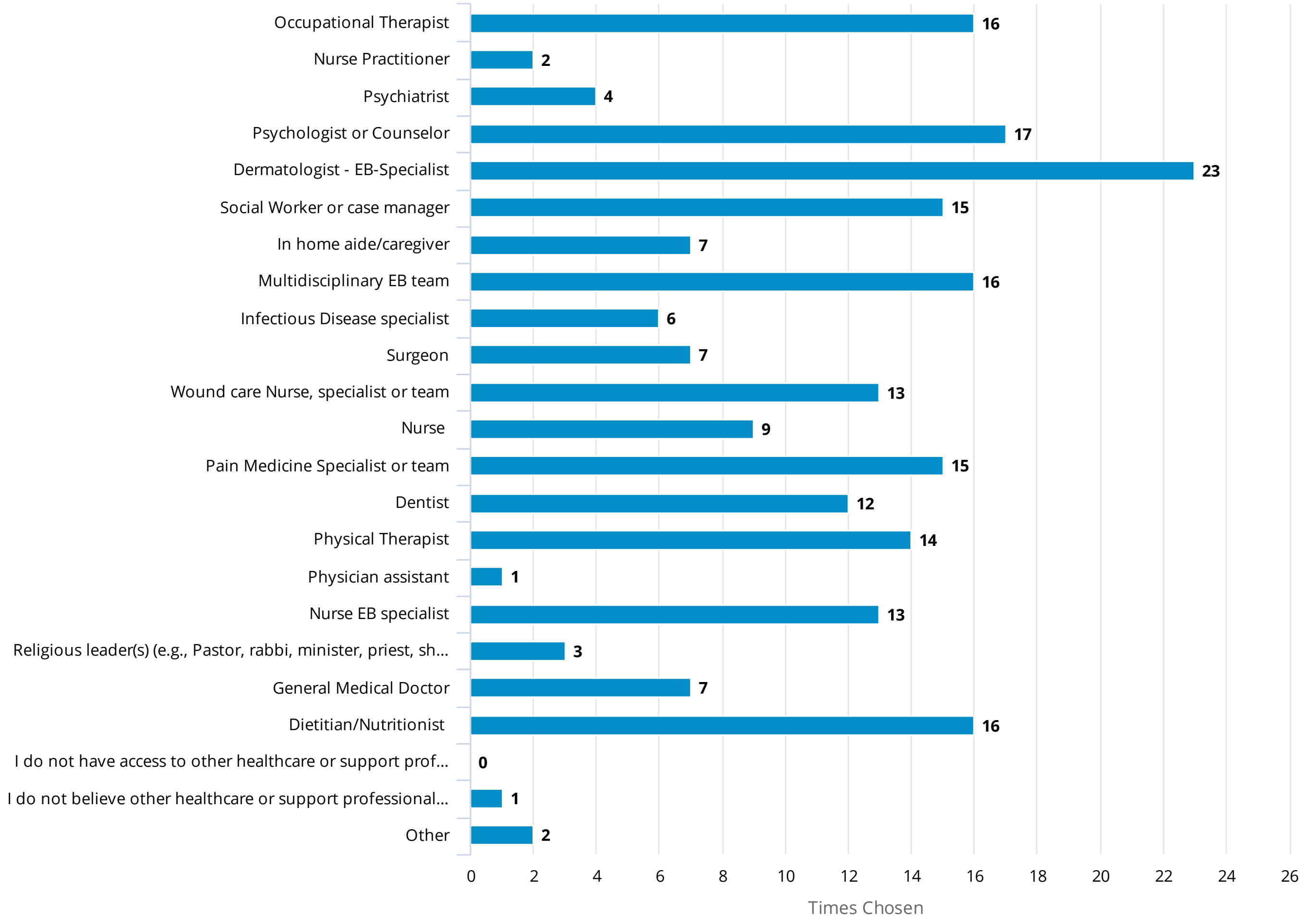

"Other" text answers:

GI Specialist

Pharmacist

4. Which team members/resources do you believe should be provided, IDEALLY, based on the diagnosis of EB alone?

Number of responses: 27

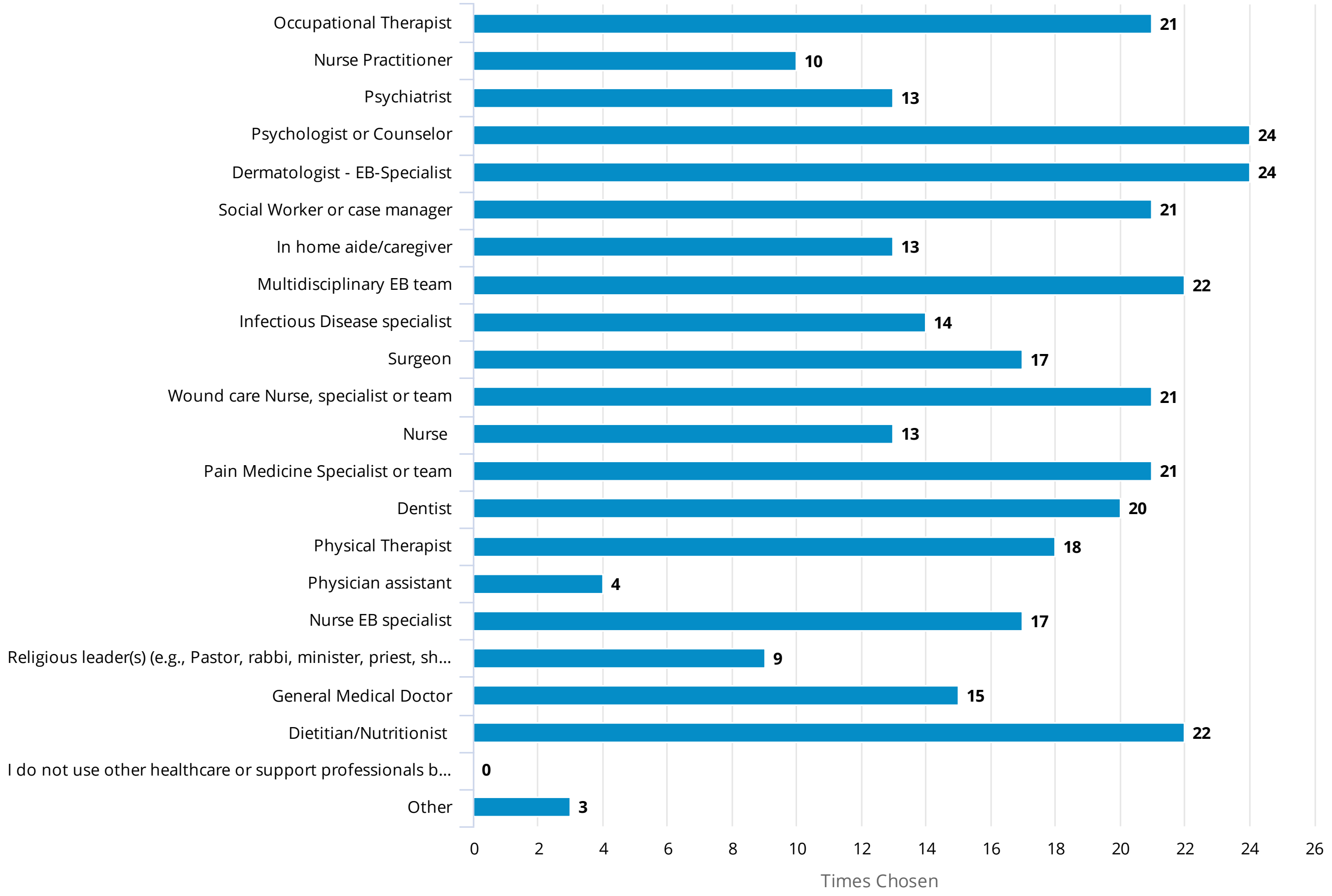

"Other" text answers:

GI specialist

Pharmacist

patient support group/palliative care

5. What triggers/indicators do you believe should initiate the introduction of YOUR AREA OF PROFESSIONAL EXPERTISE? (please name your area of expertise in your response below)

Number of responses: 25

Text answers:

All of our patients work with nursing, either in person, email or by phone. Care Management needs, wound care experitse and guidance, education - RN EB Derm

diagnosis and management of EB

gfd

Inability to identify or a access daily activities at desired level of performance.

Dermatology; when the diagnosis is made

dermatology - just the diagnosis of EB

Pediatric dermatologist

Wounds

all pateints with EB should be given the opportunity to access a specialist EB service

The presence of the nurse in the team makes a difference in various disease processes. Monitoring the development and outcome of the difficulty brings knowledge and brings the team closer to the difficult time of this process.

In home EB wound care

NICU nurse. Babies come to us for care from their birth hospitals. We treat the baby, support and educate the family.

EB diagnosis - EB clinincal Nurse

Children with developmental concerns, children and adults with limitations in self care and access to community resources and engagement.

one visit based on diagnosis to screen, then f/u as needed

Pediatric GI -- growth issues, dysphagia, reflux, constipation, abdominal pain

Psychologist

Co morbidity, complications

dhshfgjh

All patients with EB

active skin disease, need for diagnosis or work-up

EB nurse- initiation should begin at time of diagnosis

Dermatologist

Social Work - being the link between the team in the hospital or health center and all the other systems like school or welfare state to provide the best life quality to the patient and the family or carers

Pain Psychologist - At time of diagnosis

6. Are you currently a member of the Palliative Care CPG Panel?

Number of responses: 21

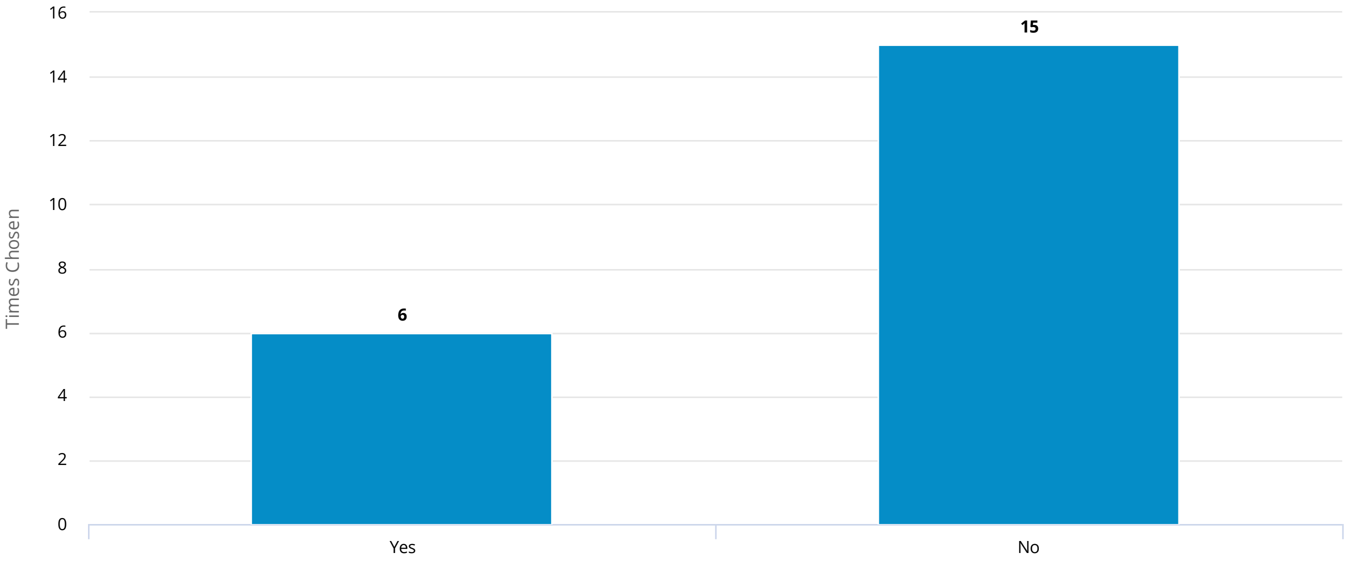

1. Prior to completing this survey, were you aware that Palliative Care emcompasses the care provided to a patient during any chronic life-limiting/threatening illness, that it is not only limited to treatment at the end-of-life, and that it differs from hospice care?

Number of responses: 16

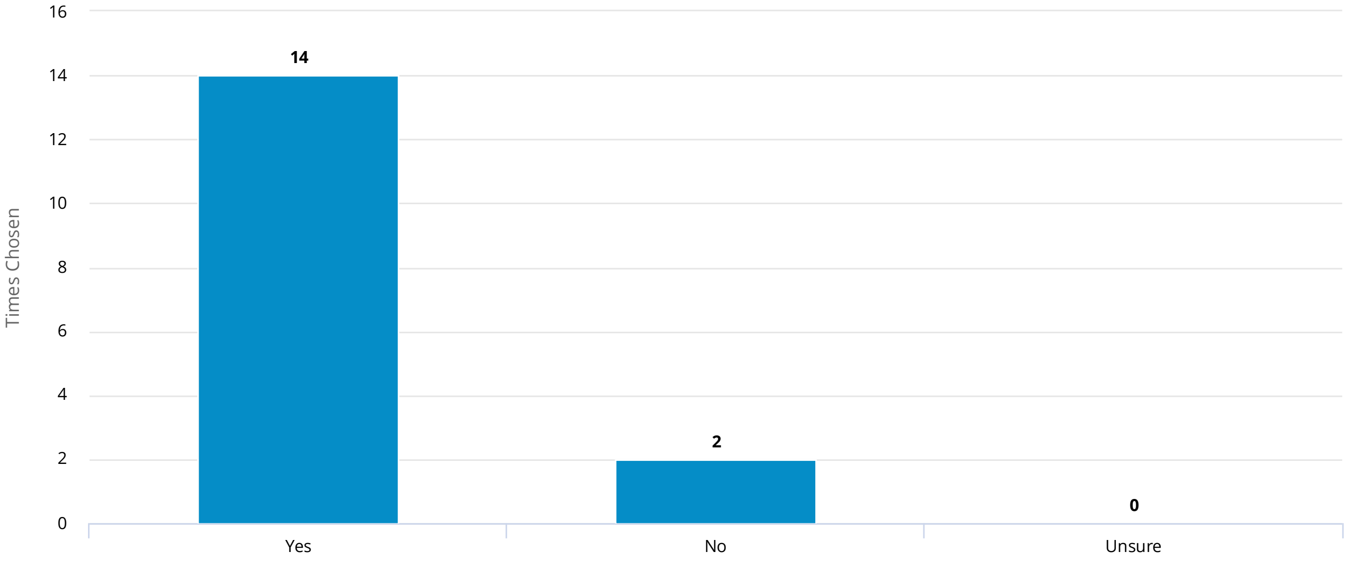

If you have any additional comments or thoughts, please write them here.

Number of responses: 3

Text answers:

We should be introducing Palliative care to our families earlier so they understand the concept and differences between Hospice and palliative Care

have used such for many years; VERY helpful though we need to provide education to them (on occasion)

I am interested in participating on the panel and learning about this specialty.

2. When do you believe is the appropriate time to begin the referral process to Palliative Care when dealing with EB?

Number of responses: 16

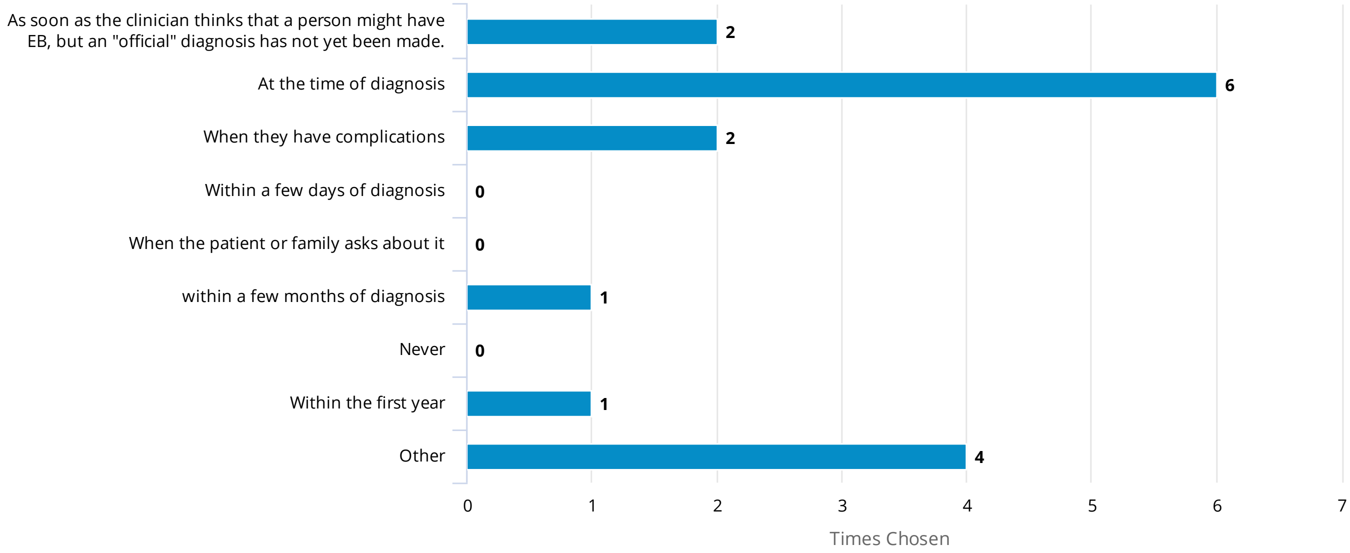

"Other" text answers:

only allowed one choice, complications 1st choice

again, for me, this depends on the type and severity/duration of problems BUT BEFORE individuals are at end of life!

End of life apparent

when the clinical team deems appropriate

3. What triggers you to make or suggest a referral to Palliative Care?

Number of responses: 16

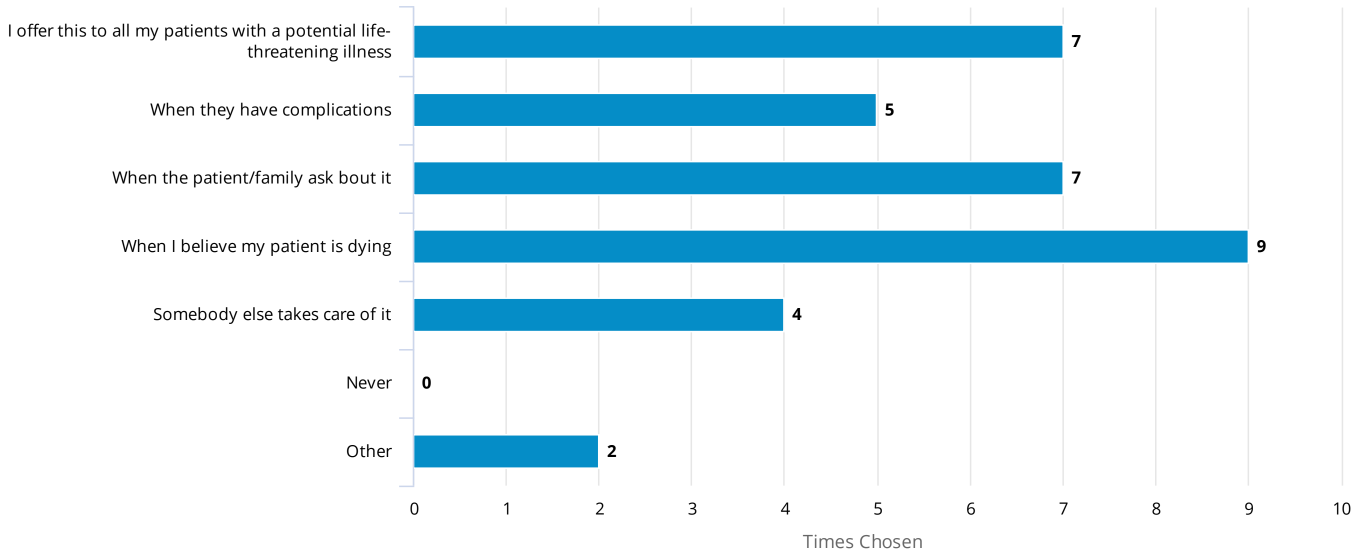

"Other" text answers:

before, as noted above, death is imminent

We should offer it to all patients with this chronic illness - in practice, not sure if we have a specific protocol/timeline

4. Who provides Palliative Care at your institution?

Number of responses: 16

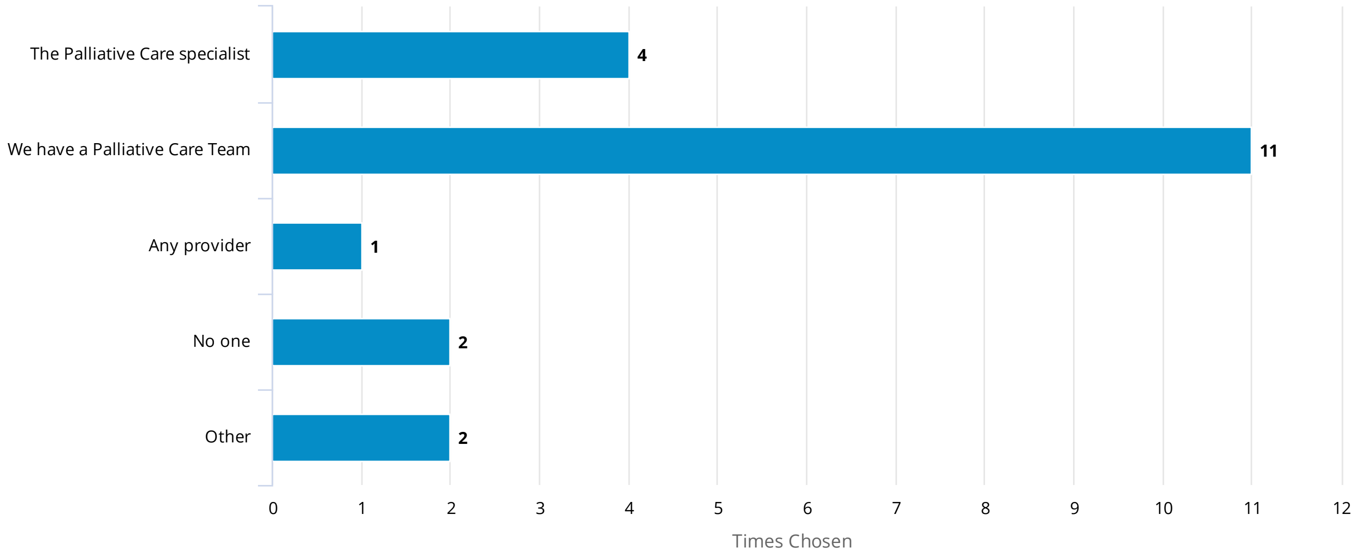

"Other" text answers:

We have a both hospital and community palliative care plus the hospice. Community Care works both with or independantly of the hospice.

not sure

5. What type of educational resources do you provide regarding Palliative Care?

Number of responses: 16

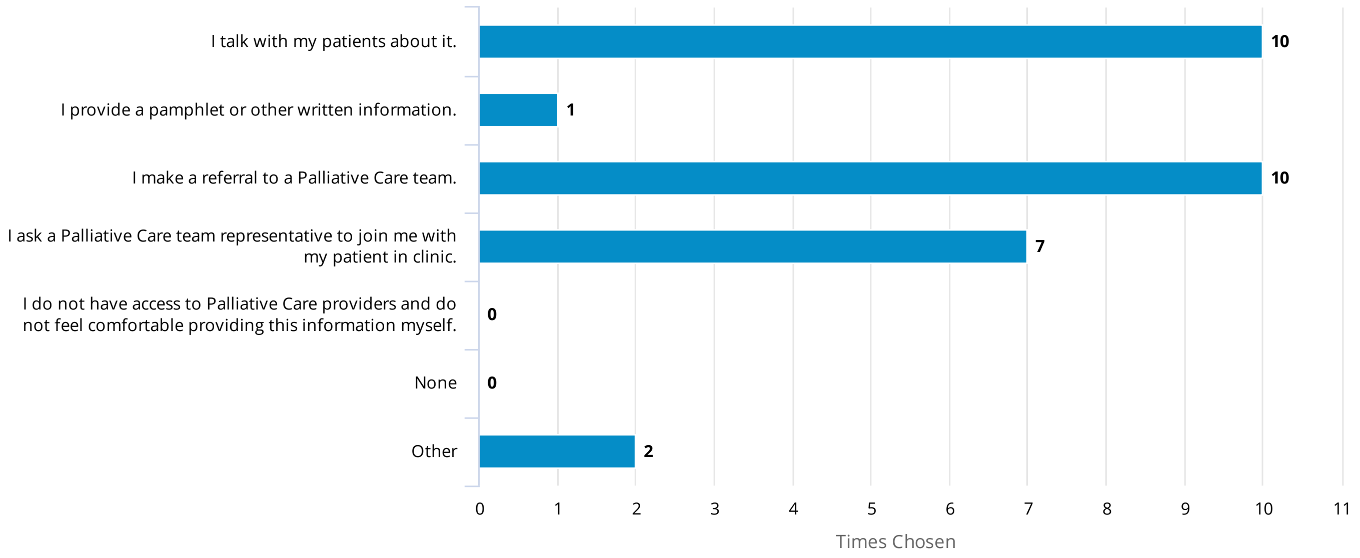

"Other" text answers:

not sure
Usually discuss with patients/families after they have met palliative care team

6. Did you know that all management of EB is palliative?

Number of responses: 16

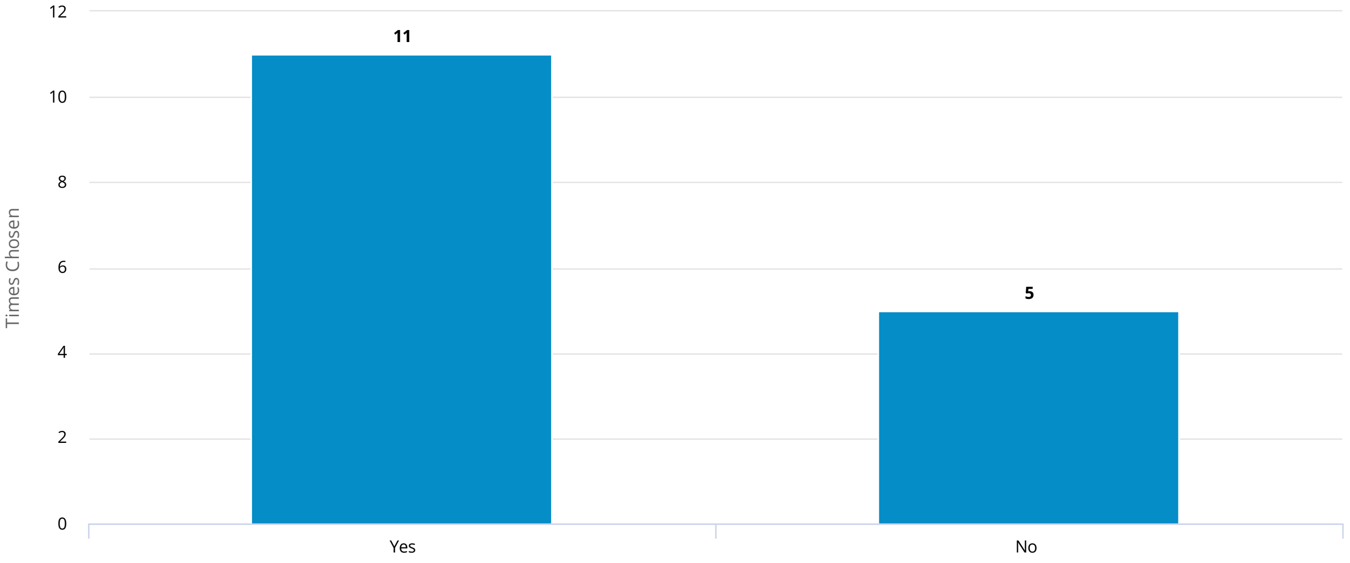

7. Do you tell that to your patients when introducing Palliatice Care in the conversation?

Number of responses: 11

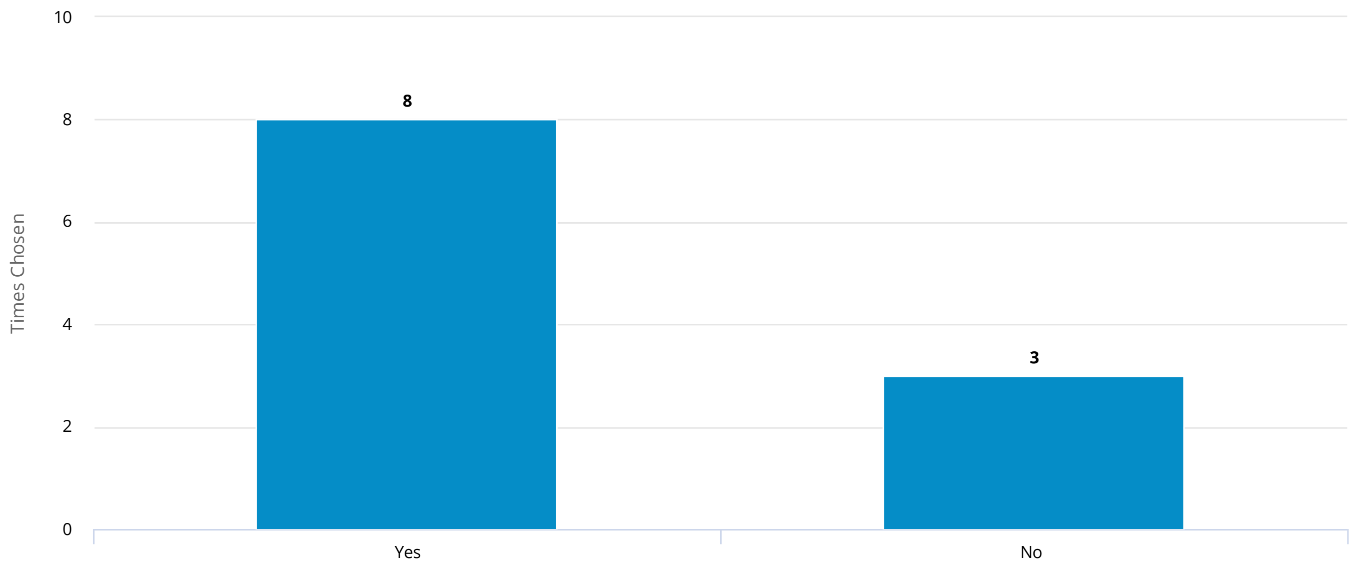

8. What do you tell your patients with EB (if anything) about the role of Palliative Care in their overall treatment plan? Please elaborate

Number of responses: 15

Text answers:

Agree that the palliative team should be involved early, and pre-crisis.

again, when the frequency and severity of disease is impacting (appropriate) decision making in the care of the individual and the family

I refer to the team and let them explain. I tell them that they can help with quality of life and in making important decisions.

we are aware as a team that we need to involve palliative care sooner in the patients journey and have been looking at ways to address this

The chronic characteristic of EB makes the palliative clinical practice gradually introduced in the care phase. The family and patient need to understand the benefits of joining the palliative team as ease in the care process.

I tell them nothing. I deal with what is in front of me.

I have not addressed this

It's not in my practice flow (but should be -- or if not mine specifically, our team should be discussing this with families regularly)

Unless initiated by a patient/family, these conversations are typically initiated and discussed with primary medical team/attendings

Our goal is to provide comfort and hopefully prevent infections

shfdhdfh

that the team helps address dealing with a life-limiting or life altering chronic disease, both support to family and patient

I don't specifically head this conversation as this is the role of the physician

We try to explain that all treatments for EB are palliative given tath there is no cure, but that doesn't mean that we are not going to stop treating them, we are going to be by their side and work to provide them the best life quality posible depending on their needs

I explain that Palliative Care helps make whatever life is left, whether it is 5 minutes or 50 years or longer, and help make that life worth living.

9. What do you fear or worry about with respect to referring a patient to Palliative Care?

Number of responses: 15

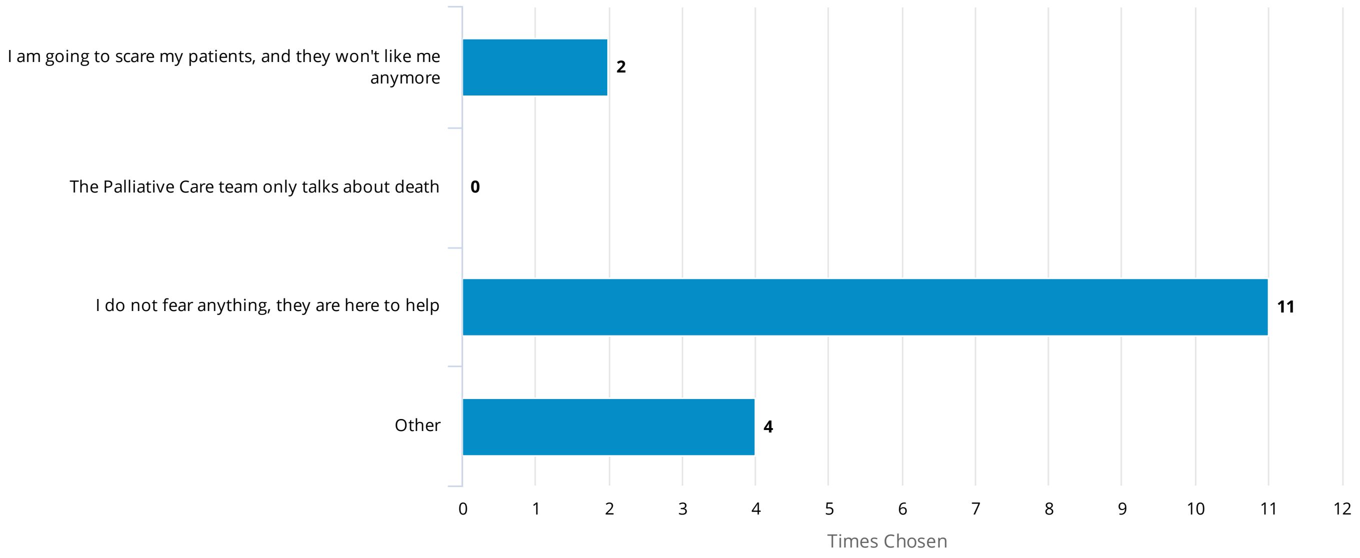

"Other" text answers:

That they will misunderstand what palliative care is.

its hard for my patients to grasp the concept

I don't want patients to think that "we think they are dying soon"

That my patients won't accept the help becasue of their misperceptions.

10. What symptoms/issues have you found to be the most difficult to manage on a day-to-day basis?

Number of responses: 16

Rank	Choice	Distribution	Score	Times Ranked
1.	Pain		171	16
2.	Wound management		162	16
3.	Itch		148	16
4.	Family stressors		141	16
5.	Mental Health concerns (e.g., depression, anxiety, etc.)		137	16
6.	Infections		120	16
7.	Access to proper health care		116	16
8.	Feeding		108	16
9.	Access to medications		87	16
10.	Access to supplies		82	16
11.	Hydration		81	16
12.	Breathing		56	16
13.	Catastrophic bleeding		47	16

Lowest — Highest

11. What symptoms/issues have you found to be the most difficult to manage at the end-of-life?

Number of responses: 15

Rank	Choice	Distribution	Score	Times Ranked
1.	Pain		168	15
2.	Family stressors		146	15
3.	Mental Health concerns (e.g., depression, anxiety, etc.)		134	15
4.	Wound management		121	15
5.	Infections		104	15
6.	Itch		102	15
7.	Feeding		100	15
8.	Access to medications		95	15
9.	Access to proper health care		93	15
10.	Hydration		87	15
11.	Breathing		80	15
12.	Access to supplies		75	15
13.	Catastrophic bleeding		60	15

Lowest — Highest

12. Did you find any other challenging symptom we did not mention above? Please share your experience(s) with us here:

Number of responses: 15

Text answers:

Changes such as fluid retention, bloating of abdomen with only lab changes noted in albumin which is an ongoing concern. Difficult to explain to families what these symptoms are from.

non

again... so much of this depends on the type of EB... but generally, these issues related to dystrophic or junctional cases

I have not had any patients at end of life other than very young babies with severe junctional EB.

no

dysphagia

No

No

School attendance

Lack of primary care

vbmxvmvzn

none

From a clinician perspective, at end of life, it is hard to keep the balance between "saving your patient" and strictly comfort measures.

Discrimination, social isolation, cultural and spiritual aspects

no

13. How could EB symptom management education be better delivered to children, their families, and healthcare professionals (including those that are not part of an EB team)?

Number of responses: 16

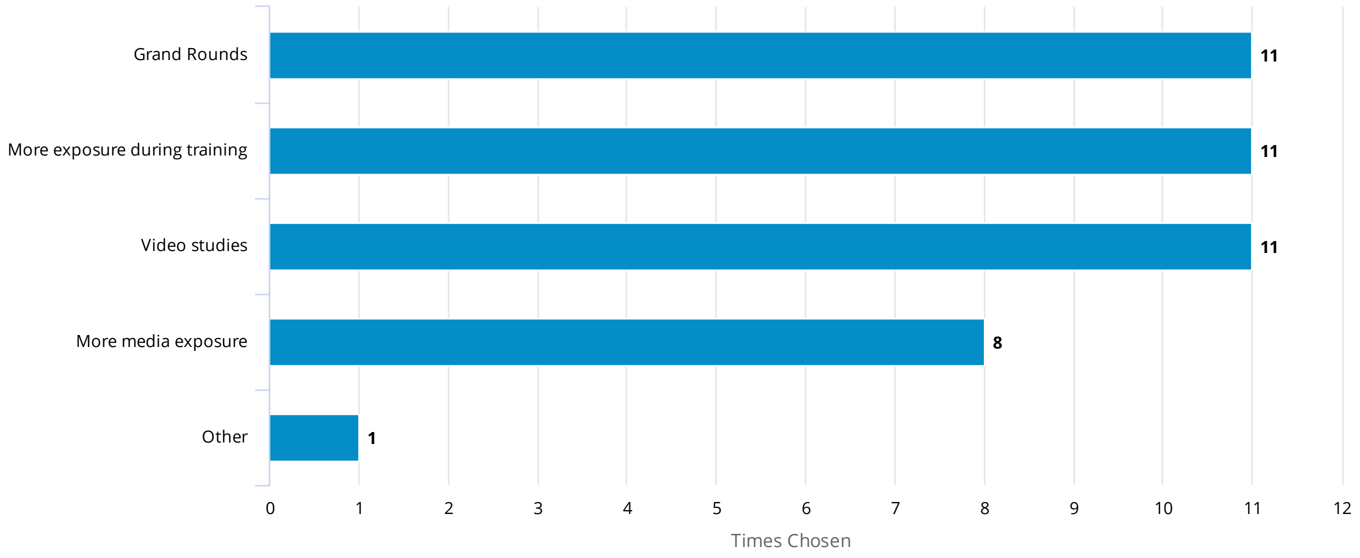

"Other" text answers:

Age appropriate education

14. What support systems do you recommend to your patients to help them cope with EB?

Number of responses: 16

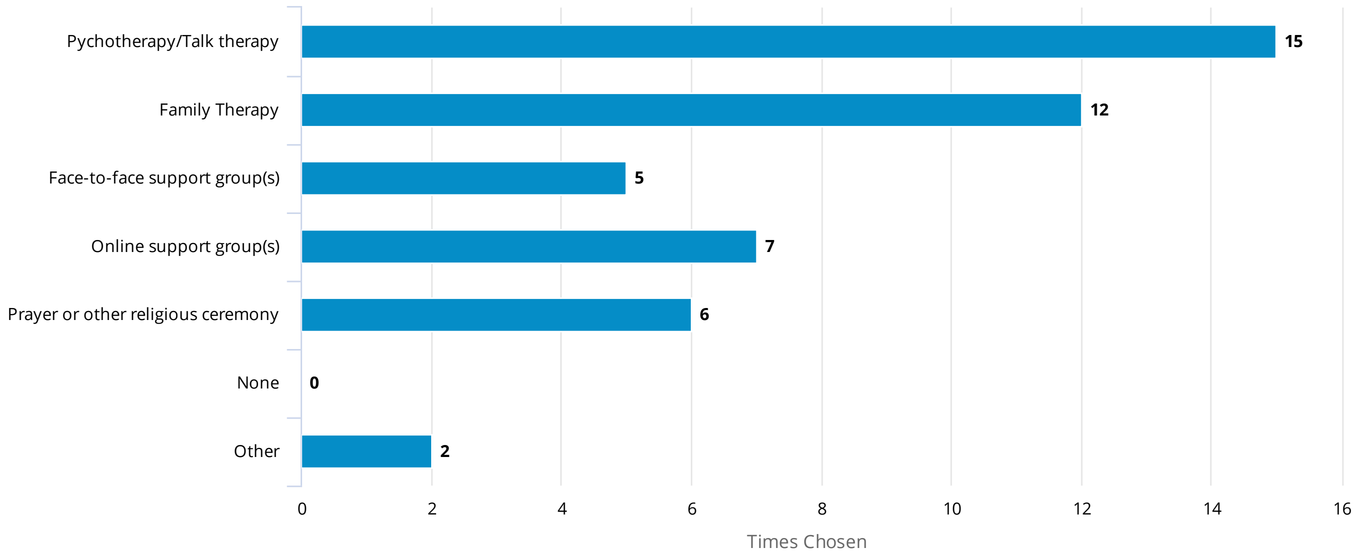

"Other" text answers:

engagement in outside activities with others
meet other patients and families

15. Are you aware of any support groups that your patients with EB utilize?

Number of responses: 16

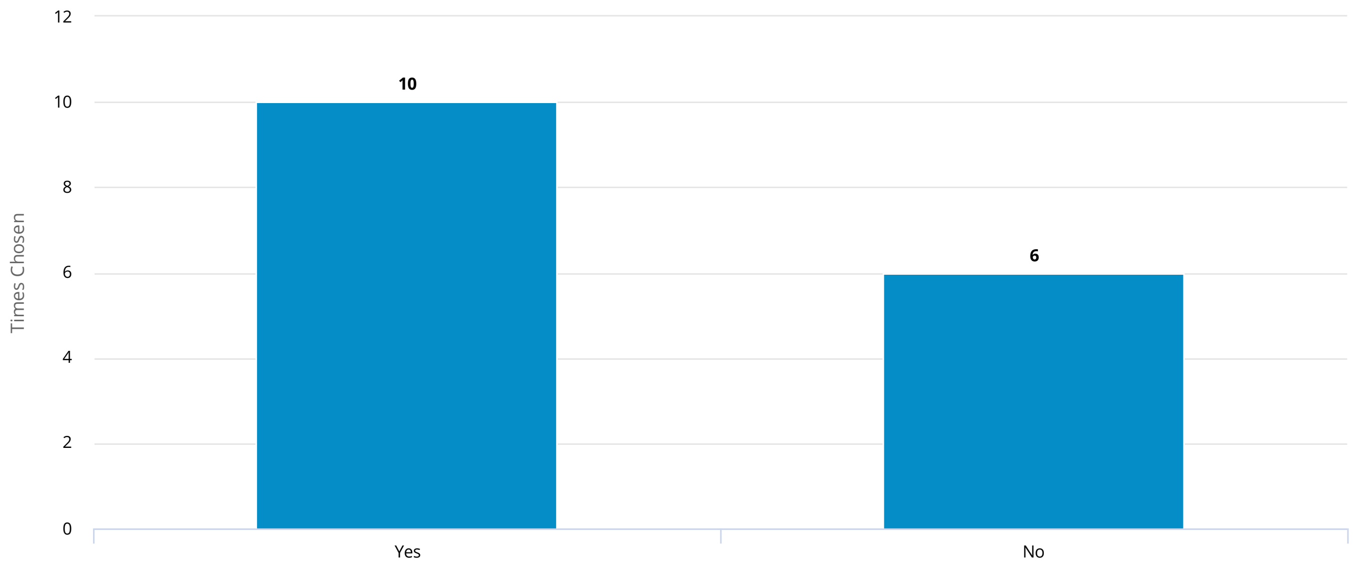

16. Select all types of support groups you have recommended to your patients who have EB?

Number of responses: 10

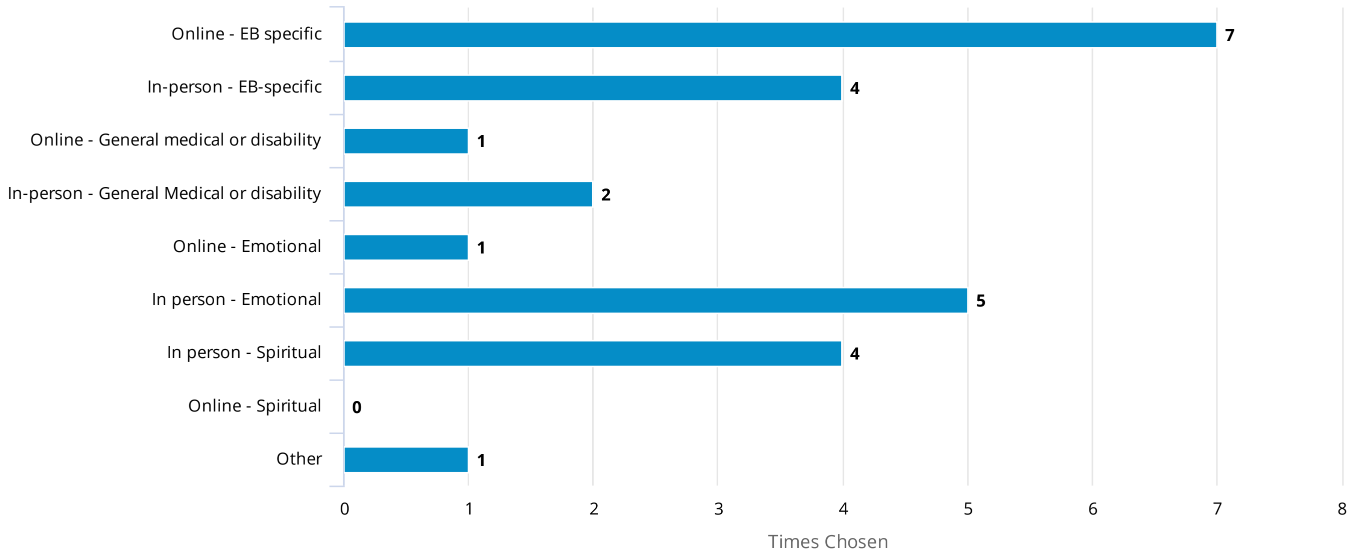

"Other" text answers:

Other

17. What do you see as the emotional needs of a person living with EB who recieves Palliative Care during:

Number of responses: 15

Infancy	Toddler years	Elementary/Primary/Grade school years	High/Secondary school age	Young adulthood	Middle adulthood	Older adults
Emotional Psychosocial support for parents, pain management for child	Emotional Psychosocial support for parents, pain management for child, alternate methods to manage pain and itch - distraction, etc	Emotional support to family and child with unexpected illness/disease - grieving, pain. Support child as their disease progresses and changes in relationships with peers which ultimately occur, pain, alternative measures for comfort	Emotional support family and child with chronic illeness, management of pain and changes in peer relationships, alternate methods to manage pain, preparation for future with this disease	Emotional support to young adult with chronic illness, preparation for future, discussion regarding disease progressions, etc	Emotional support, pain management,	Emotional support, suppot in life changes
parents in all stages but especially this one	5 stages: refuse, anger, sadness, acceptance	5 stages: refuse, anger, sadness, acceptance	5 stages: refuse, anger, sadness, acceptance	5 stages: refuse, anger, sadness, acceptance	5 stages: refuse, anger, sadness, acceptance	5 stages: refuse, anger, sadness, acceptance
family support	patient and family support	above plus education of other students, teachers, counselors	as above	patient/family support; education of those in work environment...	as above	as above
family support	family support	school issues/adaptation	peer issues	job assistance	jobs and family	family concerns

Infancy	Toddler years	Elementary/Primary/Grade school years	High/Secondary school age	Young adulthood	Middle adulthood	Older adults
na	na	na	na	recognition of changes in their self and impact on EB	Living well with EB, relationships, having children	Living well with EB
Emotional support to family	Assistance in understanding the disease process and importance of specialized monitoring	School inclusion within the limitations of the disease phase.	School inclusion within the limitations of the disease phase.	Management of adversities at the stage of life, family composition instructions, and awareness of the importance of palliative care for a better understanding of the disease stage	Prepare family and patient for the disease progression process associated with hormonal and body changes. Psychological support.	Prepare family and patient for the aging process, body changes and physical changes and limitations due to EB
Unsure	Unsure	Counselling, peer support	Counselling, peer support	As above	As above	As above
Family support	Family support	Support of family and child as they transition to interacting with others	Support of child with social stressors and engagement in activities that they find fulfilling	support of young adult to engage with others and in activities that they find fulfilling. Support of person to gain more independence.	Support of adult in being independent as much as possible	Support of adult in maintaining independence and outside activities.
care & comfort	age appropriate behavioral challenges and how to manage them	social isolation, anxiety, depression	anxiety, social isolation, depression	anxiety, transition to adulthood (e.g., college), independence	N/A	N/A
None	None	None	None	None	None	Support
gfggdz	\dhdgh\dgh	\dgd\ngd	\dndngd\n	\nd\gngd	\ng\dn	\ngd\ngd\
not sure	connecting with loved ones, having experiences	fitting in with peers, connecting with loved ones	fitting in with peers, having relationships and a purpose	fitting in with peers, hvaing relationships and a purpose	not sure	not sure
Parent/caregiver respite or support	Parent/caregiver respite or support	School/peer support and education, child feels and looks different from peers	depression/anxiety, missing school, eating issues	can they attend college or get a job?	Dating, jobs, peers, relationships	depression, losing hope

Infancy	Toddler years	Elementary/Primary/Grade school years	High/Secondary school age	Young adulthood	Middle adulthood	Older adults
Fear of suffering pain, feel limitated compared to others, the relation woth your family and equals	Fear of suffering pain, feel limitated compared to others, the relation woth your family and equals	Feeling different, discrimination and isolated. Thouths about what's wrong with them and why they have to be this way and suffering physical and emotional pain	Feeling different, discrimination and isolated. Thouths about what's wrong with them and why they have to be this way and sexual issues	Feeling different, discrimination and isolated. Thouths about what's wrong with them and why they have to be this way, sexual and working issues	Feeling different, discrimination and isolated. Thouths about what's wrong with them and why they have to be this way, sexual and working issues	Feeling different, discrimination and isolated. Thouths about what's wrong with them and why they have to be this way, sexual and working issues
Family support	Family support, helping person accept their differences	helping person accept their differences, dealing with bullies, friendship issues	helping person accept their differences, dealing with bullies, transitioning self and care to adulthood, relationship issues	Transitioning to adulthood, feeling safe in search for providers, relationship issues	Relationship issues, concerns about having a family of their own, possibly end of life	Relationship issues, end of life, legacy building

18. How do you think does the expectation of a potentially shortened lifespan affects lifestyle development and values of a person with EB?

Number of responses: 15

Text answers:

It can create a feeling of doom and lack of motivation- no interest in planning an adult life, education or activities, relationships in some. Others who have as "normal" a lifestyle as possible seek to move forward despite their disease and continue with college, employment and marriage. Early childhood management of disease and limitations is critical.

value life, often good support from family

honestly, this varies in my experience by the individual and family dynamics... support from others

Huge impact

Immeasurable, ripple effect around the world as peers die.

The child with EB needs to be
It is prepared for a life with some limitations, but it will not significantly change its focus on society, its education, its insertion in the labor and social market nor its family constitution. if we can prepare them with this awareness, giving them opportunities for inclusion. If the child is not prepared, the patient's tendency to live under the view that life will be cyclic and full of limitations. early acceptance intervention and demonstration of good examples in the EB community is needed

They become very self focussed.

Some seem to wait to die. Others like to challenge themselves and take on new experiences. Some want to help others with EB.

increased anxiety

Depression

jnd\ngd\

absolutely does! delays transition discussions both physical transition and emotional/body transition by HCPs
goals are delayed or not sought, access is limited purposely, kept close by families without being able to live life fully

Patients might feel if life is shortened then why bother with going to school, getting a job, having friends or relationships. They might feel cheated from life.

It deppends on how ready is the society they live to provide them a carer system. In less developed care system people can be afraid of living with pain till the end and being able to access to basic wound healing products and of curse the sufering of the family or carers. Some times desperarion can make people or families try to move to another country to have the chance to have a better end of life

I have seen this run the gamut from patients/families becoming very religious or turning to their religion. For some, there is a sense of pressure to do all the things they want to do before they pass on. Others very much value family and deep/close friendships as well as having experiences over things.

19. What non-medicine strategies/skills have you found to be fundamental to promoting quality of life and self-determination through the lifespan of a person with EB?

Number of responses: 15

Text answers:

Encouragement to maintain and continue education, encouraging a "you can do anything" attitude, offering pt many experiences . Role models and mentors are so helpful.

Be interested in the person

family
friends
knowledge of their condition, including clear limitations

Finding ways they can give back to the community

Acknowledgement of shortended lfe

Labor market inclusion, social inclusion,

I have not had a lot of experience as yet.

Instilling a sense of independence and worth. Helping person be independent with self care as much as possible and also with social activities, school, and work.

individual cognitive behavioral therapy (CBT), engagement in pleasurable activities, engagement in age appropriate and functional activities (e.g., attending school, activities, daily chores, etc.)

Music, participation in decisions

d\ngd\ngdn

OT helps for things like getting out in the world, having confidence, seeking relationships, sex life etc

Encouraging patients to surround themselves with others that share a similar diagnosis(we hold periodic EB family events) and to also surround themselves with those that promote self sufficiency and a positive environment.

It's very important to create a space where they can express their feelings freely, to talk about their fears and strengh. Some times families don't talk about what worries them to " protect" the others, but having a space to share and work on their concerns helps on the self-determinarion

nonpharmacologic pain and itch management skills, changing negative automatic thoughts to more positive and rational thoughts, mindfulness skills

20. Does anybody in your practice evaluate for the spiritual needs of a patient/family coping with EB?

Number of responses: 16

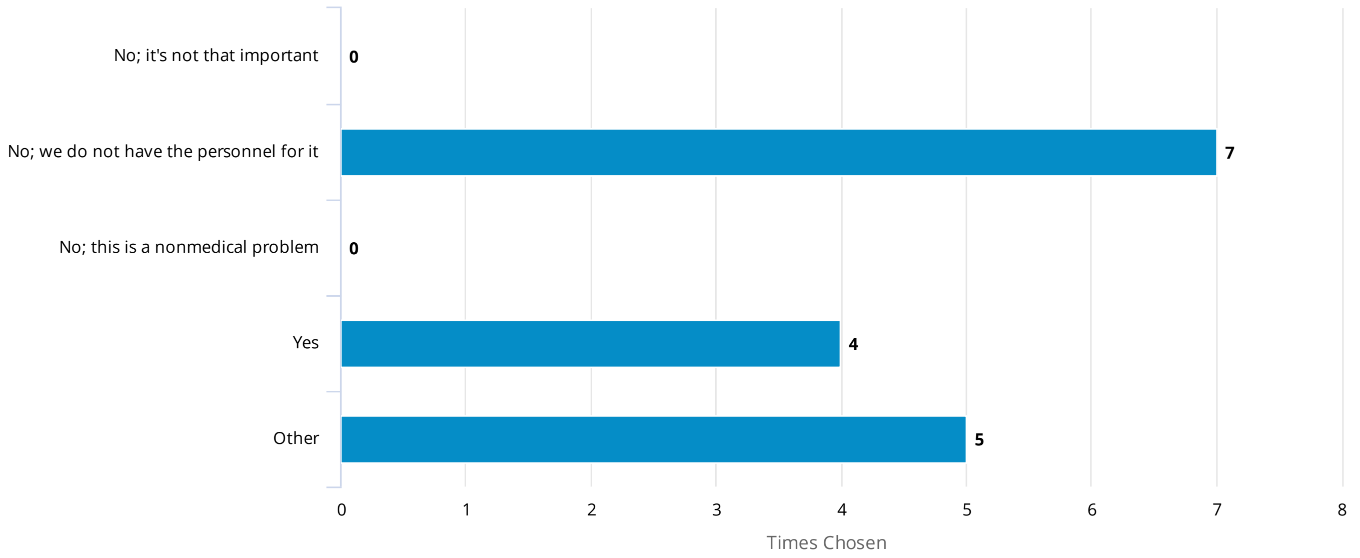

"Other" text answers:

not done though is freely available in our environment

Other

Only if asked.

not sure

I'm not sure if we do

Who does that evaluation?

Number of responses: 6

Text answers:

would depend upon the individual, I would think;

Not sure

ghgdzhgd

case management at least asks about it and can refer

Social worker

The spiritual agent

21. Do you actively take into consideration your patients' cultural, spiritual and ethical values in your decisions about their treatment?

Number of responses: 15

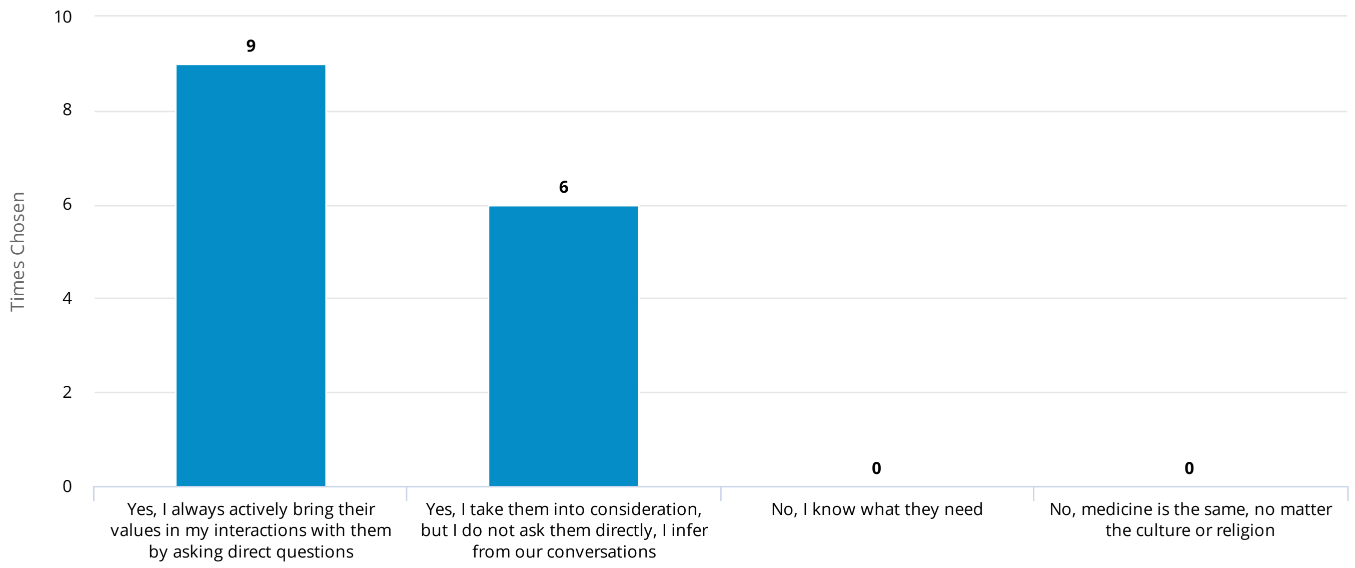

22. In general, how do you measure/define quality of life for your patients?

Number of responses: 15

Text answers:

"Health" and quality of life for each patient differs. We must keep our eyes and ears open to what the families say and patents say and help move them from where they are to greater their potential (not what we think is their potential -we could over or under shoot that). QOL is in their eyes - they may be very happy and content with being with family or others may need to go outside their circle and seek that validation from others. Employment is appealing to some, not all.

iscorEB

by their interactions with others... friends, school mates, family... by asking them directly how they are "feeling"

Ability to participate in activities of daily living like school.

invidivual discussion, no two patients are the same

Participation in family social routine, community acceptance and social inclusion within the limitations imposed by EB

I ask them

Some one who has goals.

self-report

How she and family is doing on a daily basis

zddd

by pain/itch levels, ability to do own ADLs and accomplish their own goals

I'm not sure to be honest

It's very dificult to evaluate, we need to work on a tool to evaluate qol in children

Quality of life for my patients is what THEY say it is as it is different for everyone.

23. Do you find sometimes that your own cultural, spiritual, and/or ethical values interfere with the care of patients living with EB when assessing quality of life/therapy efficacy?

Number of responses: 15

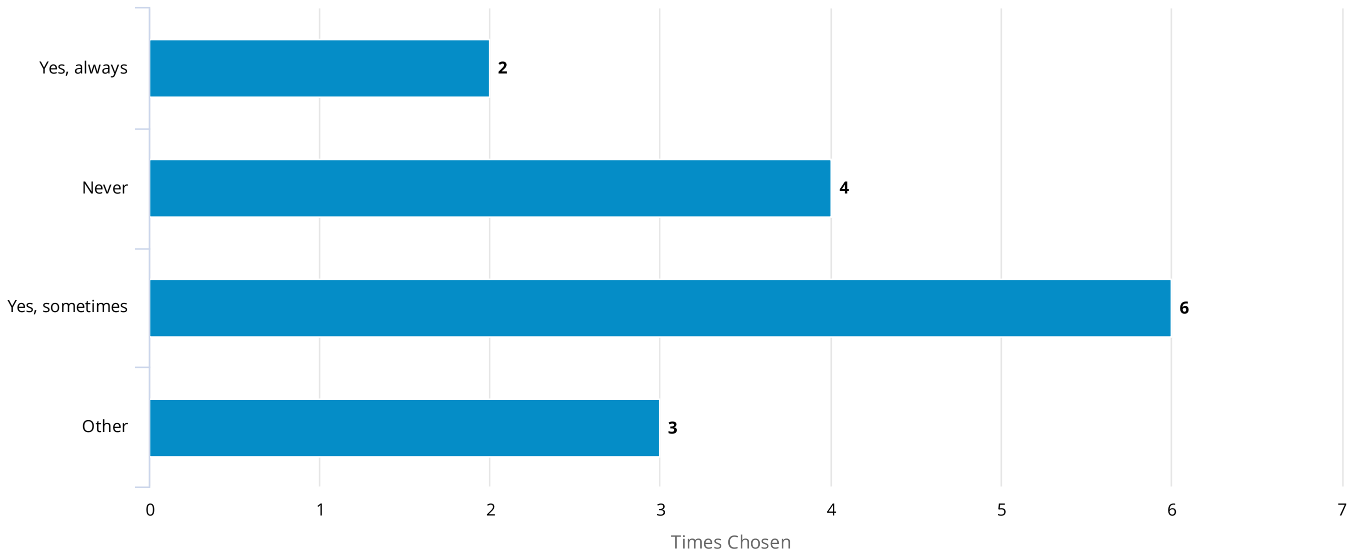

"Other" text answers:

use clinical supervison to explore this

Not openly

not usually

24. Do you, or someone on your team, talk with the unaffected family members (e.g., siblings, sons/daughters, extended family members, etc.) about EB with respect to Palliative Care (including end-of-life care)?

Number of responses: 16

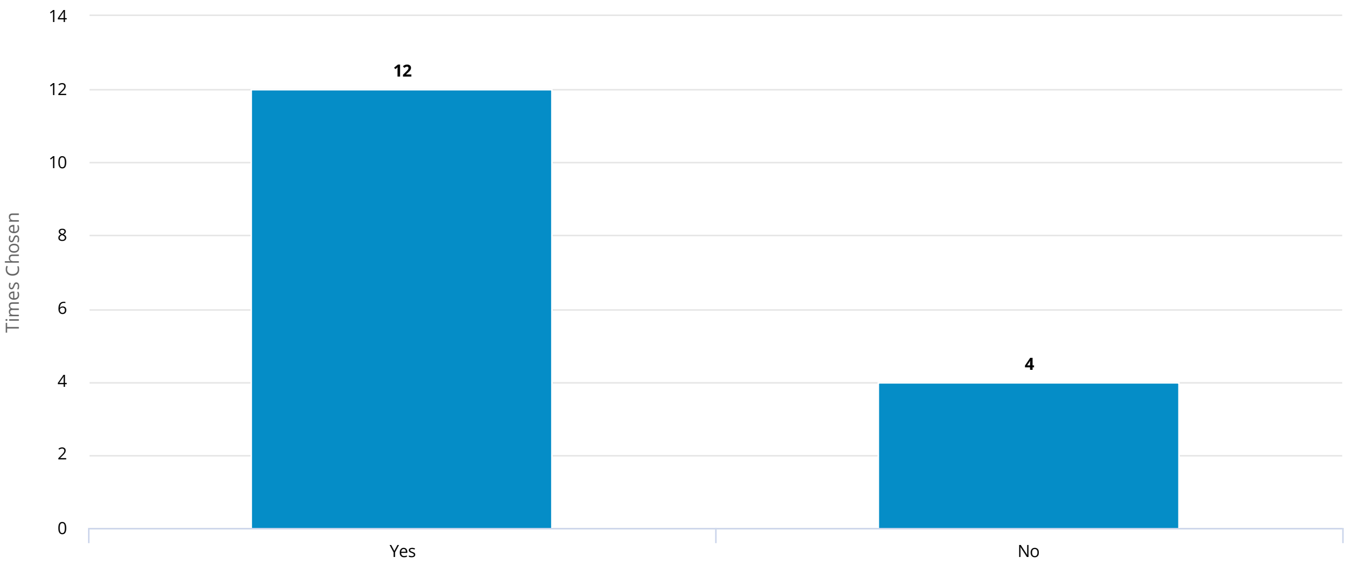

What aspect(s) do you think you/your team do well when working with people living with EB?

Number of responses: 12

Text answers:

We do our best to look into their world, listen to their hopes and what they wish to accomplish, how they want to live their lives. We help them develop plans and then work with them to meet their needs.

no

engaging all members involved in care, family members, etc

patient centered care

Much talk to convince this need, demonstrate the importance of palliative care for quality of life of patients with EB

Advocation and support

multi-disciplinary approach to ALL aspects of the patient's life

Support

jhfjfj

balance independence of HC decision making and respect for their wishes with providing accurate medical advice and treatments when available

Everyone is importabt for us, tha direct family, the extense family, schooling, friends... Every situation is different and we try to adapt to each situation

Wound management education, full service care with all providers meeting the patient all at one clinic visit so there is a need for fewer appointments.

Is there anything that you think that you/your team could do better in this aspect?

Number of responses: 15

Text answers:

Heavier involvement of psychology and psychiatry (resources are limited). Encourage initiation of counseling much earlier than middle school years -family counseling as well.

no

care coordination

bring in palliative care team sooner

introduce palliative earlier, better conversations about this aspect

Orientation, family persuasion and community orientation

Always - you can always do better

I think that looking at supporting the patient in spiritual matters is something to be considered.

better resources and ensure follow through with recommendations

Not sure

zjzjkf

oh yes, we could do everything better, its draining on us as HCPs, sometimes I just can't deal with the constant barrage of patients and phone calls and sick kids/adults, I am sure that affects things

involving the entire family. So far, we have had a hard time including other family members as they don't want or have the time to be present for these discussions. Or even, the patients themselves don't want other siblings involved.

Make more evaluations with patients and families and investigation

Doing more for family support

25. As a medical provider working with people who have EB, do you believe that you are best placed to have in depth conversations about palliative and end-of-life care or are you too close to the patients for difficult conversations to be effective?

Number of responses: 15

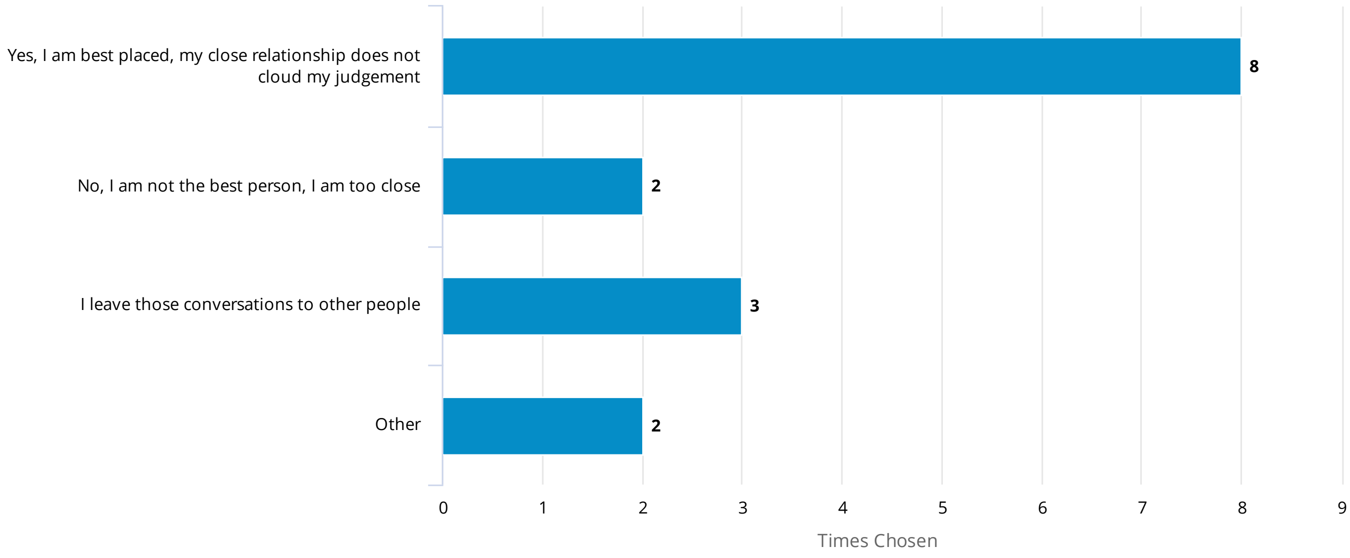

"Other" text answers:

Collaboration with palliative team member would be ideal

i can bring it up but i prefer someone help me and take it further

26. Do you talk about death/dying as a routine part of an ongoing conversation with your patients who live with EB.

Number of responses: 16

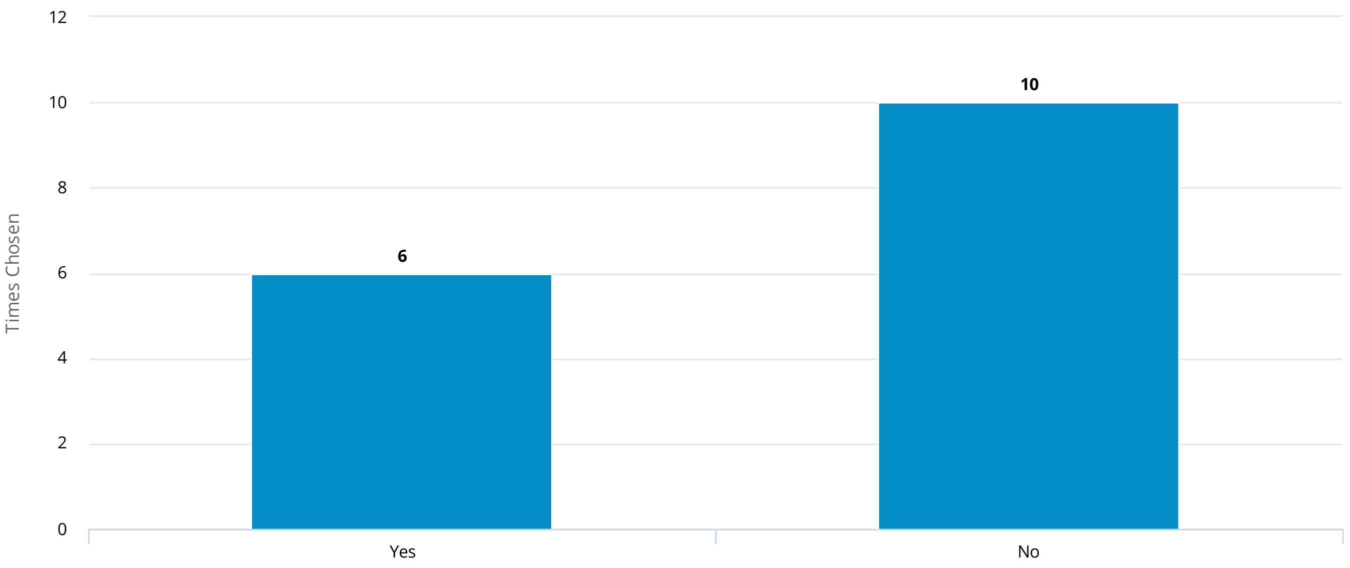

When do you talk with them about death and dying?

Number of responses: 6

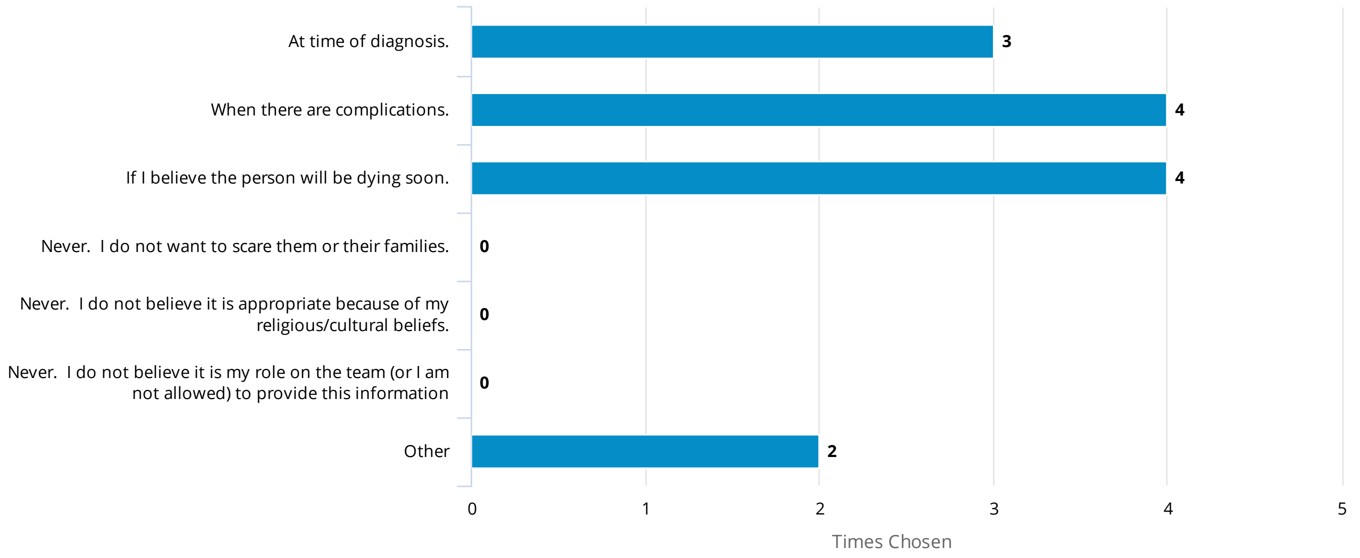

"Other" text answers:

Depending on the EB type, many children living with EB can become adults so there's no nees to talk about dying from the first day, it deppends on how the family integrates the information and how severe is the illness

When the patient or family brings it up

27. Do you talk with your patients living with EB about implementing any of the following into their plan of care?

Number of responses: 15

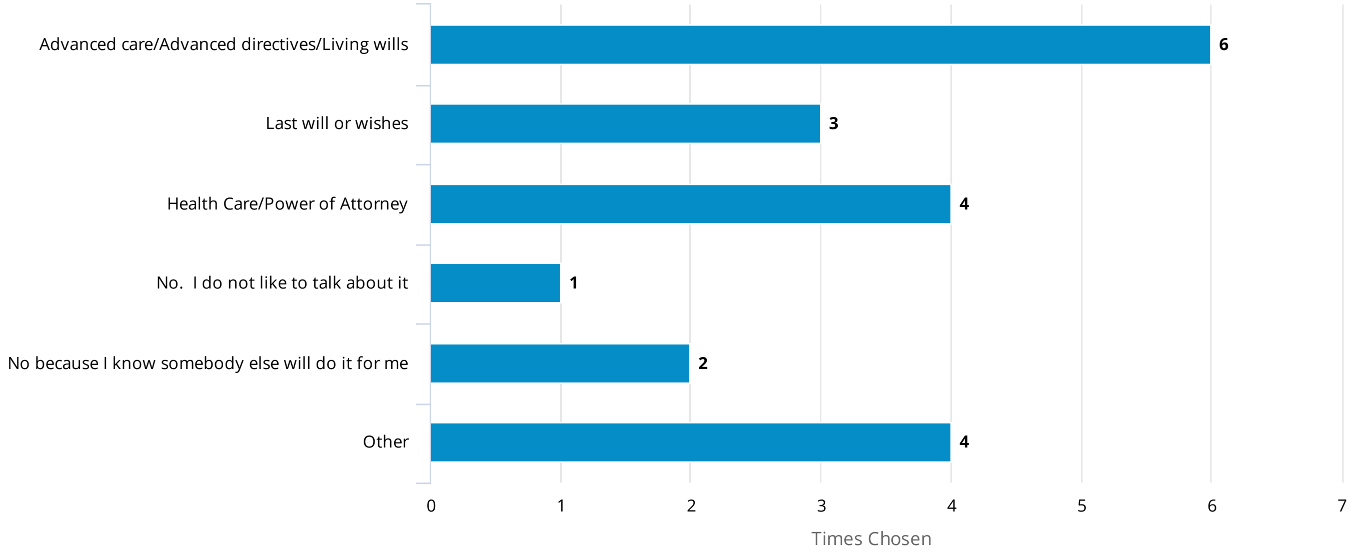

"Other" text answers:

It varies with age, but we do touch on these topics. Not enought though! Pychology and Social work assist.
I have never done this but will now think about such depending on the type, case, etc
not routinely
If initiated by patient/family

28. What do you do or encourage your patients living with EB and their families to do, if anything, related to legacy building?

Number of responses: 14

Text answers:

no
engaging, educating others
I have not dealt with this
at end of life but not routinely
Live well within the constraints imposed by EB, do not create psychosomatic limitations and build your life like any other citizen.
Nothing.
I do not address this.

Nothing in outpatient setting. Inpatient families have access to this through Child Life

Not sure, we are very involved with DEBRA

y46y46y

i don't

I personally have not done any of this.

As we work with children we don't have this situations

I try to have this conversation early, whenever possible, so that the patient and family have time to do the things they want to do before the patient is no longer able to participate.

29. What type of family/friend/other support is available at your institution after the death of a person who had been living with EB?

Number of responses: 15

Text answers:

Institution based we have a bereavement team through pastoral care that remains in contact with the families, services of remembrance are held twice a year.

no

mental health
palliative care folks
spiritual support

reunions

EB CNS has protected time for ongoing bereavement support

Within the limitations of the institution, there are always volunteers who accompany the family after death, with words of encouragement and instructions.

For me - Supervision
For patients family - nothing that I am aware of

Not sure

Bereavement counseling

I am providing private care as an RN

3h53h5h

palliative care has resources

we have chaplains, social workers, counselors

Individual and groip therapy for everyone in the familu who needs that

They can call us, but there is nothing formal other than an annual day of remembrance that is open for families of all patients that have died.

29a) Do you find that level of support to be adequate?

Number of responses: 15

Text answers:

no

no

generally, yes

I haven't had any patients die from EB

yes

No, not yeat

Yes - for me

Not sure

Yes but we are disconnected from it

No

4h4h4

yes mostly

so far yes

Is hard to people living away from the hospital so it could be noce ti have the chance to do therapy at home

No

29b) What, if any, changes would you like to see made that would best support families of people with EB who have died?

Number of responses: 15

Text answers:

continued ongoing outreach - although some families need ot walk away and not look back - hard ot know sometimes what is best.

I do not know that is available.

maintaining contact with those families

no specific

better input from Debra to acknowledge the life lived - as they used to

Specialized care centers distributed by the regions of the country, which can provide this life and death support to the family.

Unsure

Our MDT should have a component of palliative care that each member is aware of and invested in

Unsure

Better MD primary care

2hhh

long term support measures

is there a support group specifically for families who have lost a child or family member to EB? At our institution, we only have lost 4 patients to EB in the last 6-7 years.

Home asistance

Offering family therapy, individual and family grief therapy

40. What do you wish you would have known or learned about Palliative Care during your training?

Number of responses: 15

Text answers:

Anything - I learned it all on the job and while working with our Palliative Care Team here at the institution.

no

that it existed at all

The extent to which palliative care can be involved.

enhanced communication skills

I would like to deepen my knowledge in supporting patients with pain and complications at the time of death.

Palliative care is not addressed in my DEBRA training. I have done self directed additional training through my hospital job and work in palliative care as a district nurse.

What is available. What are the best ways to engage patient in these discussions.

how to initiate conversation about role of palliative that is not soley focused on death & dying

None

2hhj26h

anything I don't think i knew what it was

how to help patients understand the role of palliative care for chronic illness.

Emotional comunication

Everything! Everything that I have learned has come from additional training on my own after graduation.

41. What group(s) of people should be the focus of education resulting from this guideline?

Number of responses: 16

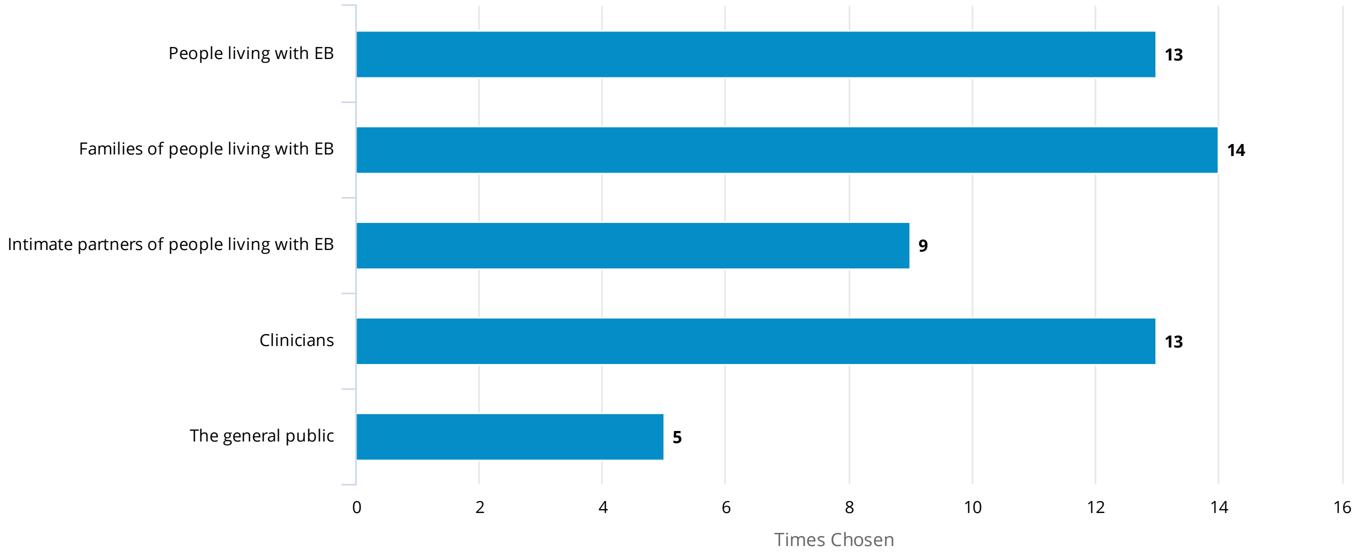

42. Is there anything else regarding Palliaitve Care for people living with EB that you would like us to know or consider?

Number of responses: 14

Text answers:

I think it is critical to include support for the families, post passing of an EB patient.

I don't think so

No

no

Nothing else

Not that I can think of

no

Support for families that are in denial/resistant

MD training

ntnytn

not sure

no

The guilt of the parents

no

43. If you are happy to share with this CPG panel, please upload any local Palliative Care policy or guidance documents you are using here:

Number of responses: 0

No answers collected yet

Please enter your contact details:

Number of responses: 8

First and last name	Title	City and country	Institution	Prefered Email address for contact
Moise Levy	MD	AUSTIN TX USA	Dell Medical School, University of Texas at Austin; Dell Children's Medical Center	mllevytx@gmail.com
Katrin Osti	Nurse	Blumenau Brazil	Debra Brasil	kao@furb.br

First and last name	Title	City and country	Institution	Prefered Email address for contact
Mo Blishen	EB Clinical Nurse	Wellington, New Zealand	DEBRA	mo.blishen@debra.org.nz
Jennifer Chan	occupational therapist, hand therapist	Menlo Park, CA/USA	Lucile Packard Childrens Hospital Stanford	jennifer.chan@sbcglobal.net
Jessica Collins	Psy. D.	Philadelphia, USA	Children's Hospital of Philadelphia	collinsj3@email.chop.edu
Ellen Wallace	RN	Gulf Shores USA	Private Duty	ellewa52@gmail.com
Kellie Badger	RN -EB	Phoenix, AZ USA	Phoenix Children's Hospital	kbadger@phoenixchildrens.com
Eduard Pellicer Arasa	Mr	Barcelona, Spain	Sant Joan de Deu Hospital	epellicera@sjdhospitalbarcelona.org
