# Supplementary material for: Consensus-based guidelines for the provision of palliative and end-of-life care for people living with epidermolysis bullosa
Source: Orphanet J Rare Dis. 2023 Sep 4;18:268. doi: 10.1186/s13023-023-02870-8 (PMC10476410; doi:10.1186/s13023-023-02870-8)
Supplement: Supplementary file 3 — Additional file 3: Stakeholder involvement, peer review, and methodology. [file 13023_2023_2870_MOESM3_ESM.docx]

**Consensus-based Guidelines for the Provision of Palliative and End-of-Life Care for People Living with Epidermolysis Bullosa**

**Supporting Information**

Mark P. Popenhagen^1, 2*^,

Paola Genovese^3^,

Mo Blishen^4^,

Dilini Rajapakse^5^,

Anja Diem^6^,

Alex King^7^,

Jennifer Chan^8^,

Eduard Pellicer Arasa^9^,

Simone Baird^10, 11^,

Anna Carolina Ferreira da Rocha^12,13^,

Gideon Stitt^14^,

Kellie Badger^3^,

Vlasta Zmazek ^15, 16^,

Faiza Ambreen^17, 18^,

Caroline Mackenzie^19^,

Harper Price^3^,

Toni Roberts^20, 21^,

Zena Moore^22^,

Declan Patton^22^,

Paul Murphy^22^,

Kattya Mayre-Chilton^23, 24^

**Supporting Information: Stakeholder involvement, peer review, and methodology**

**A. Stakeholder involvement and peer review**

These guidelines were guided by expert consensus of an international panel formed with diverse role representation including both clinical professionals supporting people with EB and patient population involvement (PPI), members living with EB, and/or PPI members who are caring for, or cared for, an individual with EB (Table 1). All panel members completed written conflict of interest and code of conduct declaration. An independent review panel, blinded to the authors’ names and affiliations, was invited to give feedback that was then incorporated into the manuscript before submission for publication (Table 1).

Table 1. Author and Review Panel Membership

**CG Working Panel**

| **Panel Member** | **Country of origin** | **Specialty with EB** | **Role on panel** | **First meeting** | **Searches** | **Paper filtration and appraisal** | **Outcome summaries** | **Recommendation Meeting** | **Writing** | **Panel review** | **Incorporation of panel feedback** | **Incorporation of reviewers’ feedback** | **Submitting the CG** |
| --- | --- | --- | --- | --- | --- | --- | --- | --- | --- | --- | --- | --- | --- |
| M. Popenhagen | USA | Paediatric Psychologist specializing in Pain and EB; Guideline methodologist | Lead | X | X | X | X | X | X |  | X | X | X |
| P. Genovese | USA | Palliative Care and Pain Management Physician | Co-lead |  | X |  | X | X | X |  |  |  |  |
| F. Ambreen | Pakistan | DEBRA Pakistan; PPI - mother of baby with EB who passed away | Panel | X | X |  |  |  |  |  |  |  |  |
| E.P. Arasa | Spain | Social Worker | Panel | X | X | X |  | X | X |  |  |  |  |
| K. Badger | USA | EB Nurse | Panel | X | X | X |  | X | X |  |  |  |  |
| S. Baird | Australia | DEBRA Australia; PPI | Panel | X | X |  |  | X | X |  |  |  |  |
| M. Blishen | NZ | EB Clinical Nurse; DEBRA New Zealand | Panel | X | X | X |  | X | X |  |  |  |  |
| J. Chan | USA | Occupational Therapist | Panel | X | X | X |  | X | X |  |  |  |  |
| A.C.F. da Rocha | Brazil | PPI | Panel | X |  |  |  | X |  |  |  |  |  |
| A. Diem | Austria | EB Senior Physician (EB house Austria), specializing in Pain, Palliative Care, Emergency Medicine, Genetics | Panel | X | X | X |  | X | X |  |  |  |  |
| A. King | USA | Occupational Therapist | Panel | X | X | X |  | X | X |  |  |  |  |
| C. Mackenzie | UK | EB Clinical Nurse Specialist | Panel | X | X |  |  |  |  |  |  |  |  |
| K. Mayre-Chilton | UK | DI CG Coordinator, Specialist adult research dietitian | Project Manager; Panel |  | X | X |  |  | X |  |  |  | X |
| Z. Moore | Ireland | Professor, Academic School of Nursing & Midwifery | Second appraiser for articles |  |  | X |  |  |  |  |  |  |  |
| P. Murphy | Ireland | Information Specialist; RCSI Library | Information Specialist |  | X |  |  |  |  |  |  |  |  |
| D. Patton | Ireland | Professor, Director of Nursing & Midwifery Research | Second appraiser for articles |  |  | X |  |  |  |  |  |  |  |
| H. Price | USA | Dermatologist, EB Physician | Panel | X | X | X |  |  | X |  |  |  |  |
| D. Rajapakse | UK | Palliative Care, Pain, EB Paediatrician | Panel | X | X |  |  | X | X |  |  |  |  |
| T. Roberts | South Africa | PPI, co-founder DEBRA South Africa | Panel | X | X | X |  |  |  |  |  |  |  |
| G. Stitt | USA | Clinical Pharmacist | Panel |  | X | X |  | X |  |  |  |  |  |
| V. Zmazek | Croatia | DEBRA Croatia; PPI - mother of young man with EB who passed away | Panel | X |  |  |  |  |  |  |  |  |  |
| Key: CG – Consensus Guideline; EB – Epidermolysis Bullosa; DI – DEBRA International; PPI – Patient and Public Involved (People living with EB and carers of people living with EB); UK – United Kingdom; USA – United States of America | | | | | | | | | | | | | |

| **Reviewer Panel** | | | | | | | | | | | | | |
| --- | --- | --- | --- | --- | --- | --- | --- | --- | --- | --- | --- | --- | --- |
| M. Belen | Argentina | PPI – mother of girl with RDEB | Reviewer |  |  |  |  |  |  | X |  |  |  |
| J. Fellows | UK | Consultant Clinical Psychologist | Reviewer |  |  |  |  |  |  | X |  |  |  |
| M.T. Garcia Romero | Mexico | Paediatric Dermatology and researcher | Reviewer |  |  |  |  |  |  | X |  |  |  |
| K. Goldschneider | USA | Paediatric Anesthesiologist and pain specialist | Reviewer |  |  |  |  |  |  | X |  |  |  |
| R. Jones | UK | Specialist EB paediatrics dietitian | Reviewer |  |  |  |  |  |  | X |  |  |  |
| L. Mosley | USA | Anesthesiologist | Reviewer |  |  |  |  |  |  | X |  |  |  |
| J.M. Phillips | USA | Clinical Dietitian II | Reviewer |  |  |  |  |  |  | X |  |  |  |
| N. Yerlett | UK | Specialist EB paediatrics dietitian | Reviewer |  |  |  |  |  |  | X |  |  |  |
| Key: EB – Epidermolysis Bullosa; RDEB – Recessive dystrophic EB; UK – United Kingdom; USA – United States of America | | | | | | | | | | | | | |

**B. Methodology**

**Phase 1:** In 2018, Debra International (DI) consulted with the international EB community and identified palliative care in EB as a high priority area for development. In the same year, an international multidisciplinary team (MDT) panel was established to undertake the guideline development process.

**Phase 2: Survey for EB patients and carers and for international clinical experts.** Electronic surveys were circulated via DEBRA International newsletter, DEBRA-associated social media sites, EB Clinet (a clinical network that encourages collaboration between healthcare professionals working with EB), and DEBRA United States. Utilizing the Delphi technique^(1)^, the panel developed a questionnaire that sought the opinions of people living with EB, their carers, and medical providers who treat EB on the topic of EB and palliative care

**Phase 3: Primary Panel Meeting.** The first face to face panel meeting was held in London, United Kingdom (January 2020) at the first World Congress for EB conference. Using the specific population (P), intervention (I), comparison(C), and outcome (O), to formulate three clinical questions and priority ranking method^(2)^, three broad clinical questions and six outcomes (Symptom Control, Quality of Life, Survivorship, Satisfaction, Grief, and Mental Health/Well-being) were developed as defined below, and served as the basis of the systematic search terms.

**Outcomes Definitions**

**Symptom Control/Management** – This outcome focused on whether the article aimed to evaluate the control or management of symptoms associated with EB, including pain and itch, wounds, infections, dental caries, microstomia, malnutrition, and any other symptom that is associated with the condition of EB. While the symptoms did not have to be exclusive to EB, they did need to occur as a direct result of EB.

**Survivorship** – Survivorship is living with, and in spite of, EB that would begin as soon as a diagnosis is made, typically shortly after birth. Like in the cancer literature, the use of the term “co-survivor” means a person who cares/cared for a person with EB.

**Mental Health/Well-being** – According to the WHO^(3)^, “Mental health is a state of mental well-being that enables people to cope with the stresses of life, realize their abilities, learn well, and work well, and contribute to their community. It is an integral component of health and well-being that underpins our individual and collective abilities to make decisions, build relationships and shape the world we live in.” Therefore, this outcome explored the mental health stressors or resilience factors in those living with EB and discussed the strategies that the individual with EB might use to cope with the condition.

**Quality of Life** (QoL) for person with EB and/or carers – This CG followed the WHO’s definition of QoL ("an individual's perception of their position in life in the context of the culture and value systems in which they live and in relation to their goals, expectations, standards and concerns") for those who have EB and for their carers.”^(4)^

**Life satisfaction** – Satisfaction explores whether the articles touch on measuring or otherwise assessing the culture, values, beliefs, religion, and/or spirituality of the person living with EB as defined by the person living with EB.

**Grief** – Grief is an individual reaction to loss. However, loss does not have to exclusively mean death, but rather can be related to a loss of ability, plans/planned activities, previous skills, independence, dignity, a caregiver, identity, or any other loss that leads to an emotional impact on an individual with EB or their carer. Bereavement differs from loss in that it is a period of mourning or intense grief, often after the death of a loved one. Articles may have also considered grief that is uncomplicated (“typical grief”) and/or complicated (grief that goes on longer than a few months, worsens over time, has an absence of mourning, extreme focus on the object that is being grieved, extreme focus on that which has been lost, difficulties focusing or doing normal activities, etc.).

**Phase 4: Systematic Literature Searches**

For the systematic literature search, the search terms included the following:

Keyword list used for literature search

| - Epidermolysis Bullosa - Palliative Care - Quality of life - End-of-life - EB - DDEB - RDEB - DEB - Dystrophic epidermolysis bullosa - Psychosocial impact of chronic disease - EB-Simplex - Junctional EB - Herlitz - non-Herlitz - Kindler - Generalized severe epidermolysis bullosa - Skin conditions - Death | - Palliative medicine - Palliative therapy - Supportive care - Skin fragility disorders - Caregiver stress - Rare diseases - Butterfly skin disease - Compassionate care - Compassion fatigue - Grief - Complicated grief - Hospice care - Stress management - Decision making - EB multidisciplinary clinic - Palliative wound care - Healthy Grief - Carer fatigue - Care burnout - Burden of Care + EB, EB + multidisciplinary | - EB + missed opportunities - EB + home care - EB + hospital care - EB care/treatment in rural areas - EB care/treatment in underdeveloped countries - EB care/treatment in developed countries - EB specialty care - EB + community support - EB + community centre - EB national organizations + resources - EB international organizations + resources - Decisions at EoL in severe skin conditions - EB + grief + survivors |
| --- | --- | --- |

EMBASE, PubMed, and CINAHL search engines were conducted by an Information Specialist/Librarian see the database search tables below:

EMBASE, PubMed, and CINAHL Search Tables

|  | **EMBASE on embase.com** |  |
| --- | --- | --- |
| A | Palliative care |  |
| 1 | 'epidermolysis bullosa':ti,ab,kw OR 'epidermolysis bullosa'/exp OR “skin fragility disorders” OR “butterfly skin disease” | 9004 |
| 2 | palliative:ti,ab,de,kw OR 'palliative therapy'/exp OR 'palliative nursing'/exp OR 'palliative wound care' OR 'end of life' OR 'quality of life' OR 'quality of life'/exp OR 'supportive care' OR 'psychosocial care'/exp | 778749 |
| 3 | #1 AND #2 | 387 |
|  |  |  |
| B | Carers, stress, and grief |  |
| 1 | 'epidermolysis bullosa':ti,ab,kw OR 'epidermolysis bullosa'/exp OR “skin fragility disorders” OR “Butterfly skin disease” | 9004 |
| 2 | “carer fatigue” OR “carer burnout” OR “caregiver fatigue” OR “Caregiver stress” OR 'caregiver burnout'/exp OR “Care burnout” OR “Burden of Care” OR “Compassionate care” OR “Compassion fatigue” OR ‘Grief’/exp OR “complicated grief” OR 'complicated grief'/exp OR 'anticipatory grief'/exp OR “healthy grief” OR “psychosocial impact” | 21059 |
| 3 | #1 AND #2 | 9 |
|  |  |  |
| C | Settings |  |
| 1 | 'epidermolysis bullosa':ti,ab,kw OR 'epidermolysis bullosa'/exp OR “skin fragility disorders” OR “Butterfly skin disease” | 9004 |
| 2 | hospital:ti,ab,de,kw OR hospitals:ti,ab,de,kw OR care OR 'hospital care'/exp OR 'home care' OR 'home care'/exp OR 'community care*' OR 'community support' OR 'community center' OR 'specialty care' OR 'primary care' OR 'hospice care' OR 'multidisciplinary clinic' OR 'rural area*' OR 'developing countries':ti,ab,kw OR 'developing country'/exp | 6025898 |
| 3 | #1 and #2 | 1277 |
| **Limit** | AND ('article'/it OR 'article in press'/it OR 'review'/it) AND ('case report'/de OR 'clinical article'/de OR 'clinical protocol'/de OR 'clinical study'/de OR 'cohort analysis'/de OR 'comparative study'/de OR 'controlled study'/de OR 'human'/de OR 'longitudinal study'/de OR 'major clinical study'/de OR 'multicenter study topic'/de OR 'observational study'/de OR 'practice guideline'/de OR 'prospective study'/de OR 'qualitative research'/de OR 'retrospective study'/de OR 'systematic review'/de) | 839 |

|  | **PubMed** |  |
| --- | --- | --- |
| **A** | **Palliative care** |  |
| 1 | "Epidermolysis Bullosa Acquisita"[MeSH Terms] OR "Epidermolysis Bullosa Dystrophica"[MeSH Terms] OR "epidermolysis bullosa, junctional"[MeSH Terms] OR "Epidermolysis Bullosa Simplex"[MeSH Terms] OR "Epidermolysis Bullosa"[MeSH Terms] OR "Epidermolysis Bullosa"[Title/Abstract] | 6107 |
| 2 | ("Hospice and Palliative Care Nursing"[Mes h] OR "Palliative Medicine"[Mesh] OR "Palliative Care"[Mesh] OR "Hospice Care"[Mesh]) OR ("Terminal Care"[Mesh] OR "terminal care"[Title/Abstract] OR palliative[Title/Abstract] OR "palliative wound care"[Title/Abstract] OR "end of life"[Title/ABstract] OR "quality of life"[Title/Abstract] OR "supportive care"[Title/Abstract] OR "psychosocial care"[Title/Abstract]) | 778749 |
| 3 | #1 AND #2 | 162 |
|  |  |  |
|  | **Carers, stress and grief** |  |
| 1 | "Epidermolysis Bullosa Acquisita"[MeSH Terms] OR "Epidermolysis Bullosa Dystrophica"[MeSH Terms] OR "epidermolysis bullosa, junctional"[MeSH Terms] OR "Epidermolysis Bullosa Simplex"[MeSH Terms] OR "Epidermolysis Bullosa"[MeSH Terms] OR "Epidermolysis Bullosa"[Title/Abstract] | 6107 |
| 2 | “carer fatigue” [Title/Abstract] OR “carer burnout” [Title/Abstract] OR “caregiver fatigue” [Title/Abstract] OR “Caregiver stress[Title/Abstract]” OR “caregiver burnout” [Title/Abstract] OR “Carer burnout” [Title/Abstract] OR “Burnout, Psychological”[Mesh] OR “Burden of Care” [Title/Abstract] OR “Compassionate care” [Title/Abstract] OR “Compassion fatigue” [Title/Abstract] OR “Grief”[Mesh] OR “complicated grief” [Title/Abstract] OR “anticipatory grief” [Title/Abstract] OR “Disenfranchised Grief”[Mesh] OR “healthy grief” [Title/Abstract] OR “psychosocial impact” [Title/Abstract] | 26061 |
| 3 | #1 AND #2 | 6 |

|  | **CINAHL via EbscoHost** |  |
| --- | --- | --- |
| 1 | TI ‘epidermolysis bullosa’ OR AB ‘epidermolysis bullosa’ OR (MH “Epidermolysis Bullosa”) | 694 |
| 2 | TI palliative OR AB palliative OR (MH “Palliative Care”) OR (MH “Hospice and Palliative Nursing”) OR (MH “Terminal Care”) OR (MH “Hospice Care”) | 59,509 |
| 3 | 1 AND 2 | 12 |

Articles and conference abstracts were not limited based on language, year published, journal published in, author’s or study’s country of origin, or EB type. Articles published in lay media (e.g., newspapers, magazines, blog posts, etc.) were not included. Although the initial search ended on 15 June 2020, relevant articles discovered by the panel that were published during the writing of this guideline (through June 2022) and one recommended through the journal peer reviewer were also included.

**Phase 5: Article Appraisals and GRADEpro summary tables**

Full-text articles were then obtained for the remaining articles and were first appraised by MP, HP, AD, CM, AK, EPA, MB, JC, KB, GS, TR, and KMC. The appraisal tool was modified from CASP and GRADE terms, see appraisal tool.

**Article Appraisal Tool**

Two research specialists, DP and ZM, conducted a second appraisal to ensure consistency. Four CPGs were reviewed by MP using the Appraisal of Guidelines for Research and Evaluation II instrument (AGREE II)^(5)^. While most of the articles were published in English, the authors also identified relevant articles written in Dutch, French, German, and Spanish. Due to a limited number of available reviewers fluent in these languages, these articles had only one reviewer. Articles were declined if they did not meet the criteria of not directly address one or more of the clinical outcomes; if they were associated with, or better addressed by, another CPG that was complete at the time of article collection (e.g., pain management, skin and wound care, pregnancy, sexuality, foot care). We refer the reader to those guidelines for specific information on those topics. A total of 85 articles were used in the qualitative synthesis of this CG.

During the review process, each included article was grouped into one or more of the clinical outcome categories, and then were graded using GRADE^(6)^ methodology, and summarized in the GRADEpro^(7)^ “Summary of Findings Table” below. The articles identified in this CG cover a multitude of research types (e.g., single case studies, clinical practice guidelines, literature reviews, control matched, expert opinion, grey literature, etc.) and participant types (people living with EB, carers, providers).

| **Certainty assessment** | | | | | | | **Impact** | **Certainty** | **Importance** |
| --- | --- | --- | --- | --- | --- | --- | --- | --- | --- |
| **№ of studies** | **Study design** | **Risk of bias** | **Inconsistency** | **Indirectness** | **Imprecision** | **Other considerations** |  |  |  |
| **Symptom Control/Management – Acute medical needs (e.g. pain, hydration, oral intake); Chronic needs (e.g. respiratory, fatigue); and/or Psychological (e.g. depression, anxiety, post-traumatic stress disorder)** | | | | | | | | | |
| 44 | observational studies | serious^a^ | not serious^b^ | not serious | not serious^c^ | none^d^ | 1545 total participants were identified. Of these: 911 participants were people living with EB (8 of whom were discussed in a case study); 10 participants identified as parents/caregivers; 56 participants were physicians; and 10 articles did not identify the number of participants or the number was not applicable to the article. One article included 3 "hypothetical" children with EB who, because they were not real, were not counted in the total number of participants. Also of note, the identified articles cover a multitude of research types (e.g., single case studies, clinical practice guidelines, literature reviews, control matched, expert opinion, grey literature, etc.) and participant types (people living with EB, carers, providers). Because EB is classified as a rare disease and there are few people living with the disease, it is very likely that the same participants were used, or otherwise included, across the identified articles. As such, caution should be exercised when considering the number of participants. | ⨁◯◯◯ Very low | CRITICAL |
| **Quality of Life (QOL) for person with EB and/or carers** | | | | | | | | | |
| 76 | observational studies | serious^e^ | serious^f^ | not serious | not serious^g^ | none^d^ | 4780 total participants were identified. Of these: 4535 participants were people living with EB (7 of whom were discussed in a case study); 189 participants were parents/caregivers; 56 participants were physicians; and 20 articles did not identify the number of participants or the number was not applicable to the article. One article included 3 "hypothetical" children with EB who, because they were not real, were not counted in the total number of participants. Also of note, the identified articles cover a multitude of research types (e.g., single case studies, practice guidelines, cross-sectional studies, literature reviews, expert consensus, grey literature, etc.) and participant types (people living with EB, carers, providers). Because EB is classified as a rare disease and there are few people living with the disease, it is very likely that the same participants were used, or otherwise included, across the identified articles. As such, caution should be exercised when considering the number of participants. | ⨁◯◯◯ Very low | CRITICAL |

| **Certainty assessment** | | | | | | | **Impact** | **Certainty** | **Importance** |
| --- | --- | --- | --- | --- | --- | --- | --- | --- | --- |
| **№ of studies** | **Study design** | **Risk of bias** | **Inconsistency** | **Indirectness** | **Imprecision** | **Other considerations** |  |  |  |
| **Survivorship** | | | | | | | | | |
| 24 | observational studies | serious^h^ | serious^i^ | serious | very serious^j^ | none^d^ | 566 total participants were identified. Of these: 329 participants were people living with EB (5 of whom were discussed in a case study); 31 participants were parents/caregivers; and 6 articles did not identify the number of participants or the number was not applicable to the article. Also of note, the identified articles cover a multitude of research types (e.g., single case studies, literature reviews, control matched, expert opinion, etc.) and participant types (people living with EB, carers, providers). Because EB is classified as a rare disease and there are few people living with the disease, it is very likely that the same participants were used, or otherwise included, across the identified articles. As such, caution should be exercised when considering the number of participants. | ⨁◯◯◯ Very low | CRITICAL |
| **Satisfaction that included their culture, beliefs, religion, and/or other measurable outcomes.** | | | | | | | | | |
| 17 | observational studies | serious^k^ | serious^l^ | serious | serious^m^ | none^d^ | 908 total participants were identified. Of these: 887 participants were people living with EB (2 of whom was discussed in a case study); 21 participants were parents/caregivers; 6 articles did not identify the number of participants or the number was not applicable to the article. Also of note, the identified articles cover a multitude of research types (e.g., single case studies, meta-analyses, literature reviews, control matched, expert consensus, retrospective, etc.) and participant types (people living with EB and carers). Because EB is classified as a rare disease and there are few people living with the disease, it is very likely that the same participants were used, or otherwise included, across the identified articles. As such, caution should be exercised when considering the number of participants. | ⨁◯◯◯ Very low | CRITICAL |
| **Grief (e.g., Signs of complicated grief; Coping with grief; Understanding of grief and bereavement)** | | | | | | | | | |
| 3 | observational studies | very serious^n^ | not serious | very serious | very serious^o^ | none^p^ | 28 total participants were identified. Of these: 28 participants were people living with EB but came from a single article; and 2 articles did not identify the number of participants or the number was not applicable to the article. Also of note, the identified articles cover a multitude of research types (e.g., questionnaire development, expert opinion, qualitative study, etc.) and participant types (people living with EB and carers [some carers came from the same family]). EB is classified as a rare disease and there are few people living with the disease, it is very likely that the same participants were used, or otherwise included, across the identified articles. As such, caution should be exercised when considering the number of participants. | ⨁◯◯◯ Very low | CRITICAL |

| **Certainty assessment** | | | | | | | **Impact** | **Certainty** | **Importance** |
| --- | --- | --- | --- | --- | --- | --- | --- | --- | --- |
| **№ of studies** | **Study design** | **Risk of bias** | **Inconsistency** | **Indirectness** | **Imprecision** | **Other considerations** |  |  |  |
| **Mental health/Well-being** | | | | | | | | | |
| 27 | observational studies | very serious^q^ | serious^r^ | serious | very serious^s^ | none^d^ | 1784 total participants were identified. Of these: 1705 participants were people living with EB (3 of whom were discussed in a case study); 79 participants were parents/caregivers; and 7 articles did not identify the number of participants or the number was not applicable to the article. Also of note, the identified articles cover a multitude of research types (e.g., single case studies, literature reviews, qualitative, questionnaires, expert opinion, etc.) participant types (people living with EB and carers). Because EB is classified as a rare disease and there are few people living with the disease, it is very likely that the same participants were used, or otherwise included, across the identified articles. As such, caution should be exercised when considering the number of participants. | ⨁◯◯◯ Very low | CRITICAL |

**CI:** confidence interval

#### Explanations

a. There are more in the 50-75% range (18) than 0-25% range (13) or 100% (10); 3 were undetermined

b. 18 articles <50%, 23 articles 50-75%, 3 articles >75%. Upgrade because 2 clinical practice guidelines that involve symptom control (pain, occupational therapy) were identified.

c. Downgrade for sample size because 25% (11/41) of studies have a n of 1. Downgrade for lack of sufficient numbers of studies with confidence intervals. Downgrade for number of events as there are <100 events even though this analysis includes every article that the body of literature had to offer. Upgrade for inclusion of 4 related CPGs that all scored highly on AGREE II criteria.

d. Unsuspected as the individual studies do not typically have a large number of participants.

e. There are more in the 50-75% range (32) than 0-25% range (21) or 100% (14); 7 were undetermined

f. 21 articles <50%, 42 articles 50-75%, 11 articles >75%

g. Did not downgrade for sample size because only 14% (10/70) of studies have a n of 1 and there was a total of 4780 participants identified. Downgrade for lack of sufficient numbers of studies with confidence intervals. Downgrade for number of events as there are <100 events even though this analysis includes every article that the body of literature had to offer. Upgrade for inclusion of 2 related CPGs that all scored highly on AGREE II criteria.

h. There are more in the 50-75% range (9) than 0-25% range (5) or 100% (5); 2 were undetermined

i. 7 articles <50%, 11 articles 50-75%, 3 articles >75%

j. Downgrade for sample size because 23.8% (5/21) of studies have a n of 1 and because there was only a total of 536 participants identified. Downgrade for lack of sufficient numbers of studies with confidence intervals. Upgrade for inclusion of a related CPG that scored highly on AGREE II criteria. Downgrade for number of events as there are <100 events even though this analysis includes every article that the body of literature had to offer.

k. There are more in the 50-75% range (8) than 0-25% range (3) or 100% (2); 4 were undetermined

l. 5 articles <50%, 11 articles 50-75%, 1 article >75%

m. Did not downgrade for sample size because 25% (1/15) of studies have a n of 1 and there are 908 participants. Downgrade for lack of sufficient numbers of studies with confidence intervals. Downgrade for number of events as there are <100 events even though this analysis includes every article that the body of literature had to offer.

n. There are more in the 100% range (2) than 0-25% range (1) or 25-50% range (0); 2 were undetermined

o. Downgrade for sample size because there were only 28 participants that came from one study. Two studies did not identify the number of participants. Downgrade for lack of sufficient numbers of studies with confidence intervals. Downgrade for number of events as there are <100 events even though this analysis includes every article that the body of literature had to offer.

p. Publication bias undetected as there are not enough studies to determine if there is publication bias.

q. There are more in the 50-75% range (10) and almost as many (8) in the 100% range, than 0-25% range (6); 2 were undetermined

r. 9 articles <50%, 11 articles 50-75%, 6 articles >75%

s. Downgrade for sample size because 11.1% (3/27) of studies have a n of 1 and there are only a total of 1784 participants. Downgrade for lack of sufficient numbers of studies with confidence intervals. Upgrade for inclusion of a related CPG that scored highly on AGREE II criteria. Downgrade for number of events as there are <100 events even though this analysis includes every article that the body of literature had to offer.

**Limitations**

EB is classified as a rare disease and, as a result, producing large, randomized control studies with high levels of confidence is often difficult, at best, in this population. Additionally, it is very likely that the same participants were used, or otherwise included, across several of the identified articles. As such, caution should be exercised when considering the total number of participants across studies. Finally, although this analysis includes every article that the body of EB-specific literature had to offer, there were still too few articles to form recommendations with a high level of confidence and to identify publication bias. Although several other CPGs with large effect size were used, many of the recommendations came from single case studies and other low-level evidence, necessitating significant use of expert panel consensus.

**Phase 6 Recommendation Panel Meeting**

October 9, 2021, the panel had a second group meeting (using the virtual conferencing program Zoom [San Jose, California USA] because of the COVID-19 pandemic; MP, PG, KB, GS, ACFR, AD, MB, DR, SB, AK, EPA, and JC attended) and used the GRADE^(6, 7)^ tool based on findings in the appraised articles, expert opinion, and panel consensus to formulate the recommendations used in this CG.

**Phase 7 Editorial**

The panel addressed all reviewer comments in the final editing stage before submission. The DI coordinator conducted the Appraisal of Guidelines for Research & Evaluation (AGREE II) tool cross check, to increase the quality of practice guidelines in rare diseases. The draft manuscript was reviewed by all the panel, and their input was included. MP submitted the article for journal peer review process and addressed all reviewer and editor comments in the final edition.

**References**

1.	James D, Warren-Forward H. Research methods for formal consensus development. Nurse Researcher. 2015;22(3):35-40.

2.	Sackett D. Evidence-based medicine. Semin Perinatol. 1997;21(1):3-5.

3.	World Health Organization. Mental health: strengthening our response 2022. <https://www.who.int/news-room/fact-sheets/detail/mental-health-strengthening-our-response>. Accessed 31 July 2022.

4.	World Health Organization. The World Health Organization quality of life (WHOQOL) user manual 2012. file:///C:/Users/106795/Downloads/WHO_HIS_HSI_Rev.2012.03_eng.pdf. Accessed 31 July 2022.

5.	Brouwers M, Kho M, Browman G, Burgers J, Cluzeau F, Feder G, et al. AGREE II: Advancing guideline development, reporting and evaluation in healthcare. Can Med Assoc J 2010;14(182):E839-42.

6.	Mohd Mustapa M, Exton L, Bell H, Ormerod A, Hughes J, Levell N, et al. Updated guidance for writing a British Association of Dermatologists clinical guideline: the adoption of the GRADE methodology 2016. Br J Dermatol. 2017;176:44-51.

7.	Evidence Prime I. GRADEpro GDT: GRADEpro Guideline Development Tool. McMaster University; 2020.
